# Supplementary material for: The Hidden Potential of High-Throughput RNA-Seq Re-Analysis, a Case Study for DHDPS, Key Enzyme of the Aspartate-Derived Lysine Biosynthesis Pathway and Its Role in Abiotic and Biotic Stress Responses in Soybean
Source: Plants (Basel). 2022 Jul 1;11(13):1762. doi: 10.3390/plants11131762 (PMC9269547; doi:10.3390/plants11131762)
Supplement: Supplementary file 1 [file plants-11-01762-s001.zip › plants-1751726-supplementary.pdf]

Table S1. Significant results (E-value < 1e-5) of the HMMER (V3.1b2) analysis with gene model information per result (gene identifier, chromosome, strand, start and stop position gene), according to the Wm82.a2.v1, Mt4.0.v1 and TAIR10 annotation for *G. max*, *M. truncatula* and *A. thaliana* respectively.

Species	Sequence Identifier HMMER	Sequence Identifier this paper	E-value full sequence
Physcomitrella patens	Pp3c5_24750V3.1.p	Pp.DHDPSA1	4.8E-183
Physcomitrella patens	Pp3c6_3950V3.1.p	Pp.DHDPSA2	4.1E-182
Selaginella moellendorffii	179125	Sm.DHDPSA	7.1E-188
Picea abies	MA_116863g0010	Pa.DHDPSA1	6.5E-188
Picea abies	MA_139857g0010	Pa.DHDPSA2	1.2E-186
Sorghum bicolor	Sobic.010G216300.1.p	Sb.DHDPSA1	4.6E-197
Sorghum bicolor	Sobic.006G186600.1.p	Sb.DHDPSA2	6.7E-195
Zea mays	Zm00001d046898_P001	Zm.DHDPSA1	3.40E-196
Zea mays	Zm00001d002602_P002	Zm.DHDPSA2	4.9E-194
Oryza sativa	LOC_Os04g48540.1	Oz.DHDPSA1	2.5E-171
Oryza sativa	LOC_Os04g18200.1	Oz.DHDPSA2	2.8E-198
Aquilegia coerulea	Aqcoe7G063900.1.p	Ac.DHDPSA1	1.2E-198
Aquilegia coerulea	Aqcoe7G343400.1.p	Ac.DHDPSA2	3.0E-198
Vitis vinifera	VIT_215s0048g00750.1	Vv.DHDPSA	1.8E-202
Populus trichocarpa	Potri.002G149500.1.p	Pt.DHDPSA1	1.4E-202
Populus trichocarpa	Potri.014G071100.1.p	Pt.DHDPSA2	5.4E-200
Eucalyptus grandis	Eucgr.D02445.1.p	Eg.DHDPSA1	1.3E-200
Eucalyptus grandis	Eucgr.E01412.1.p	Eg.DHDPSA2	1.0E-152
Eucalyptus grandis	Eucgr.E01411.1.p	Eg.DHDPSA3	2.20E-150
Eucalyptus grandis	Eucgr.E01410.1.p	Eg.DHDPSA4	1.6E-149
Arabidopsis thaliana	AT2G45440.1	AtDHDPS2	8.70E-201
Arabidopsis thaliana	AT3G60880.1	AtDHDPS1	7.50E-199
Lotus japonicus	LotjaGi1g1v0463600.1	Lj.DHDPSA	1.20E-196
Lotus japonicus	LotjaGi3g1v0431700.1	Lj.DHDPSB1	8.60E-177
Lotus japonicus	LotjaGi3g1v0431900.1	Lj.DHDPSB2	1.60E-172
Lotus japonicus	LotjaGi3g1v0431800.1	Lj.DHDPSB3	4.00E-163
Lotus japonicus	LotjaGi4g1v0363100.1	Lj.DHDPSB4	3.20E-159
Medicago truncatula	Medtr1g088775.1	Mt.DHDPSA1	1.70E-198
Medicago truncatula	Medtr7g075090.1	Mt.DHDPSA2	2.50E-197
Medicago truncatula	Medtr8g036040.1	Mt.DHDPSB1	8.30E-181
Medicago truncatula	Medtr8g036035.1	Mt.DHDPSB2	1.50E-176
Medicago truncatula	Medtr8g036020.1	Mt.DHDPSB3	1.20E-172
Medicago truncatula	Medtr8g036050.1	Mt.DHDPSB4	1.10E-160
Pisum sativum	Psat3g114440.1	Ps.DHDPSA	4.70E-198
Pisum sativum	Psat4g149480.1	Ps.DHDPSB1	4.60E-176
Pisum sativum	Psat4g149520.1	Ps.DHDPSB2	8.30E-175
Pisum sativum	Psat4g149440.1	Ps.DHDPSB3	2.20E-170
Phaseolus vulgaris	Phvul.008G075400.1	Pv.DHDPSA	1.90E-197
Phaseolus vulgaris	Phvul.010G085500.1	Pv.DHDPSB	3.50E-180
Vigna unguiculata	Vigun05g079000.1	Vu.DHDPSA	7.70E-197
Vigna unguiculata	Vigun10g113600.1	Vu.DHDPSB	2.80E-181
Glycine max	Glyma.09G268200.1.p	Gm.DHDPSA1	1.70E-198

Glycine max	Glyma.18G221700.1.p	Gm.DHDPSA2	1.80E-197
Glycine max	Glyma.03G022300.1.p	Gm.DHDPSB	1.70E-184

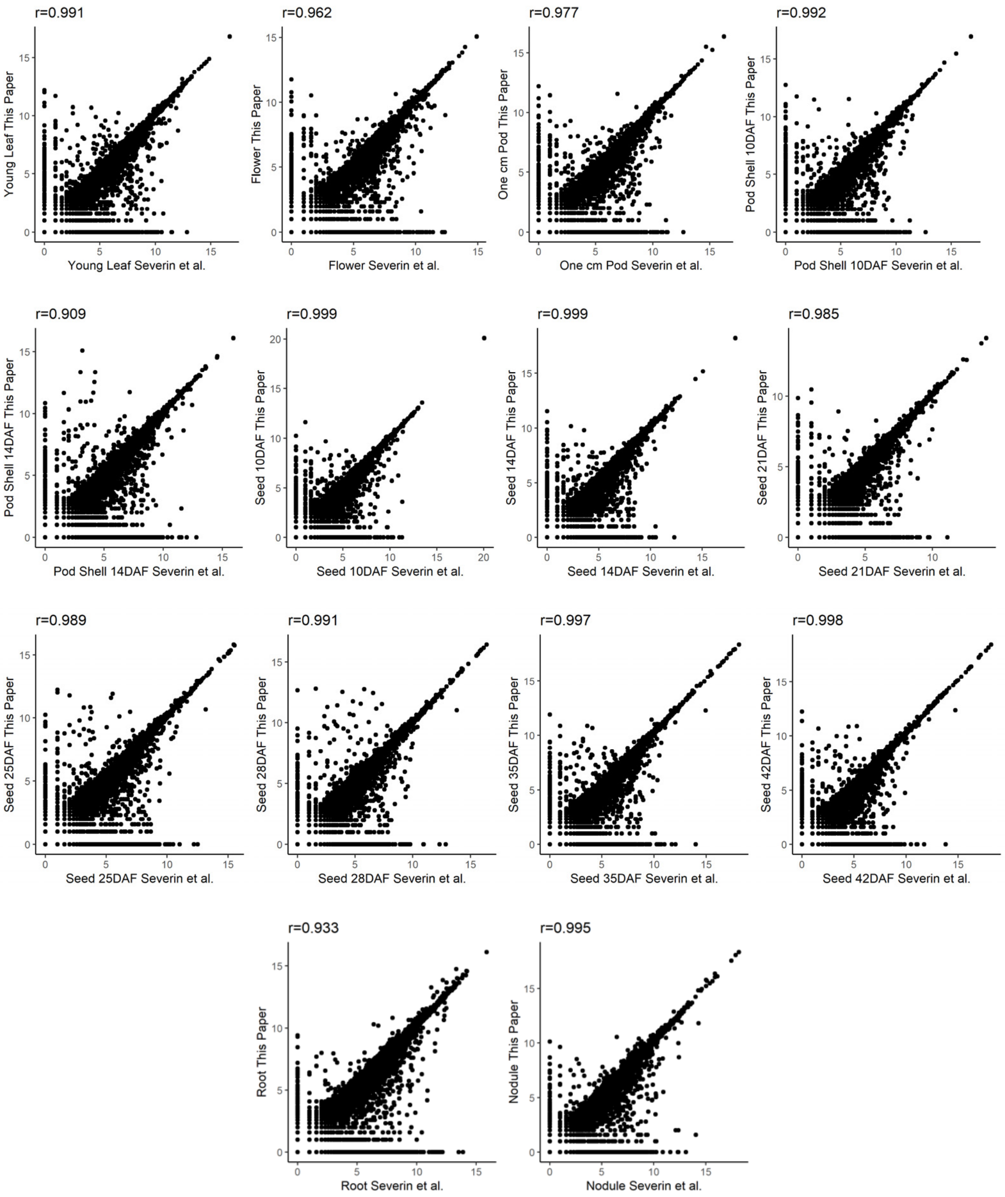

Figure S1. Correlation scatterplots comparing the gene expression counts of the publicly available SoyBase RNA-Seq Atlas data of *G. max* (X-axis) and this paper's results (Y-axis) for the 14 available plant samples [36]. Gene expression counts were log2 transformed and the Pearson's correlation coefficient is given on top of each plot.

Table S2. Experiment list used for RNA-Seq differential expression re-analysis. In total 5 biotic and 10 abiotic SRA studies were selected covering 23 biotic and 70 abiotic experiments. SRA study ID, stress type, genotype, known properties of the genotype, treatment, plant tissue, Pubmed ID, number of runs per SRA study, total amount of re-analyzed data in Mbases and Mbytes are given. NA= non-available, DAP = days after planting, DAG = days after germination, DAS = days after sowing.

SRA Study	Stress Type	Genotype	Genotype Known Properties	Treatment	Tissue(s)	Pubmed ID	#RUNS	Mbases	Mbytes
SRP009826	ABIOTIC	Be Sweet 292	Lots of pods containing large seeds	Ozone (151.2 nL L^{-1})	Leaf 45 DAP	26324463	6	16106	9933
SRP024277	ABIOTIC	Pioneer 93B15	Commercial Cultivar	Ozone (100 nl L^{-1}), Temperature (infrared), Drought (interception of nighttime rainfall)	Seed Coat (mature R8)	29233093	25	69846	50469
SRP031889	ABIOTIC	Clark	Iron Efficient	Fe deficiency (100 µM vs. 50 µM)	Leaf, Root (16 DAG)	25149281	16	15871	9489
SRP035871	ABIOTIC	Pioneer 93B15	Commercial Cultivar	Ozone (150 ppb)	Flower, Pod (R2)	25430603	16	66177	44224
SRP041622	ABIOTIC	Williams 82	Reference Genome	Dehydratation (air exposure), Salt (100 mM)	Root (V1)	25362847	21	11379	7746
SRP045932	ABIOTIC	Benning, PI416937	Benning= Drought Sensitive, PI416937=Drought Tolerant	Water Deficiency (air exposure)	Shoot (R2 stage of flowering)	25644024	24	23309	10578
SRP050050	ABIOTIC	Williams 82	Reference Genome	Ethylene (25 µl/l ethylene for 0, 12h, 24h, 48h, 72 h)	Leaf Abscission Zone, Leaf Petiole (21 DAS)	26697054	30	5693	3644

SRP076153	ABIOTIC	Williams 82	Reference Genome	Drought (withdraw water 7 days), flood (4 cm of water above soil for 7 days)	Leaf (V4 + 7 days of treatment)	27486466	9	23970	16307
SRP058975	ABIOTIC	BR16	Drought sensitive	No more irrigation after V1 + 14 days for 3 days (30% field capacity)	Leaf (V1 + 17 days 6/24h timepoints)	26149272	36	119248	83203
SRP064384	ABIOTIC	Embrapa48	Drought resistant	CO2 (Ambient 400 ppm, Elevated 800 ppm), Drought - Air (50 min)	Root (V3/V4)	30824006	8	40902	27092
SRP105922-SRP105965	ABIOTIC	Williams 82	Reference genome	Drought (NA), high CO2 (800ppm), pH, Temperature (High=35 °C, Low=15°C), Depletion (Fe, K, N, P), Salinity (60mM NaCl)	Root Hair Cells	NA	44	274603	121206
SRP132150	ABIOTIC	C08	Drought sensitive	Salt (150 mM) (0h, 1h, 2h, 4 h, 24h and 48h)	Leaf, Root	29508916	36	121459	83241
SRP108540	ABIOTIC	U06-105454, U06-625083	IDC tolerant line U06-454 and IDC sensitive line UO6-625083	iron deficiency (1µM vs. 25 µM 7+6 days) , alkaline treatment (0, 2.5 or 5 mM $NaH_2CO_{3\ \text{additional 7+4 days}}$)	Root	29403520	36	40556	29531
SRP155375	BIOTIC	Hikmok	Nematode resistant, ability to absorb Si and lack of *Rps* genes	0, 4, 7 and 14 days inoculation with *P. soja*	Root (14-28 DAG)	29848307	40	84426	34017
SRP056137	BIOTIC	Forrest	Resistant cultivar	pathogenic strain FO40 and non-pathogenic strain FO36 (72h, 96h post infection)	Root	26689712	18	73671	49396

SRP091708	BIOTIC	Hutcheson	Nematode susceptible	3,6,9 and 12 days inoculation (DAI) after 18 days growth	Root	28945515	24	59028	35701
SRP126743	BIOTIC	Jack	Virus susceptible	14 day old leaflets 0, 12 and 24h after inoculation	Leaf	30535849	6	215537	90646
SRP135932	BIOTIC	Huipizhi Heidou	Nematode resistant	Soybean seedlings 5, 10 and 15 days post infection	Root	29169106	12	84759	42387

Table S3. Gene model list used in our re-analysis of 23 biotic and 70 abiotic stress experiments. Gene model ID (Wm82 a2.v1), enzymatic activity, symbol as used in this paper and the best *Arabidopsis thaliana* TAIR10 pBlast hit are given.

Gene Model a2.v1	Enzymatic activity	Abbreviation	Best Arabidopsis TAIR10 hit
Glyma.16G049300	aspartate kinase	*Gm.AK*	AT5G13280.1 (aspartate kinase 1)
Glyma.16G147300	aspartate kinase	*Gm.AK*	AT5G13280.1 (aspartate kinase 1)
Glyma.02G066100	aspartate kinase	*Gm.AK*	AT5G13280.1 (aspartate kinase 1)
Glyma.19G102100	aspartate kinase	*Gm.AK*	AT5G13280.1 (aspartate kinase 1)
Glyma.08G107800	aspartate kinase/homoserine dehydrogenase	*Gm.AK/HSDH*	AT4G19710.2 (aspartate kinase-homoserine dehydrogenase ii)
Glyma.05g151100	aspartate kinase/homoserine dehydrogenase	*Gm.AK/HSDH*	AT4G19710.2 (aspartate kinase-homoserine dehydrogenase ii)
Glyma.05G240700	aspartate-semialdehyde dehydrogenase	*Gm.ASADH*	AT1G14810.1 (semialdehyde dehydrogenase family protein)
Glyma.08G047800	aspartate-semialdehyde dehydrogenase	*Gm.ASADH*	AT1G14810.1 (semialdehyde dehydrogenase family protein)
Glyma.09G268200	4-hydroxy-tetrahydrodipicolinate synthase	*Gm.DHDPS-A1*	T2G45440.1 (dihydrodipicolinate synthase)
Glyma.18G221700	4-hydroxy-tetrahydrodipicolinate synthase	*Gm.DHDPS-A2*	AT2G45440.1 (dihydrodipicolinate synthase)
Glyma.03G022300	4-hydroxy-tetrahydrodipicolinate synthase	*Gm.DHDPS-B*	AT2G45440.1 (dihydrodipicolinate synthase)
Glyma.16G028900	4-hydroxy-tetrahydrodipicolinate reductase	*Gm.DHDPR*	AT3G59890.1 (Dihydrodipicolinate reductase, bacterial/plant)
Glyma.07G060300	4-hydroxy-tetrahydrodipicolinate reductase	*Gm.DHDPR*	AT3G59890.1 (Dihydrodipicolinate reductase, bacterial/plant)
Glyma.08G063500	L,L-diaminopimelate aminotransferase	*Gm.LL-DAP-AT*	AT4G33680.1 (Pyridoxal phosphate (PLP)-dependent transferases superfamily protein)
Glyma.07G185700	L,L-diaminopimelate aminotransferase	*Gm.LL-DAP-AT*	AT4G33680.1 (Pyridoxal phosphate (PLP)-dependent transferases superfamily protein)
Glyma.03G173400	diaminopimelate epimerase	*Gm.DAPE*	AT3G53580.1 (diaminopimelate epimerase family protein)
Glyma.19G174300	diaminopimelate epimerase	*Gm.DAPE*	AT3G53580.1 (diaminopimelate epimerase family protein)
Glyma.13G140700	diaminopimelate decarboxylase	*Gm.DAPDC*	AT3G14390.1 (Pyridoxal-dependent decarboxylase family protein)

Glyma.03G181200	diaminopimelate decarboxylase	*Gm.DAPDC*	AT5G11880.1 (Pyridoxal-dependent decarboxylase family protein)
Glyma.19G182000	diaminopimelate decarboxylase	*Gm.DAPDC*	AT5G11880.1 (Pyridoxal-dependent decarboxylase family protein)
Glyma.10G053600	diaminopimelate decarboxylase	*Gm.DAPDC*	AT5G11880.1 (Pyridoxal-dependent decarboxylase family protein)
Glyma.13G115500	lysine-ketoglutarate reductase/ saccharopine dehydrogenase	*Gm.LKR/SDH*	AT4G33150.1 (lysine-ketoglutarate reductase/saccharopine dehydrogenase bifunctional enzyme)
Glyma.17G044300	lysine-ketoglutarate reductase/ saccharopine dehydrogenase	*Gm.LKR/SDH*	AT4G33150.1 (lysine-ketoglutarate reductase/saccharopine dehydrogenase bifunctional enzyme)
Glyma.08G180600	AGD2-like defense response protein 1	*Gm.ALD1*	AT2G13810.1 (AGD2-like defense response protein 1)
Glyma.13G113100	DIMETHYLANILINE MONOOXYGENASE	*Gm.FMO1*	AT1G19250.1 (flavin-dependent monooxygenase 1)
Glyma.17G046600	DIMETHYLANILINE MONOOXYGENASE	*Gm.FMO1*	
Glyma.10G230300	ORNITHINE CYCLODEAMINASE-RELATED	*Gm.SARD4*	AT5G52810.1 (NAD(P)-binding Rossmann-fold superfamily protein)
Glyma.20G163100	Ornithine cyclodeaminase/mu-crystallin family	*Gm.SARD4*	AT5G52810.1 (NAD(P)-binding Rossmann-fold superfamily protein)

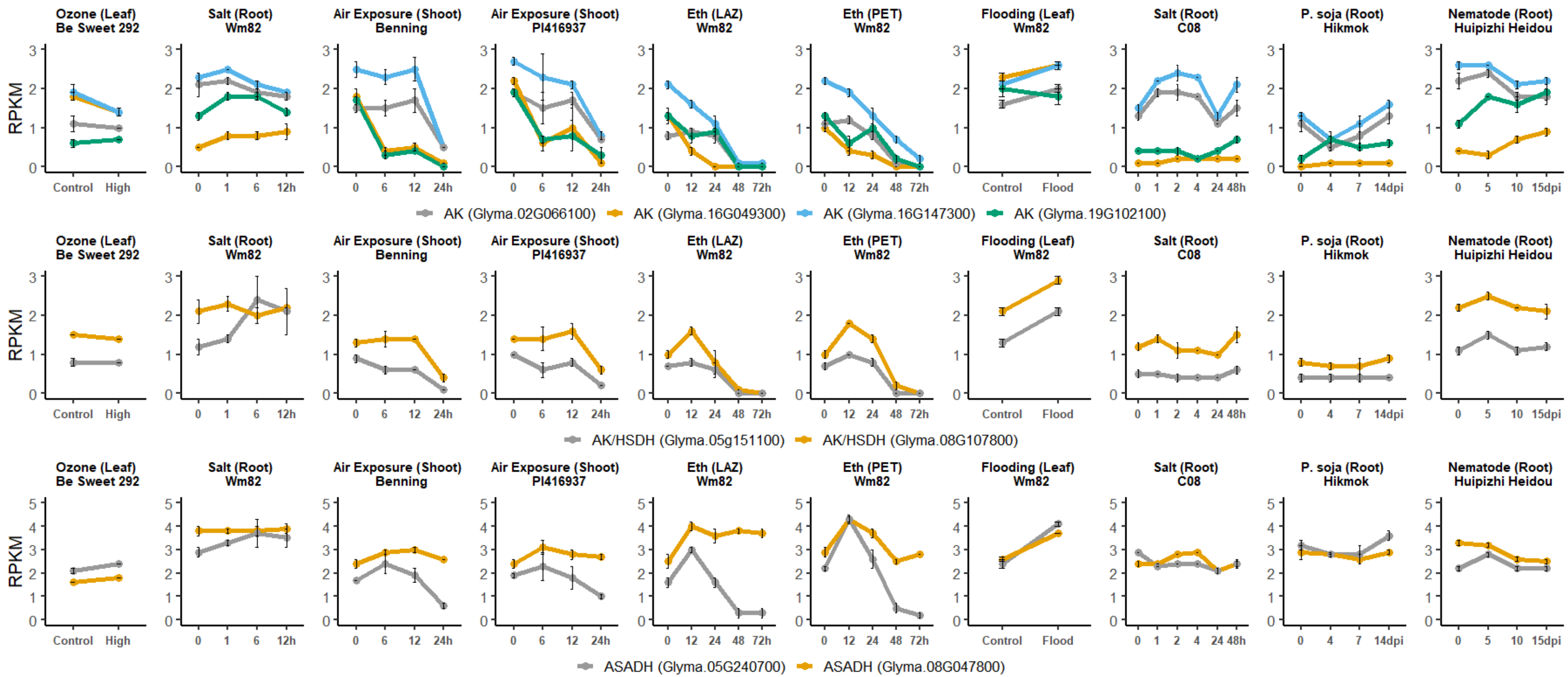

Ozone (Leaf) Be Sweet 292
Salt (Root) Wm82
Air Exposure (Shoot) Benning
Air Exposure (Shoot) PI416937
Eth (LAZ) Wm82
Eth (PET) Wm82
Flooding (Leaf) Wm82
Salt (Root) C08
P. soja (Root) Hikmok
Nematode (Root) Huipizhi Heidou
RPKM
Control High
0 1 6 12h
0 6 12 24h
0 12 24 48 72h
Control Flood
0 1 2 4 24 48h
0 4 7 14dpi
0 5 10 15dpi
AK (Glyma.02G066100)
AK (Glyma.16G049300)
AK (Glyma.16G147300)
AK (Glyma.19G102100)
AK/HSDH (Glyma.05g151100)
AK/HSDH (Glyma.08G107800)
ASADH (Glyma.05G240700)
ASADH (Glyma.08G047800)

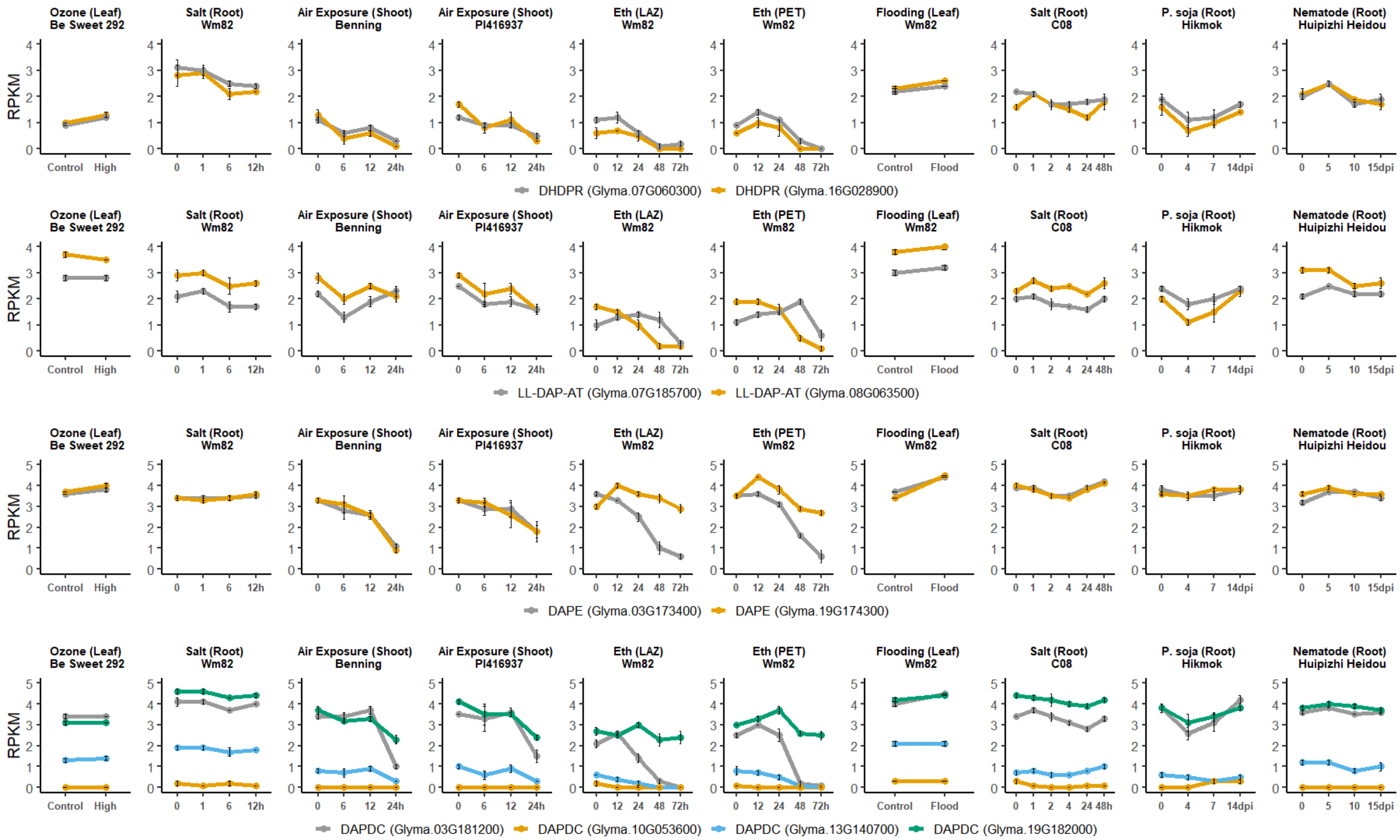

Ozone (Leaf) Be Sweet 292
Salt (Root) Wm82
Air Exposure (Shoot) Benning
Air Exposure (Shoot) PI416937
Eth (LAZ) Wm82
Eth (PET) Wm82
Flooding (Leaf) Wm82
Salt (Root) C08
P. soja (Root) Hikmok
Nematode (Root) Huipizhi Heidou
RPKM
Control
High
Flood
12h
24h
72h
48h
14dpi
15dpi
DHDPR (Glyma.07G060300)
DHDPR (Glyma.16G028900)
LL-DAP-AT (Glyma.07G185700)
LL-DAP-AT (Glyma.08G063500)
DAPE (Glyma.03G173400)
DAPE (Glyma.19G174300)
DAPDC (Glyma.03G181200)
DAPDC (Glyma.10G053600)
DAPDC (Glyma.13G140700)
DAPDC (Glyma.19G182000)

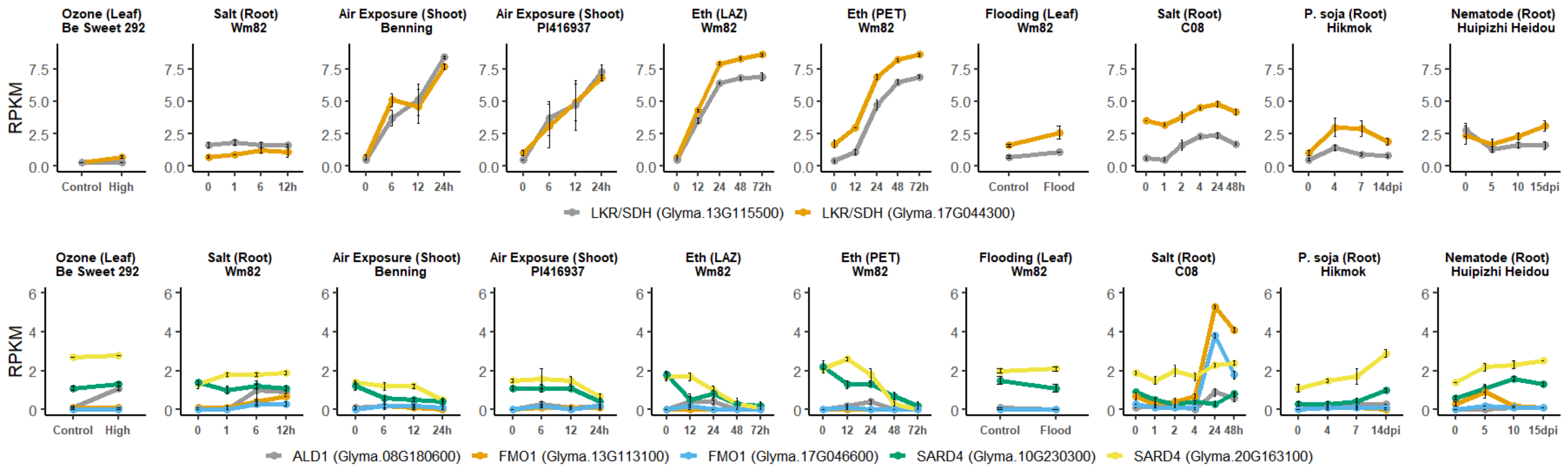

Figure S2. Line graphs of log2 transformed RPKM values for genes involved in lysine biosynthesis (AK, AK/HSDH, ASADH, DHDPR, LL-DAP-AT, DAPE, DAPDC) , lysine catabolism (LKD/SDH) and SAR response (ALD1, SARD4, FMO1) for abiotic or biotic stress experiments in which at least one DHDPS was significantly ($p < 0.05$) differentially expressed. A short summary of the study is given as the graph title with treatment, (plant tissue), genotype. In each Y-axis the log2 RPKM value is given and in each X-axis the treatments in chronological or alphabetical order per SRA study.

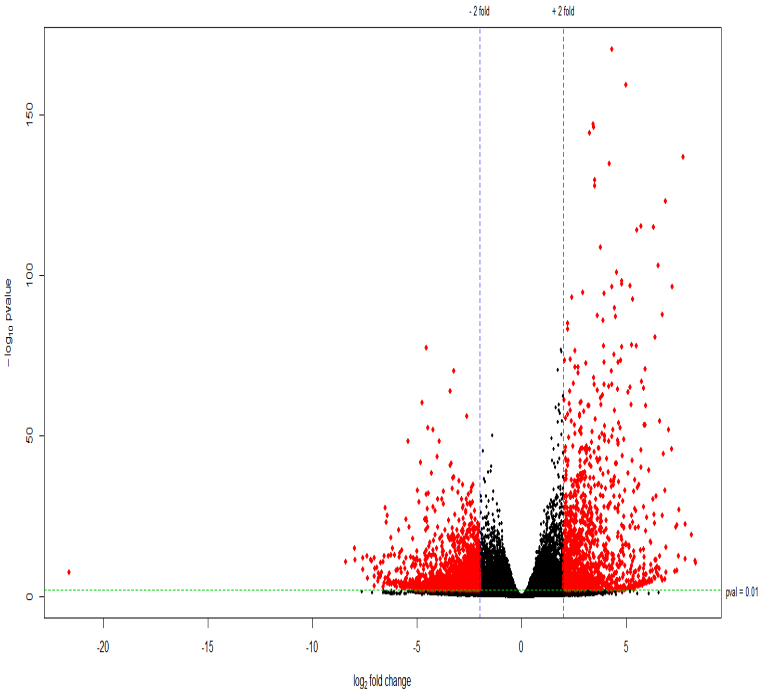

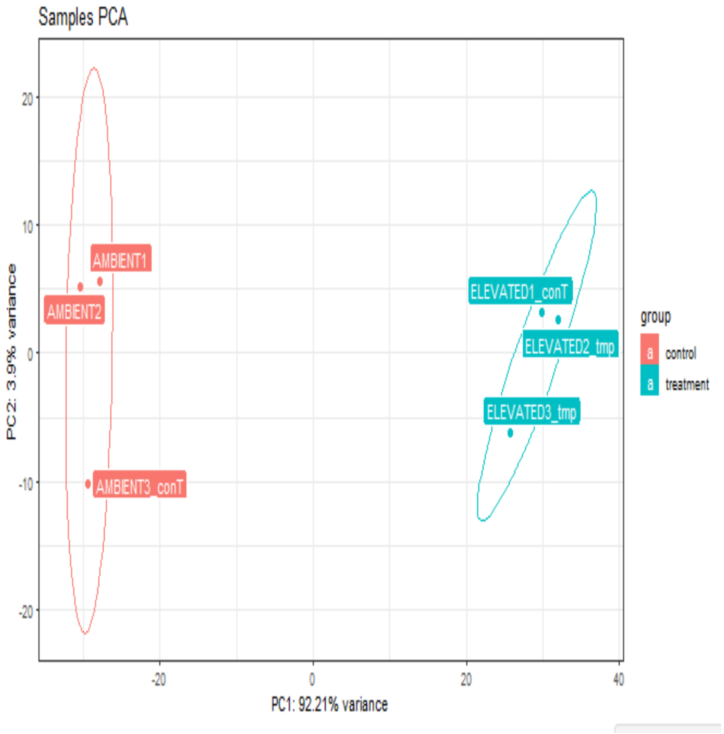

Ab1 - SRP009826 - Ozone (151.2 nL L^{-1}) - Be Sweet 292

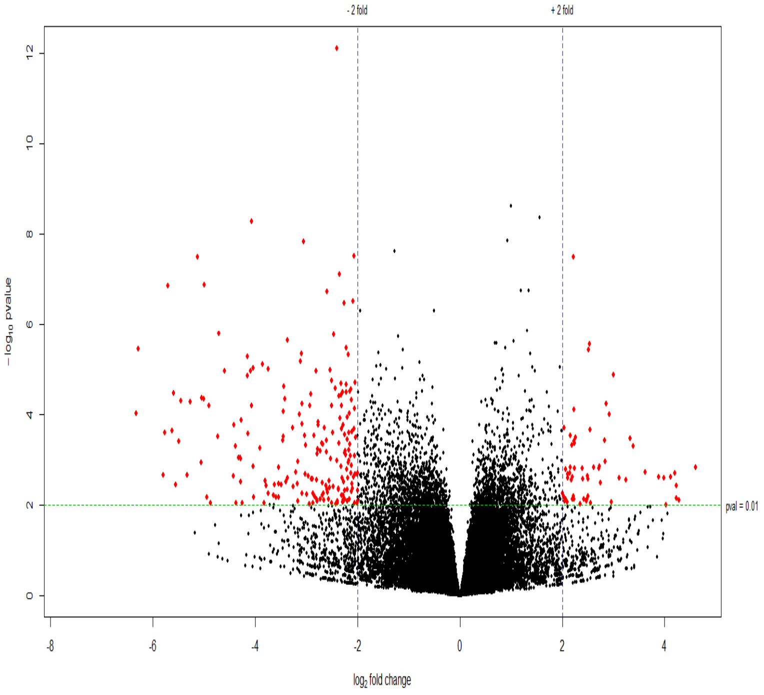

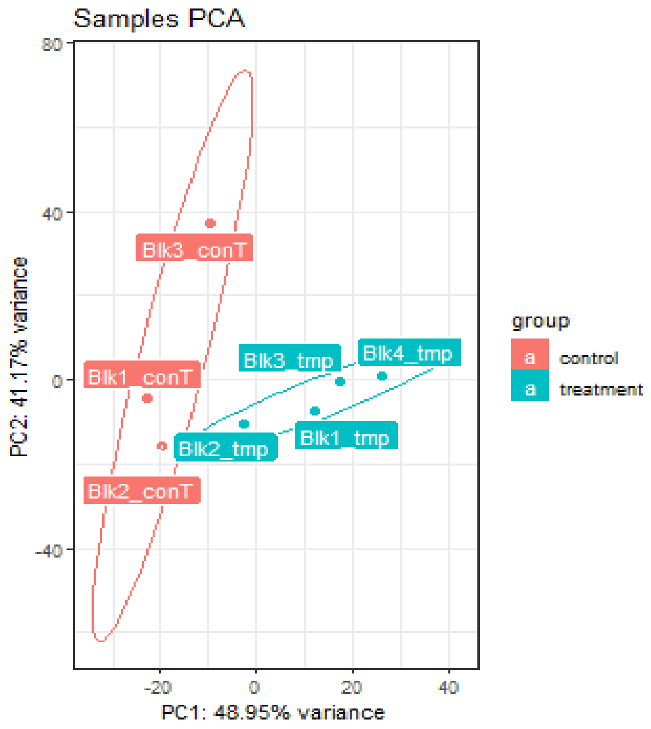

Ab2 - SRP024277 - Temperature (infrared) - Pioneer 93B15 - Seed Coat (mature R8)

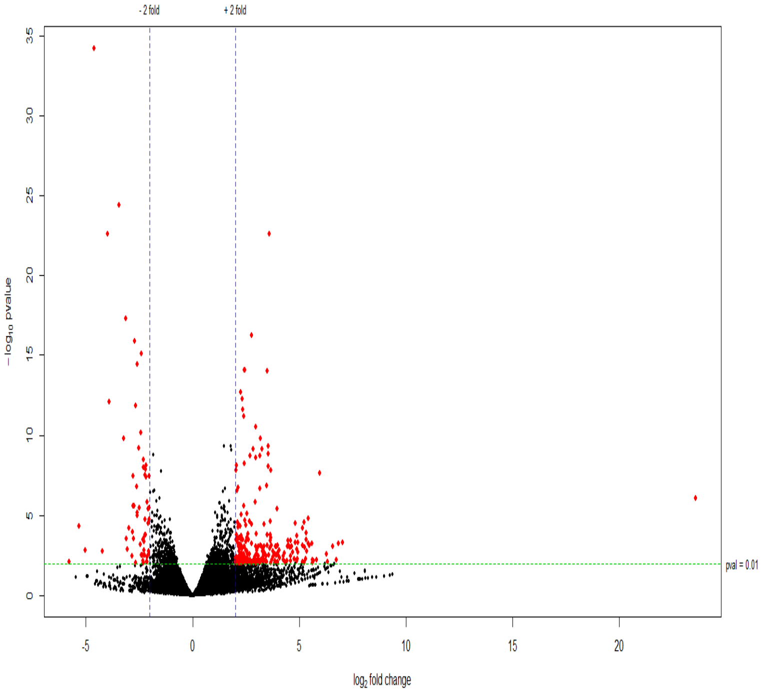

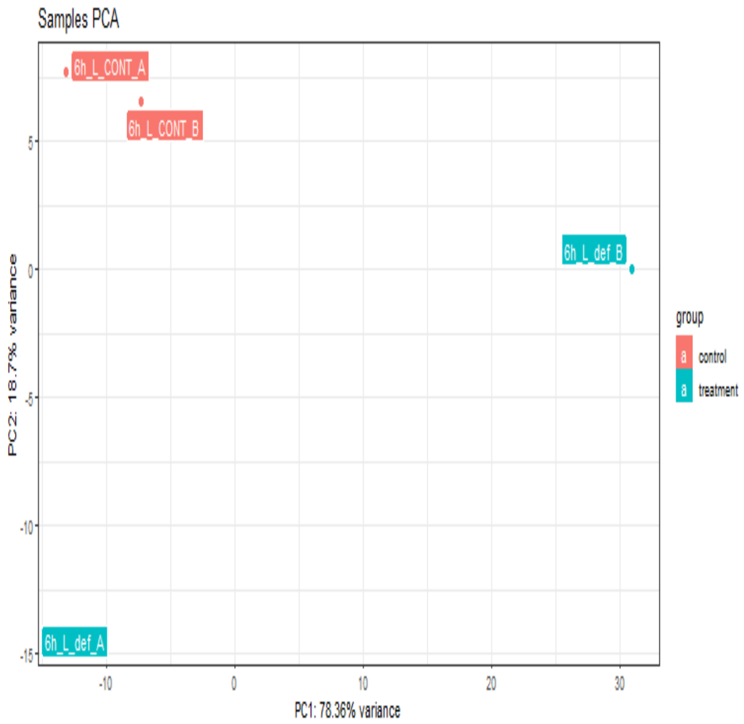

Ab3 - SRP031889 - Fe deficiency (100 µM vs. 50 µM) – Clark – Leaf 6h

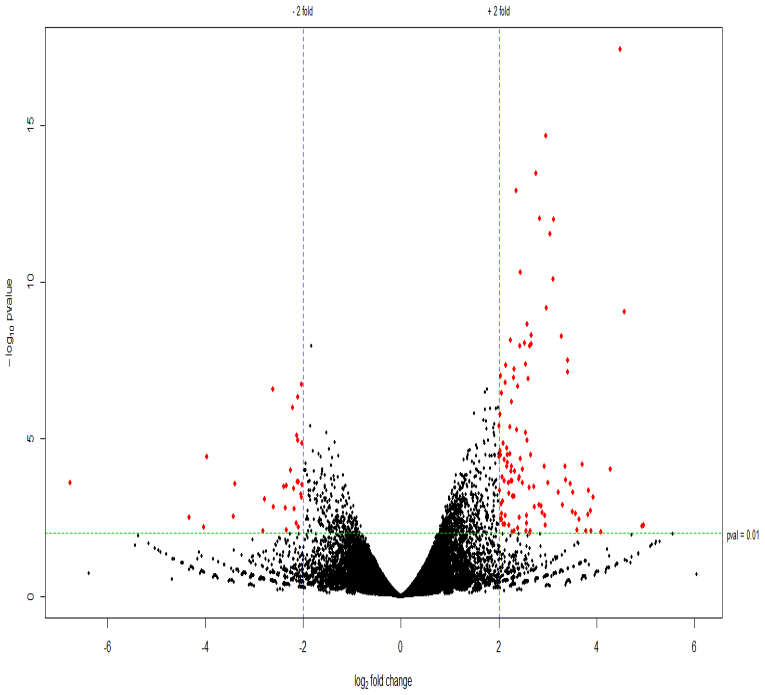

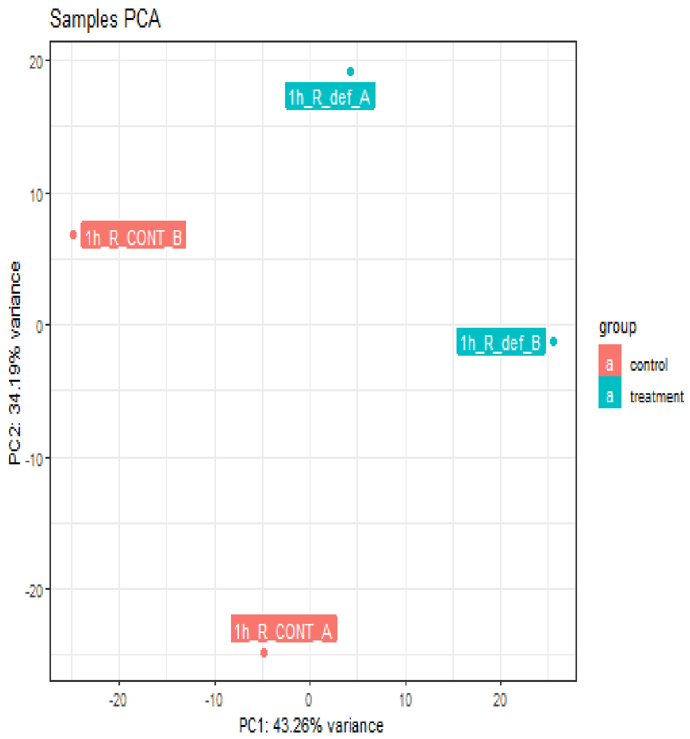

Ab3 - SRP031889 - Fe deficiency (100 µM vs. 50 µM) – Clark – Root 1h

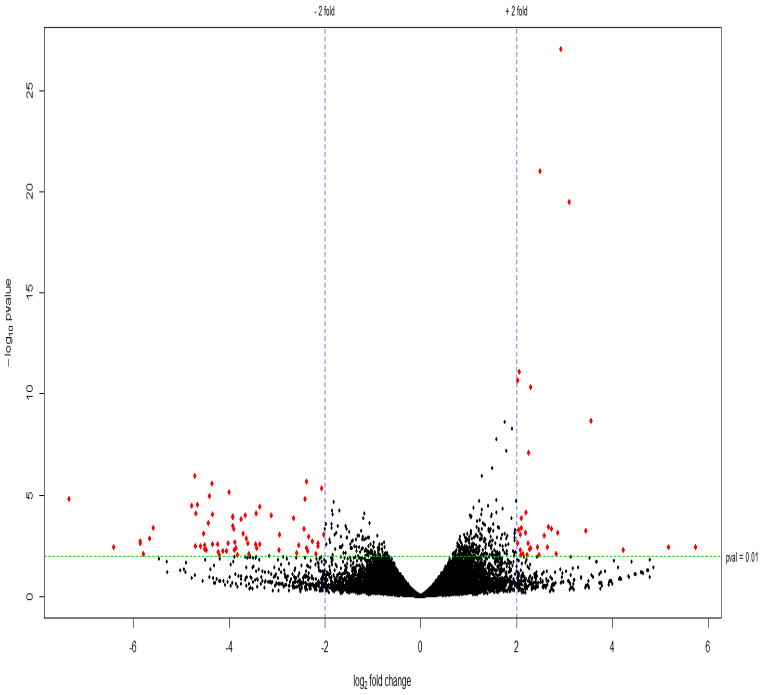

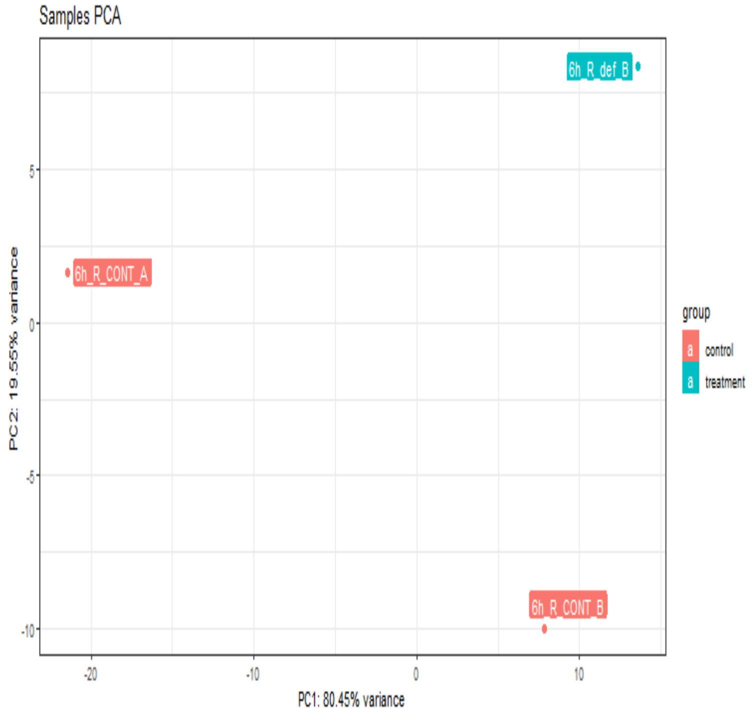

Ab3 - SRP031889 - Fe deficiency (100 µM vs. 50 µM) – Clark – Root 6h

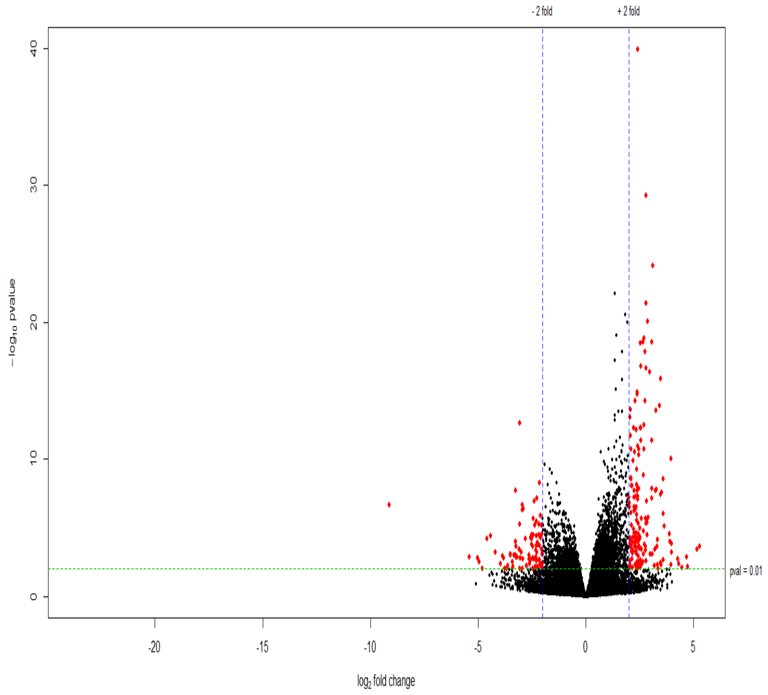

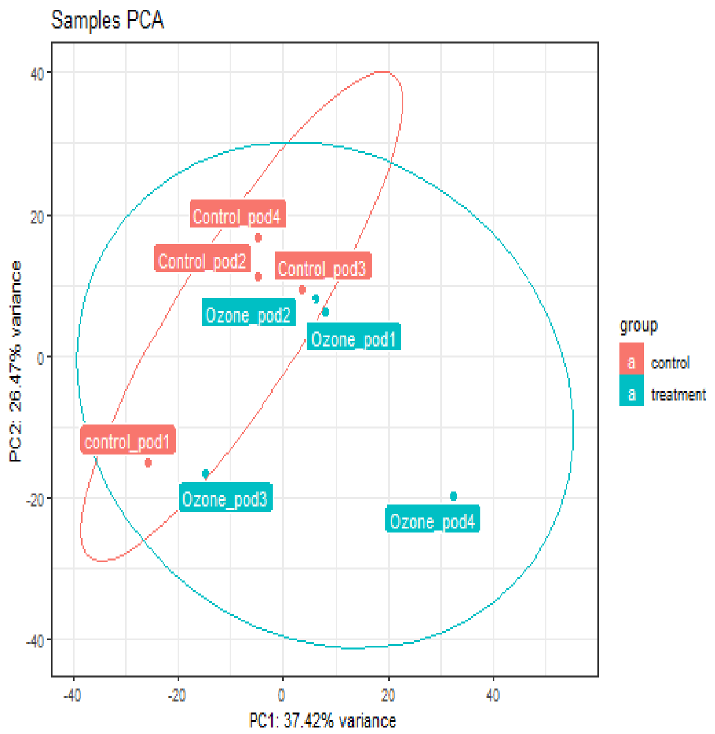

Ab4 - SRP035871_Ozone_Pod

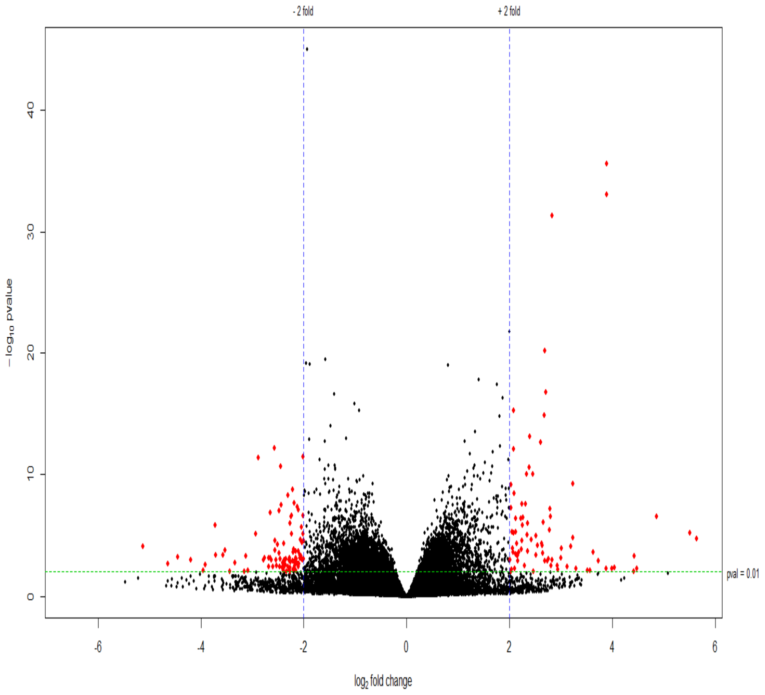

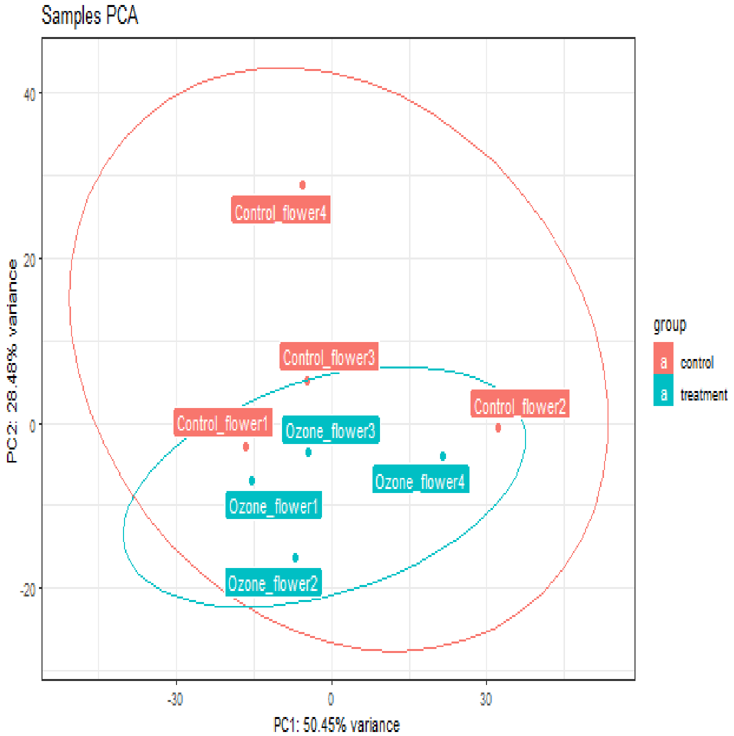

Ab4 - SRP035871_Ozone_Flower

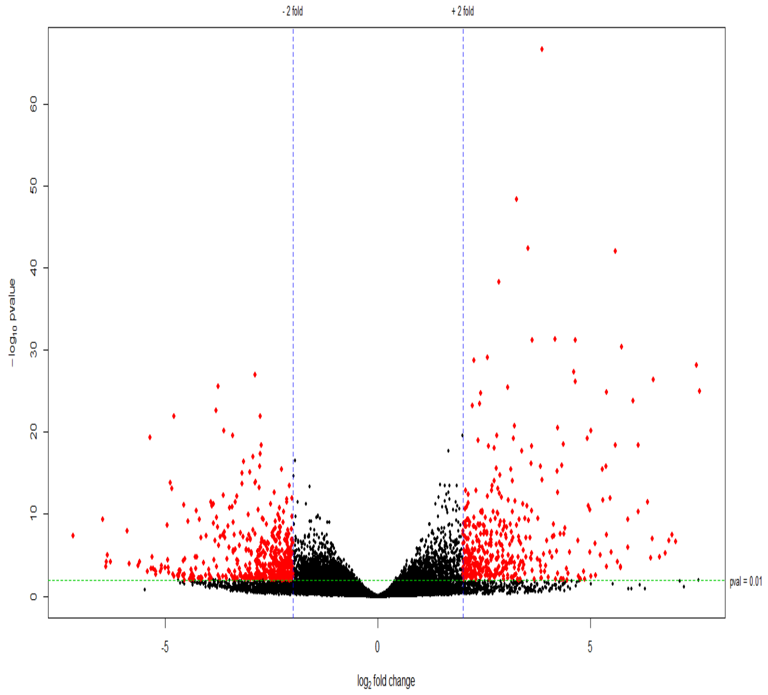

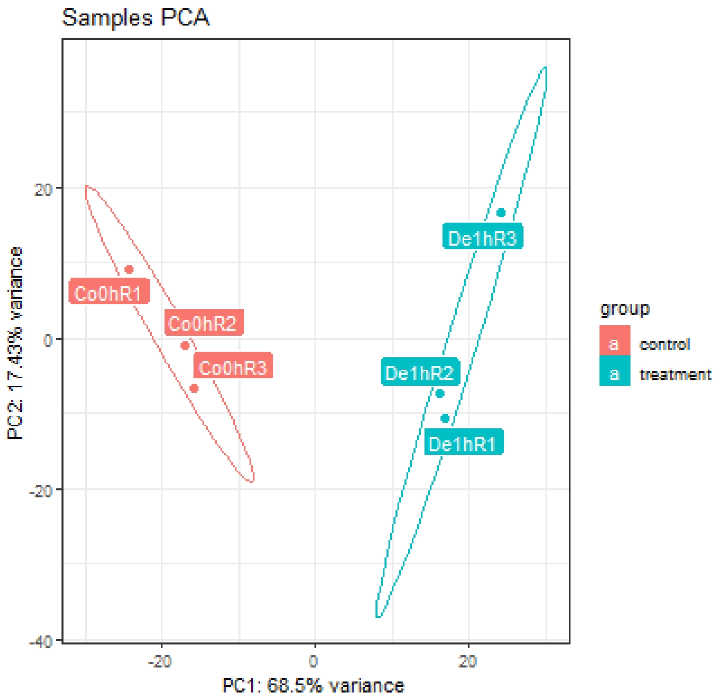

Ab5 - SRP041622_Dehydra 1h - Root (V1)

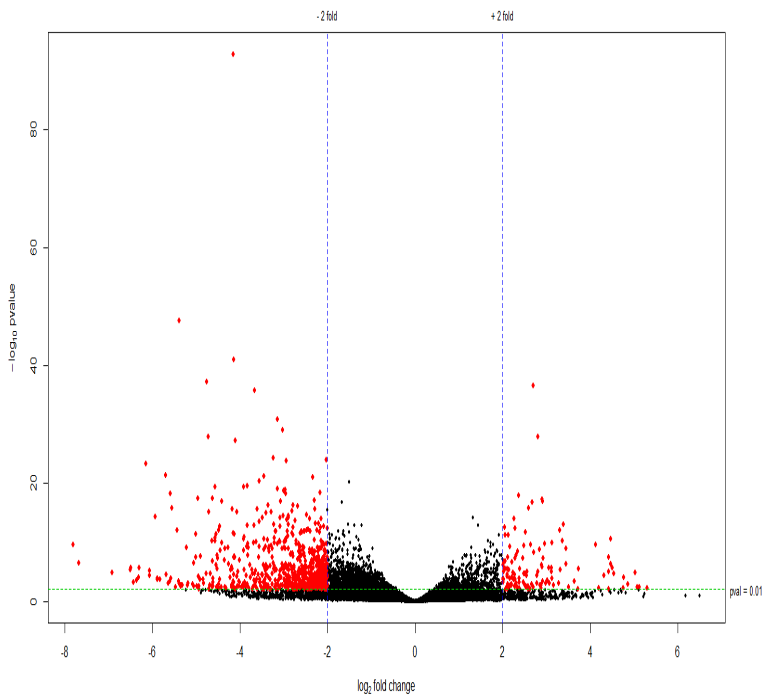

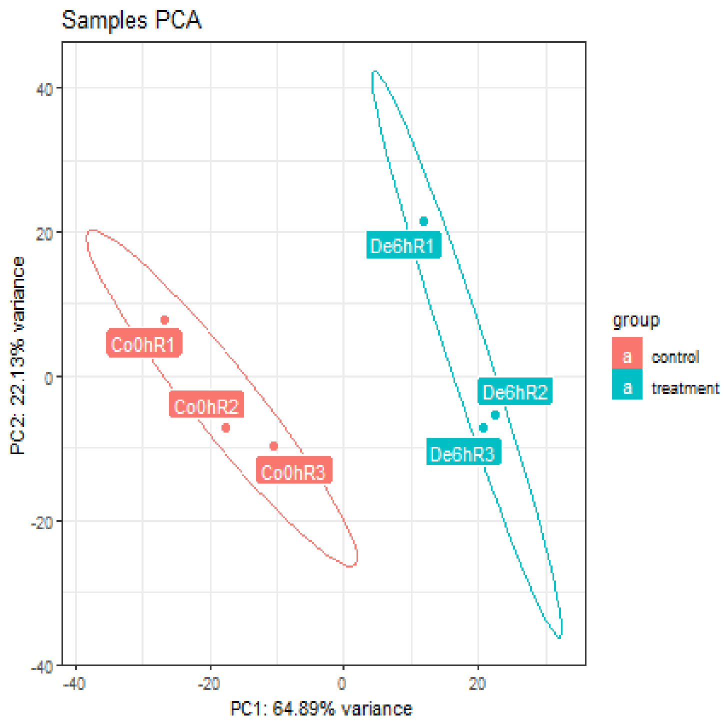

Ab5 - SRP041622_Dehydra 6h - Root (V1)

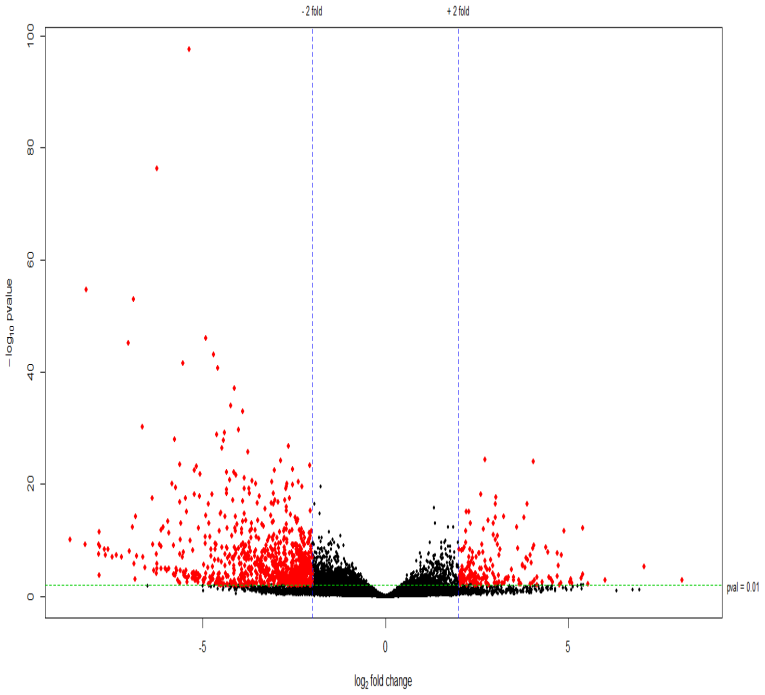

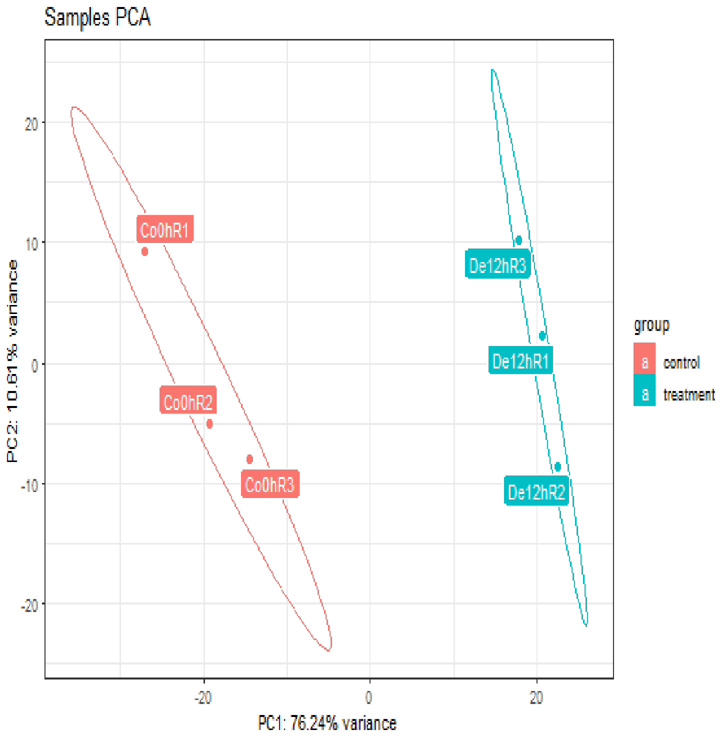

Ab5 - SRP041622_Dehydra 12h - Root (V1)

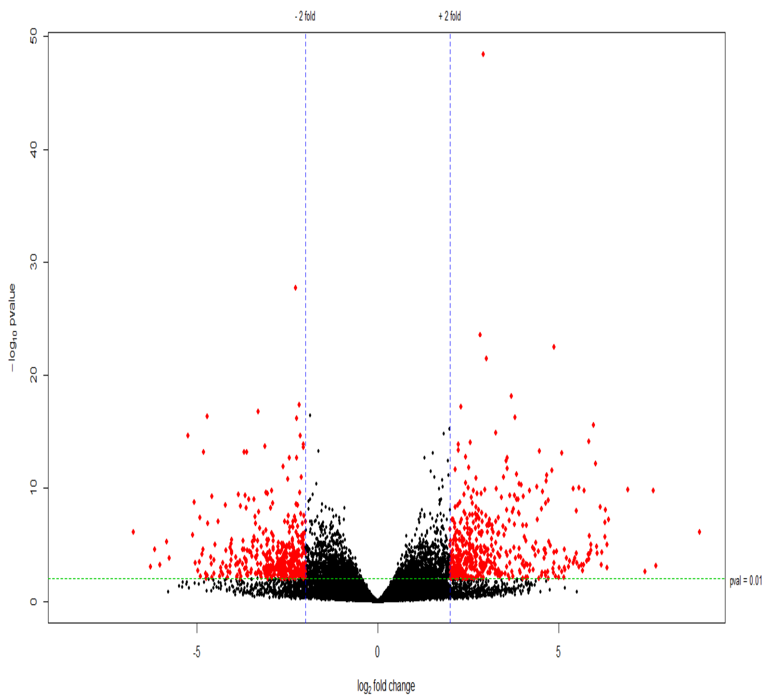

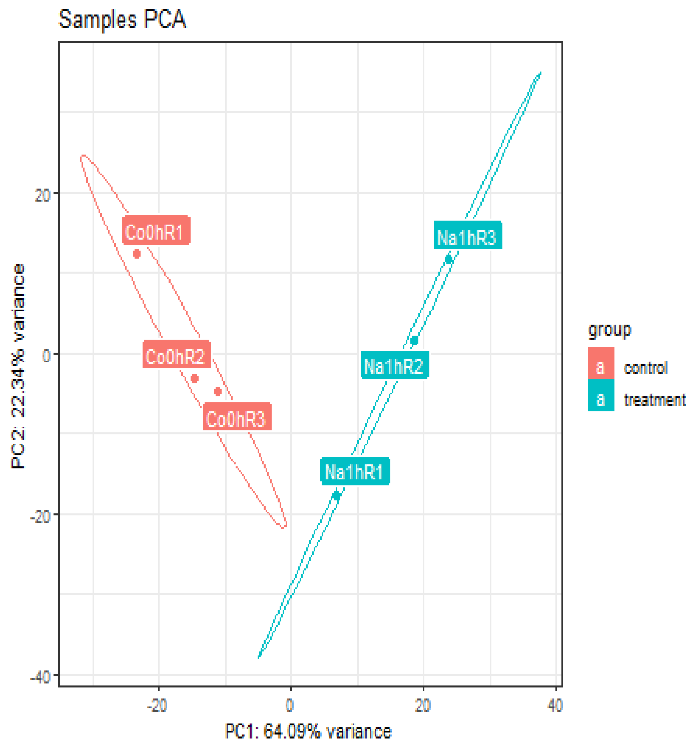

Ab5 - SRP041622_SALT 1h - Root (V1)

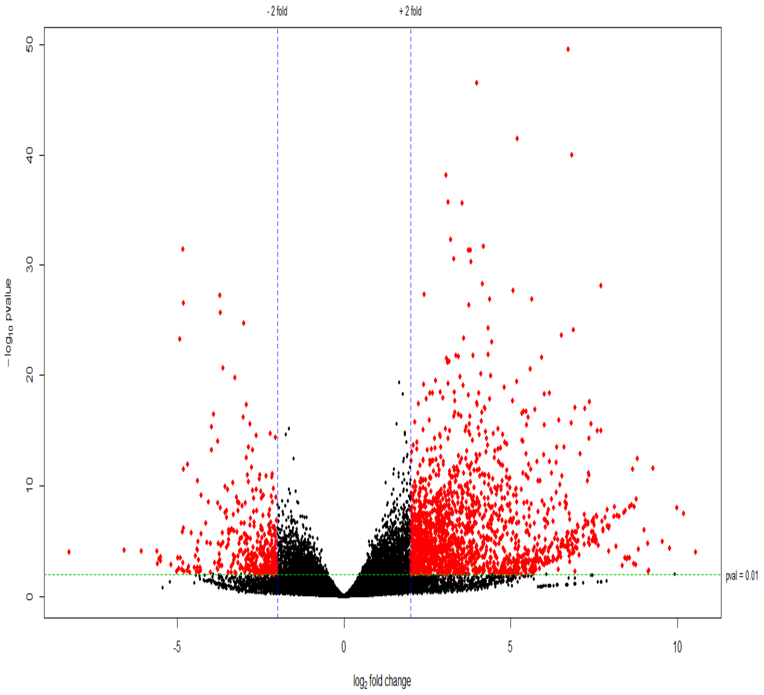

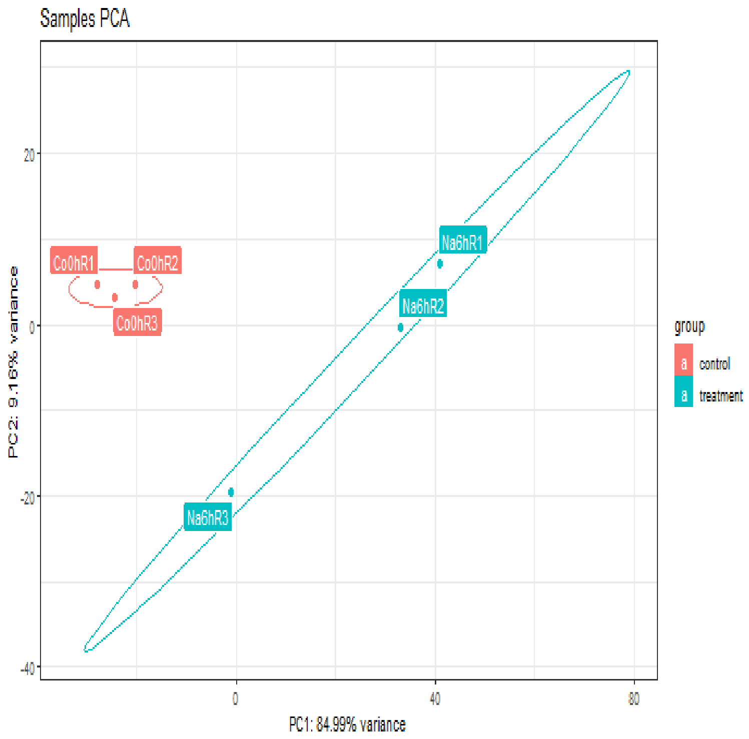

Ab5 - SRP041622_SALT 6h - Root (V1)

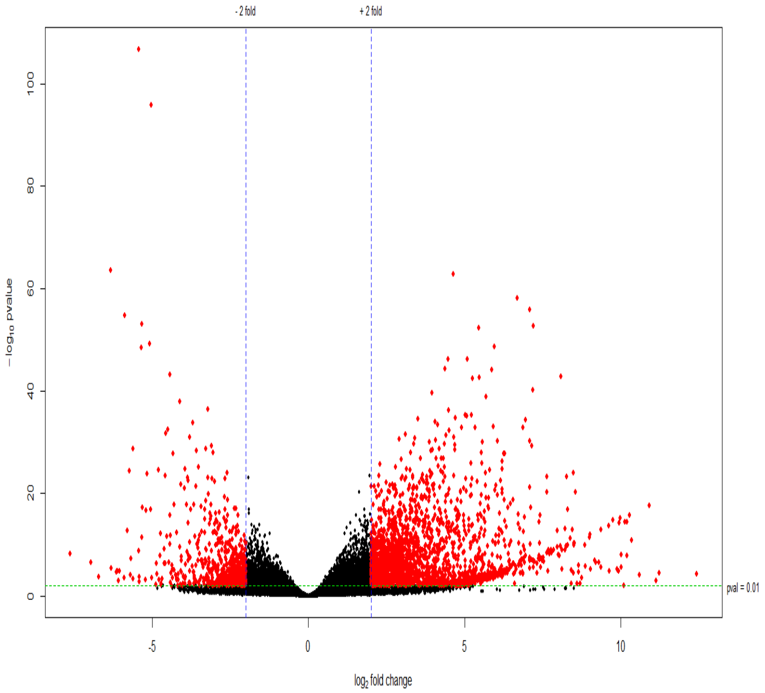

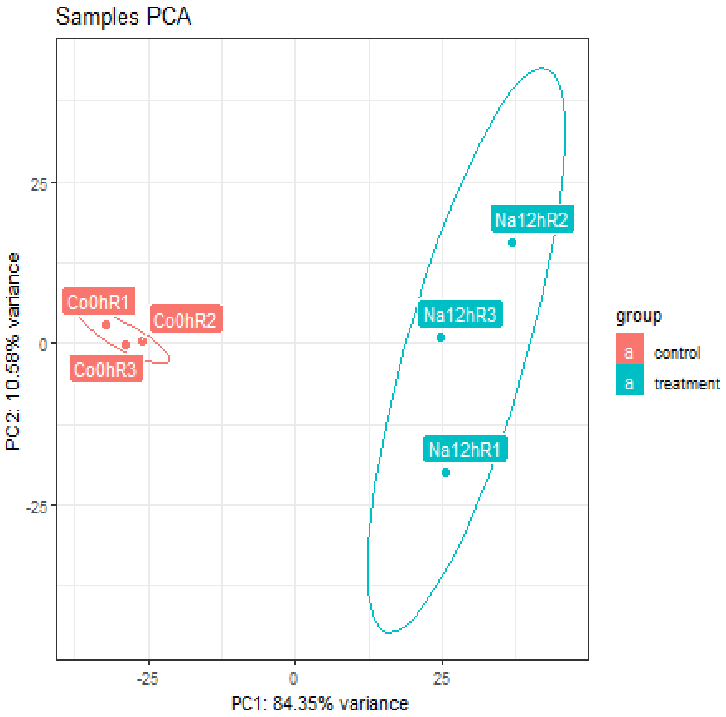

Ab5 - SRP041622_SALT 12h - Root (V1)

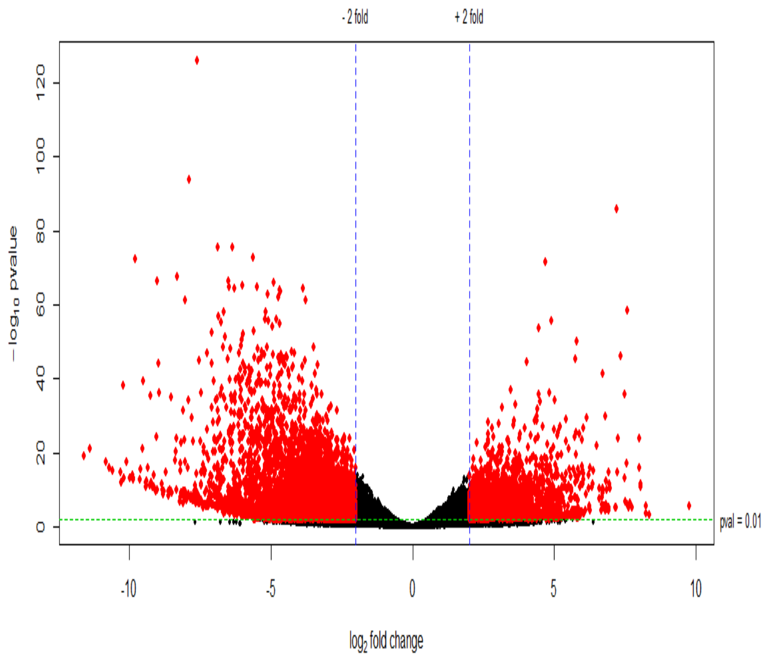

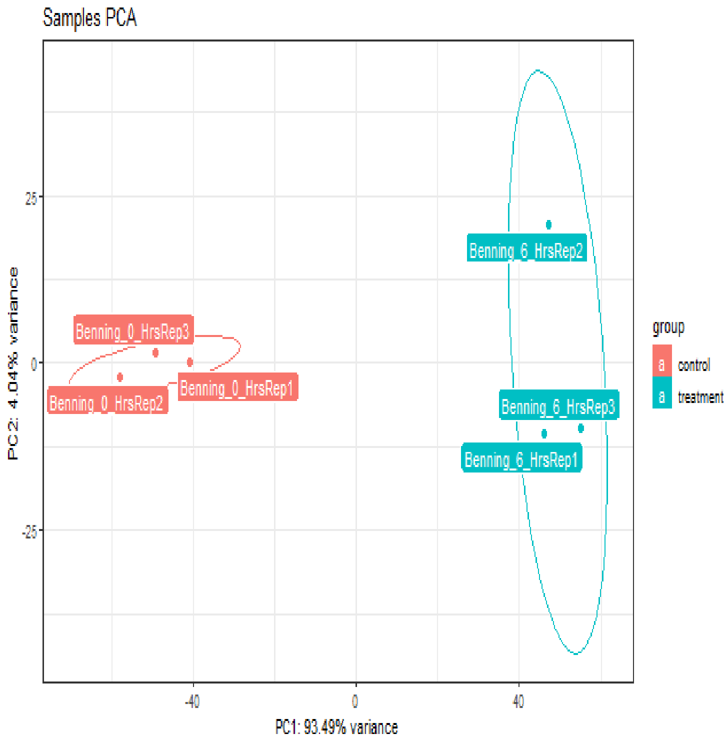

Ab6 - SRP045932_Water_Deficit_Ben6h

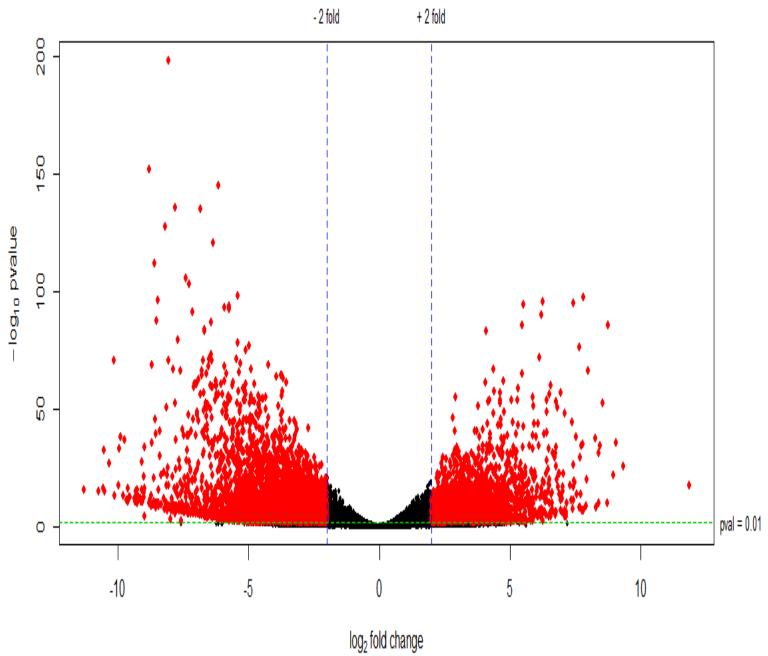

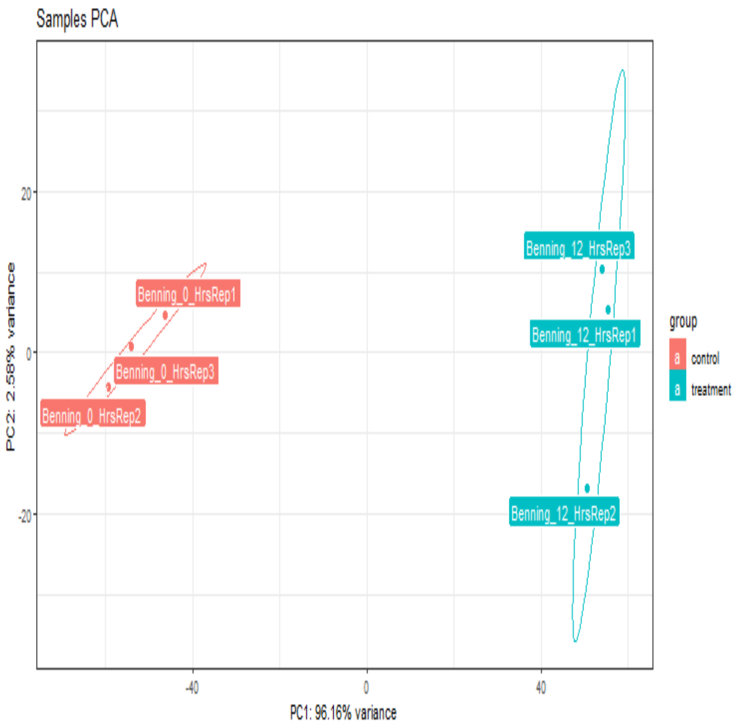

Ab6 -SRP045932_Water_Deficit_Ben12h

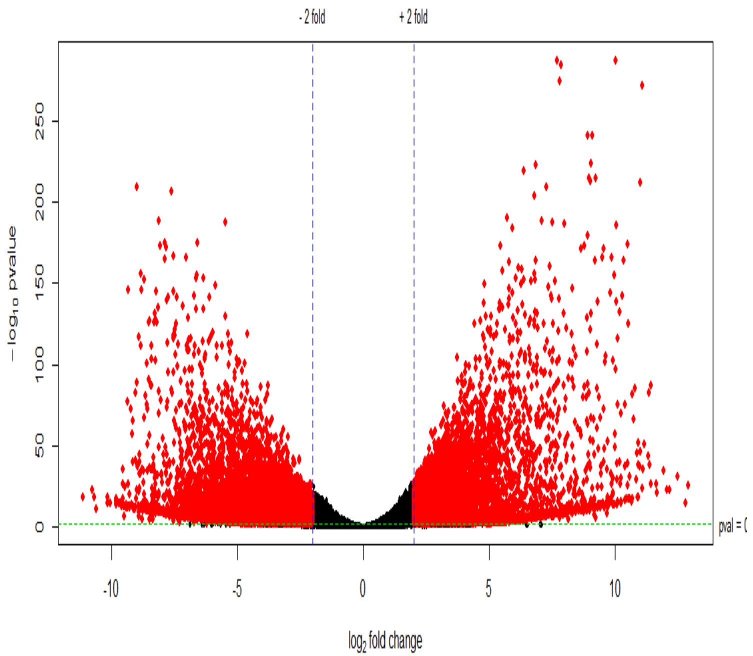

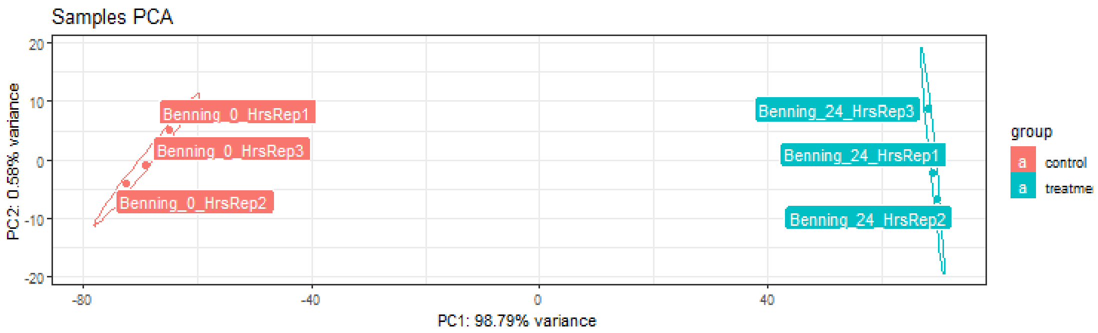

Ab6 -SRP045932_Water_Deficit_Ben24h

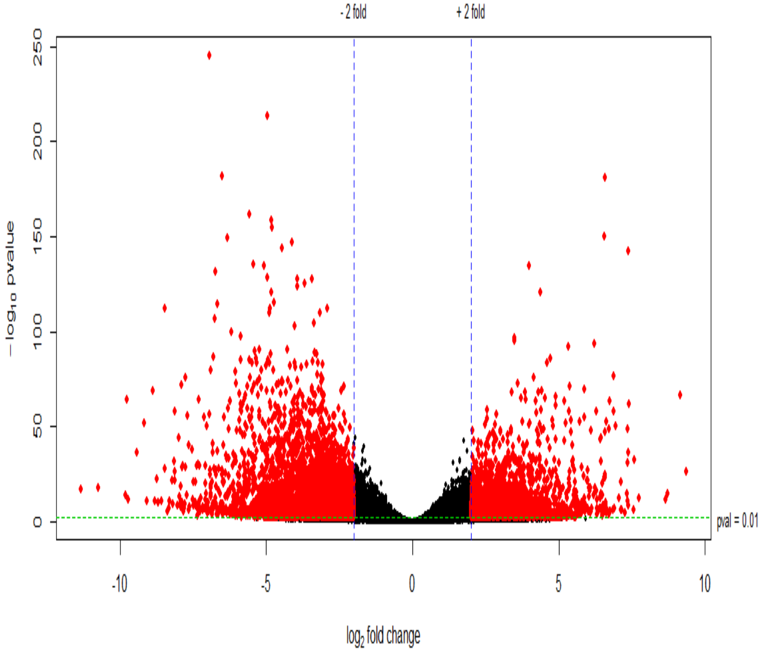

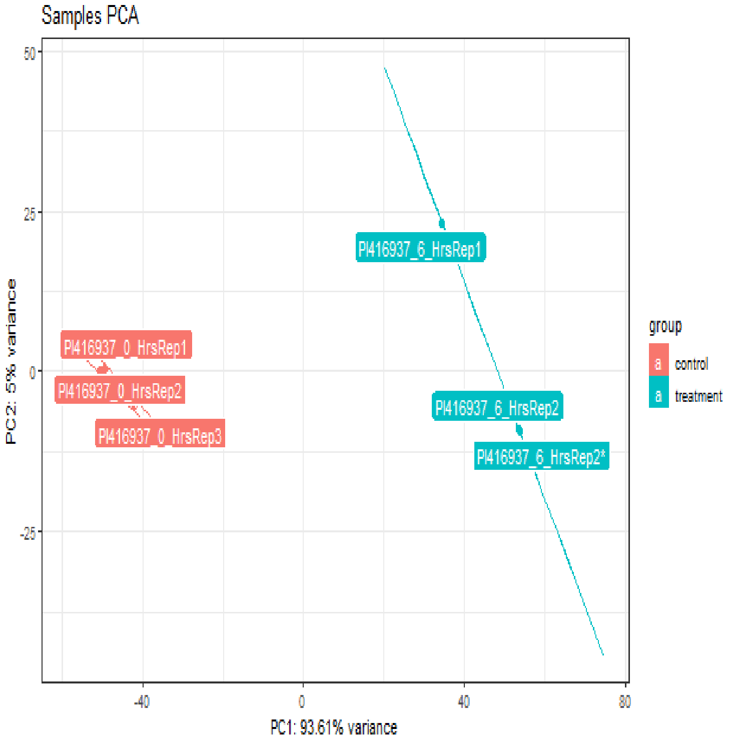

Ab6 - SRP045932_Water_Deficit_PI41_6h

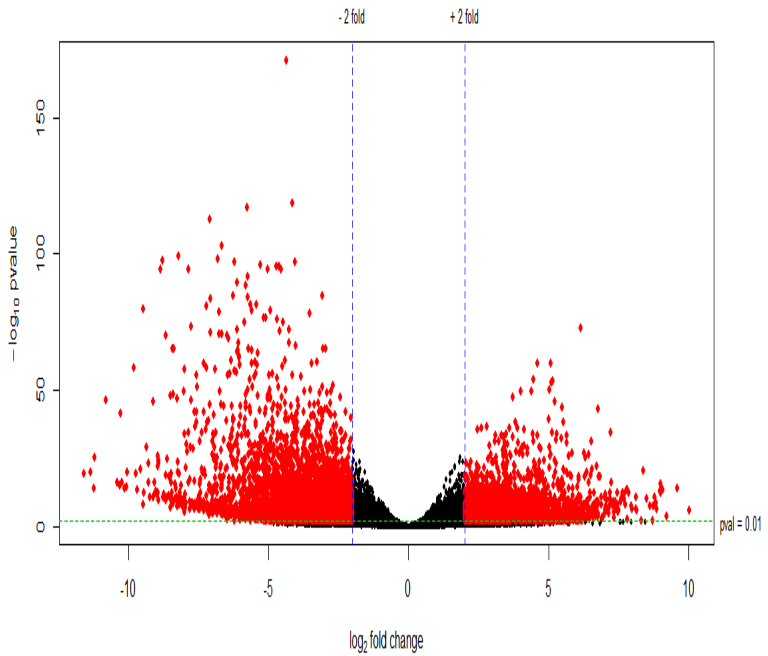

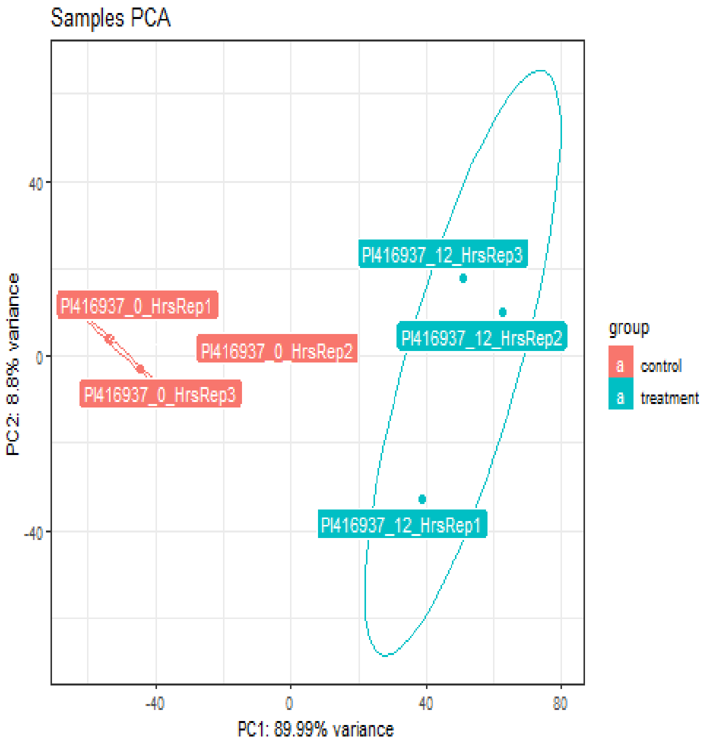

Ab6 - SRP045932_Water_Deficit_PI41_12h

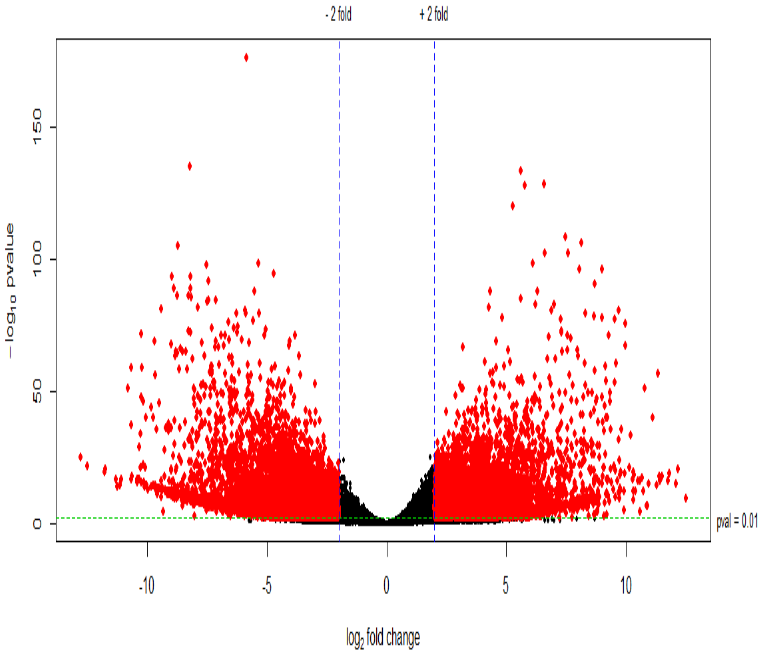

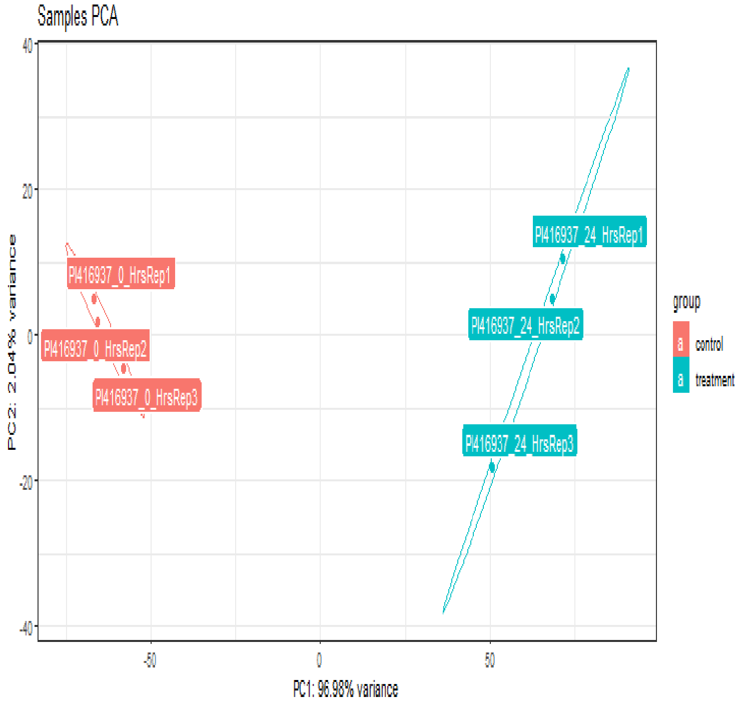

Ab6 - SRP045932_Water_Deficit_PI41_24h

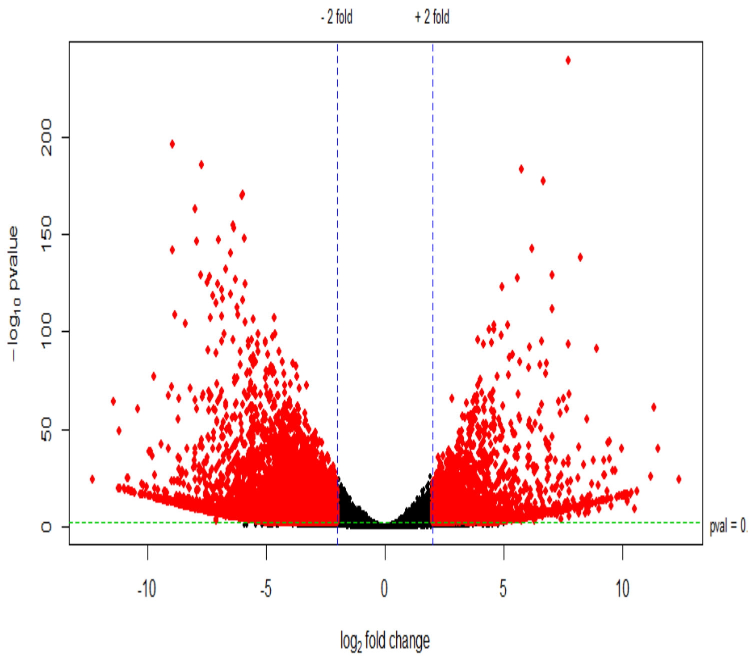

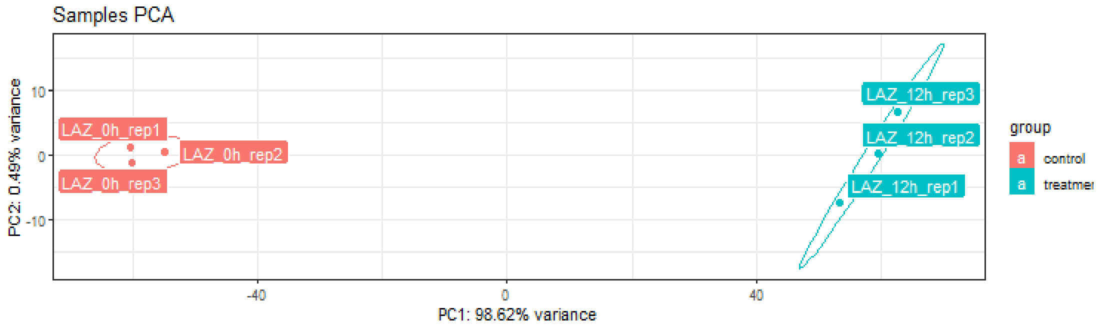

Ab7 - SRP050050_Ethyleen_LAZ12h

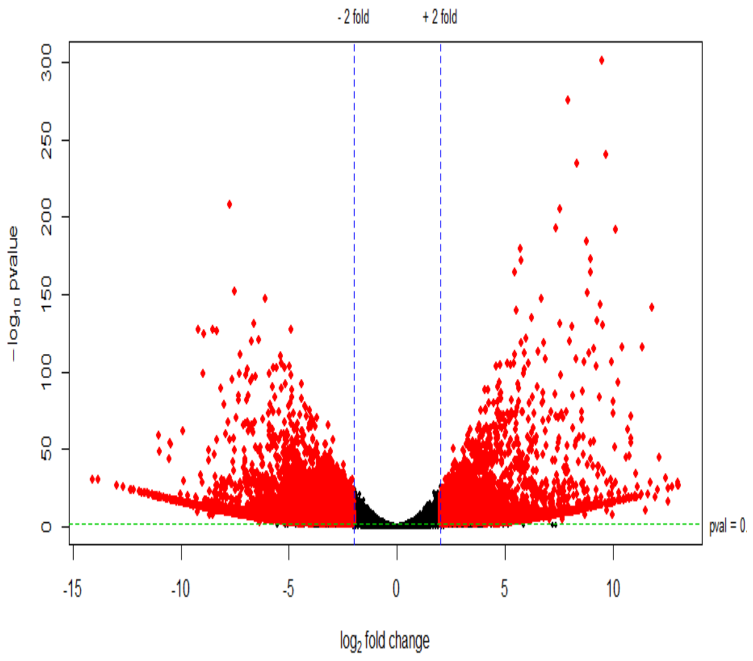

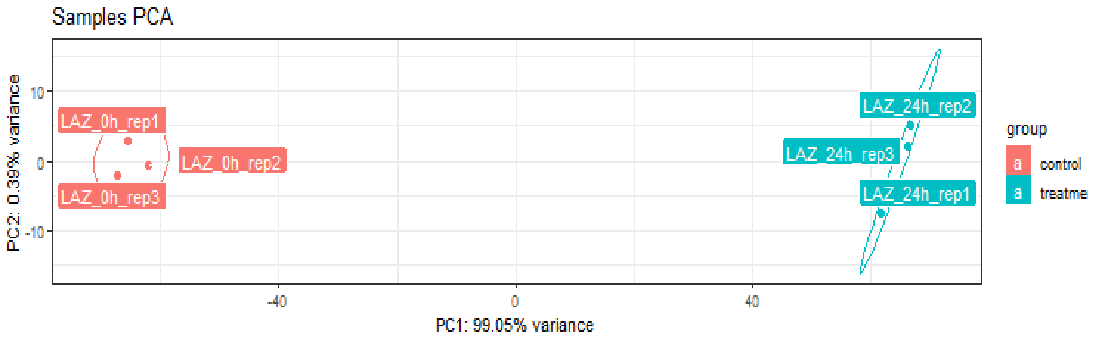

Ab7 - SRP050050_Ethyleen_LAZ24h

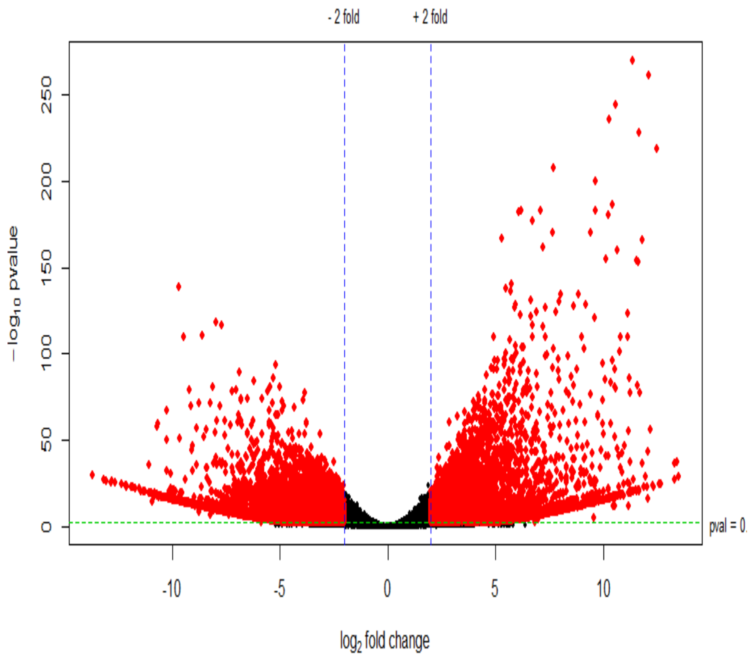

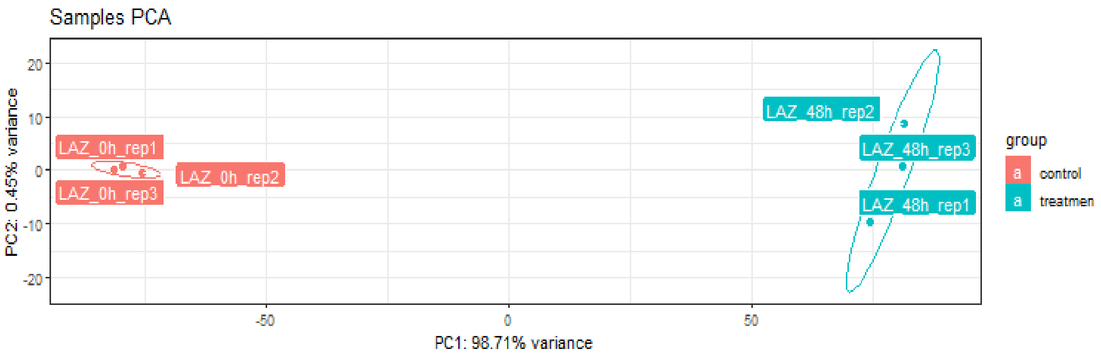

Ab7 - SRP050050_Ethyleen_LAZ48h

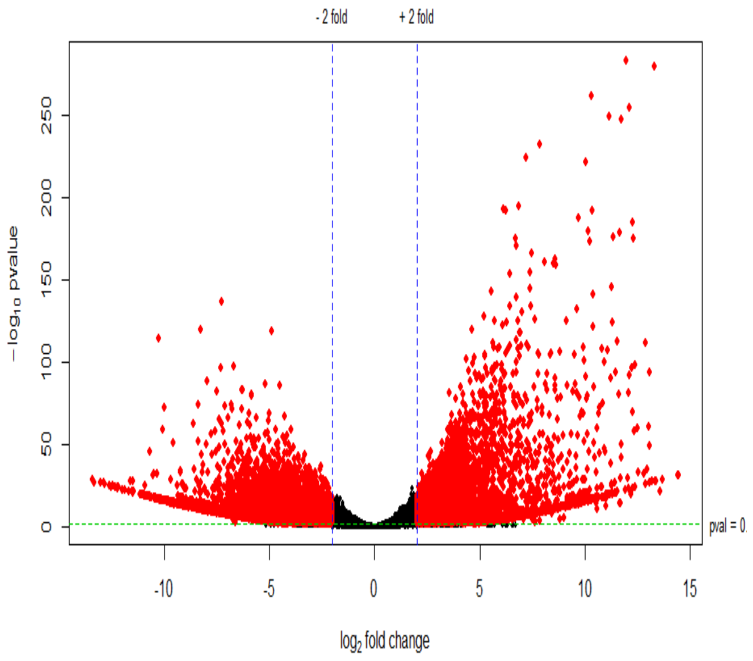

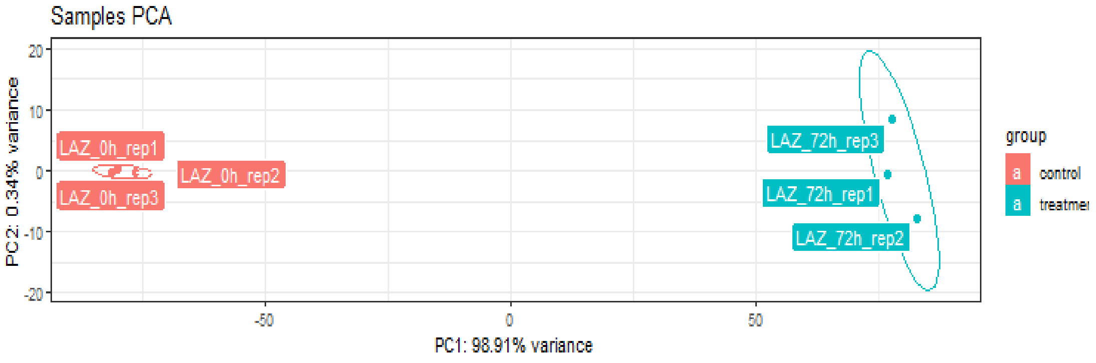

Ab7 - SRP050050_Ethyleen_LAZ72h

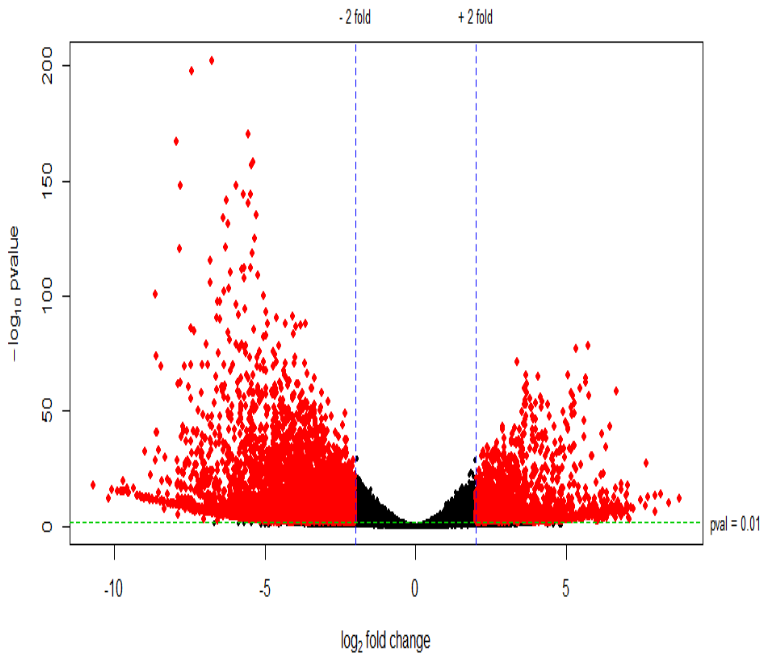

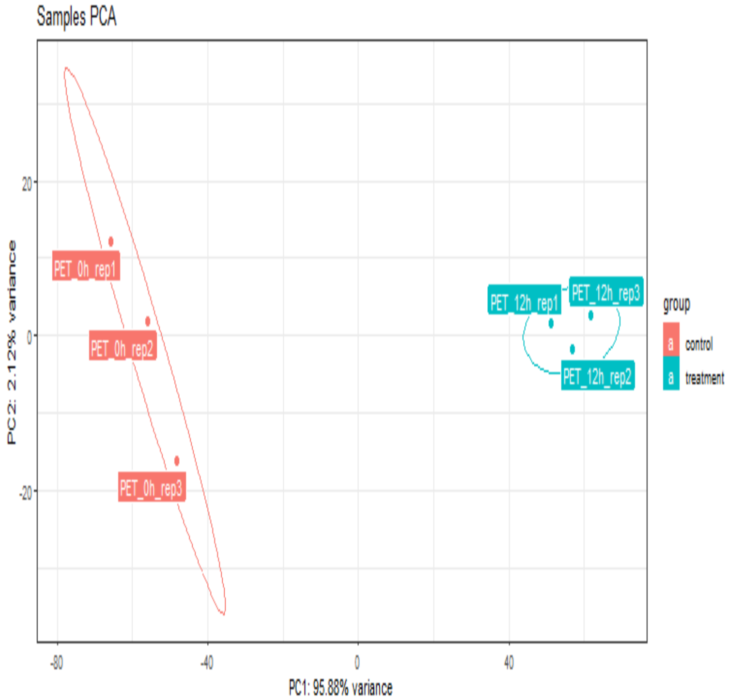

Ab7 - SRP050050_Ethyleen_PET12h

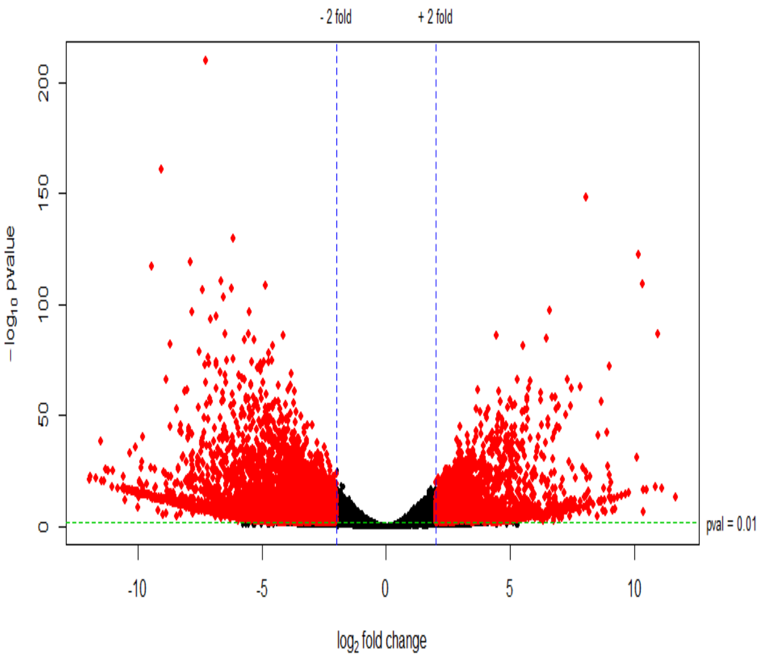

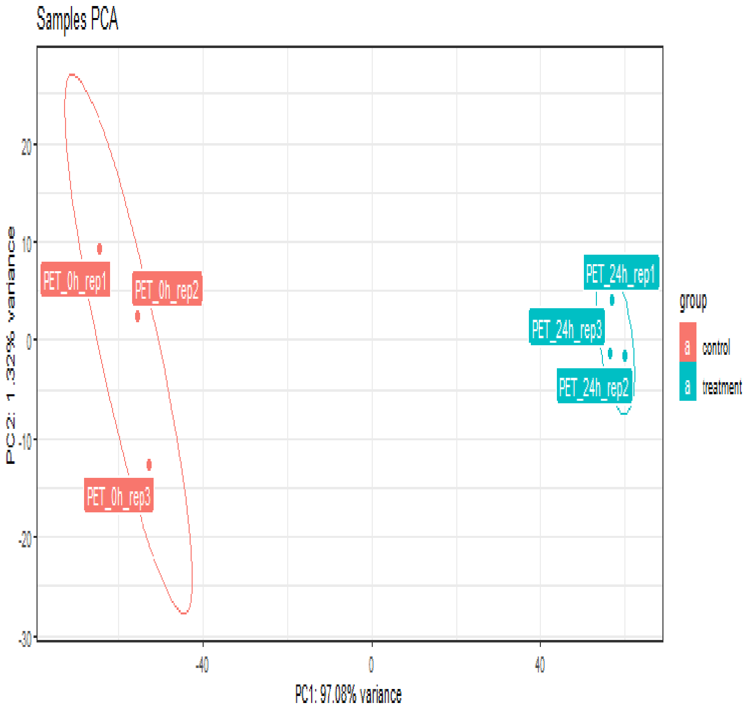

Ab7 - SRP050050_Ethyleen_PET24h

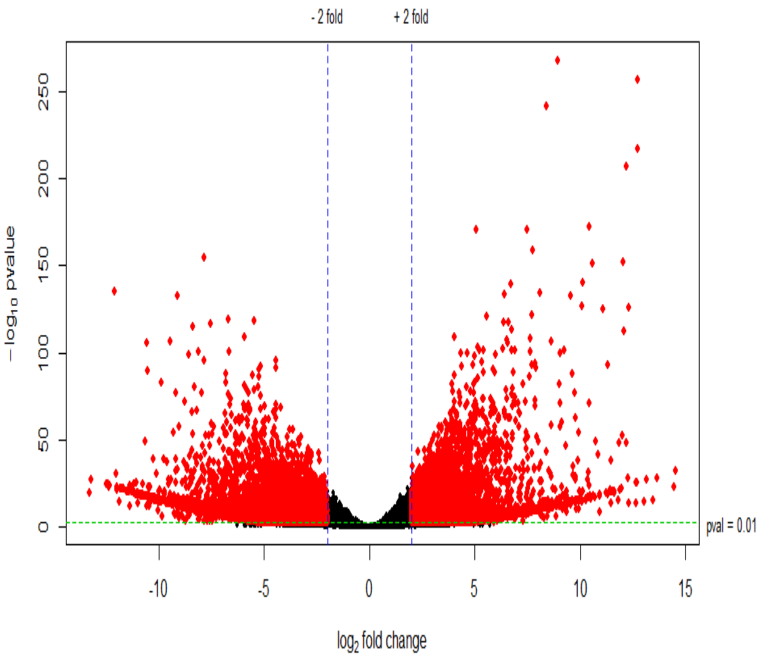

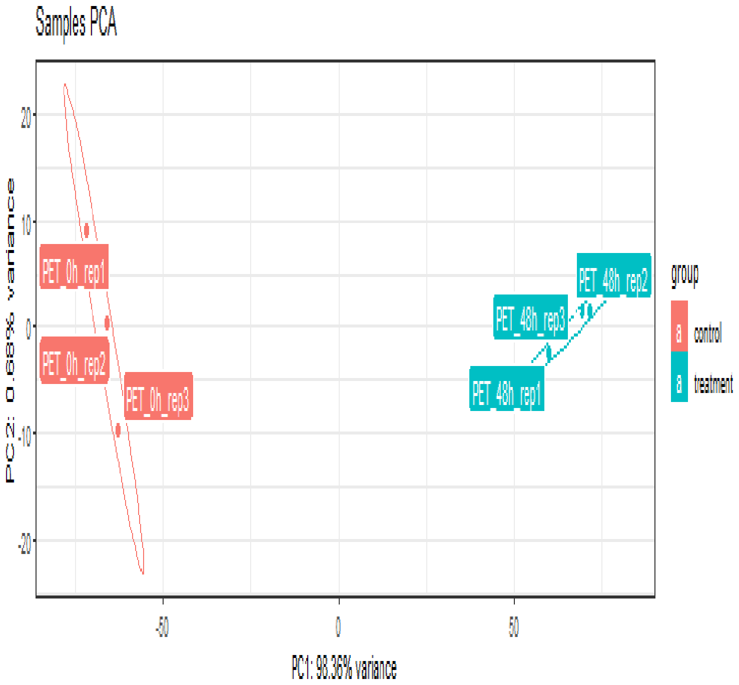

Ab7 - SRP050050_Ethyleen_PET48h

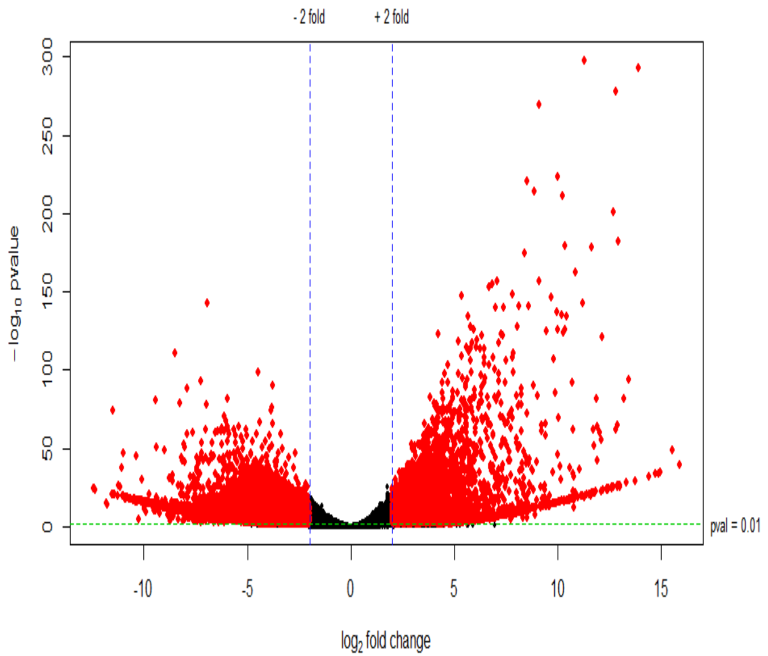

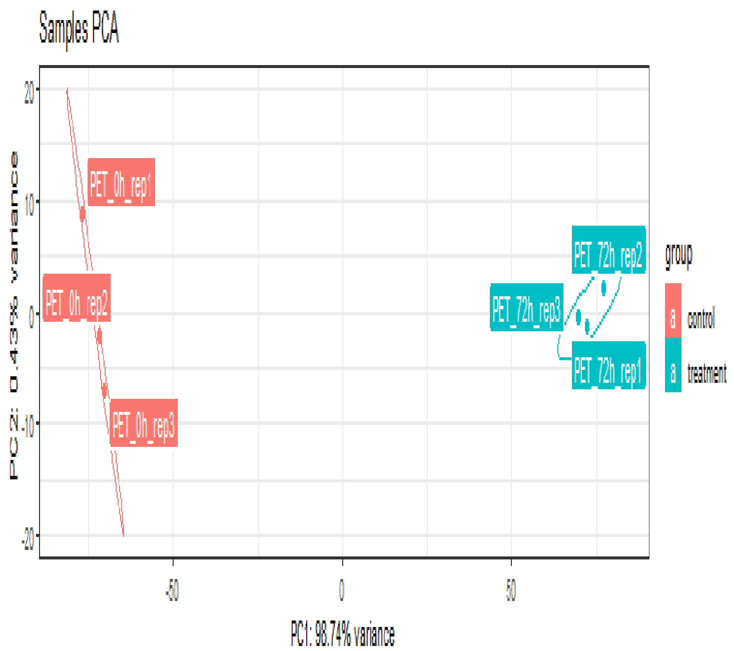

Ab7 - SRP050050_Ethyleen_PET72h

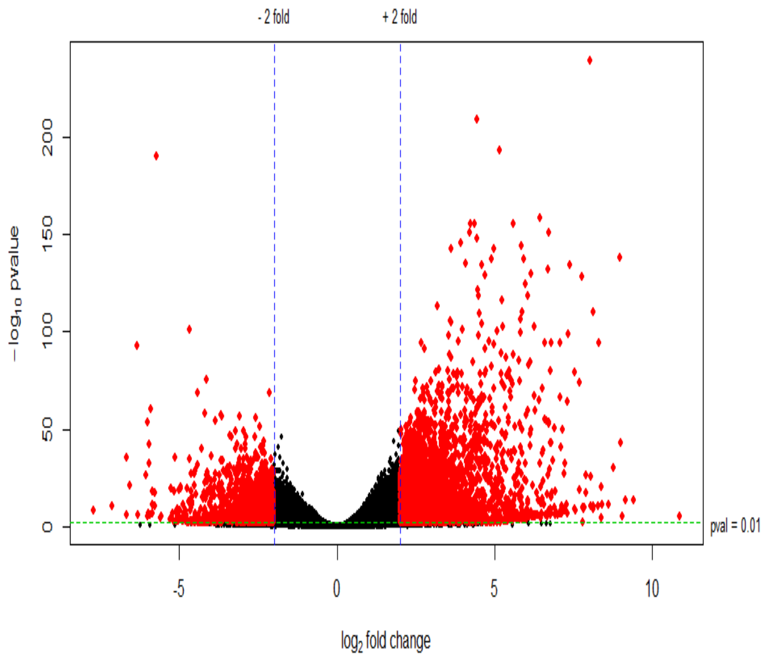

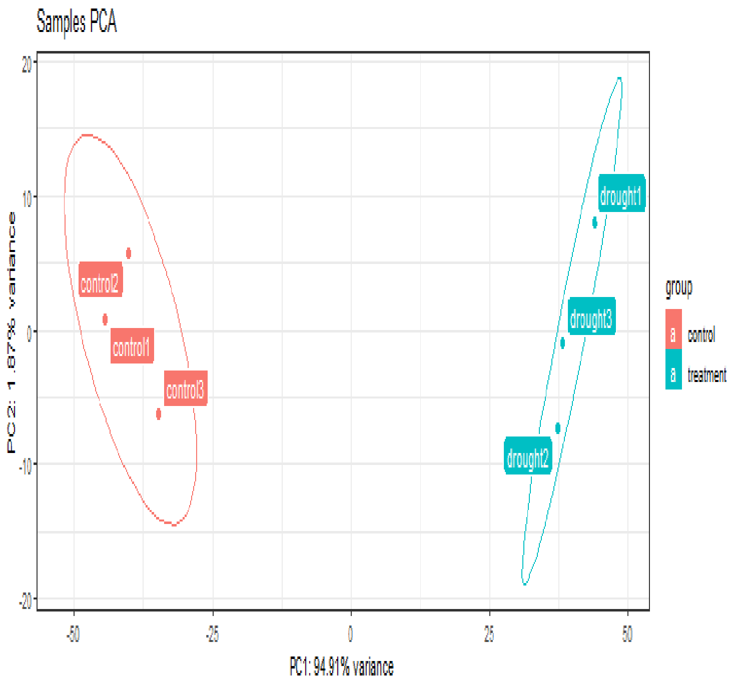

Ab8 - SRP076153_DROUGHT

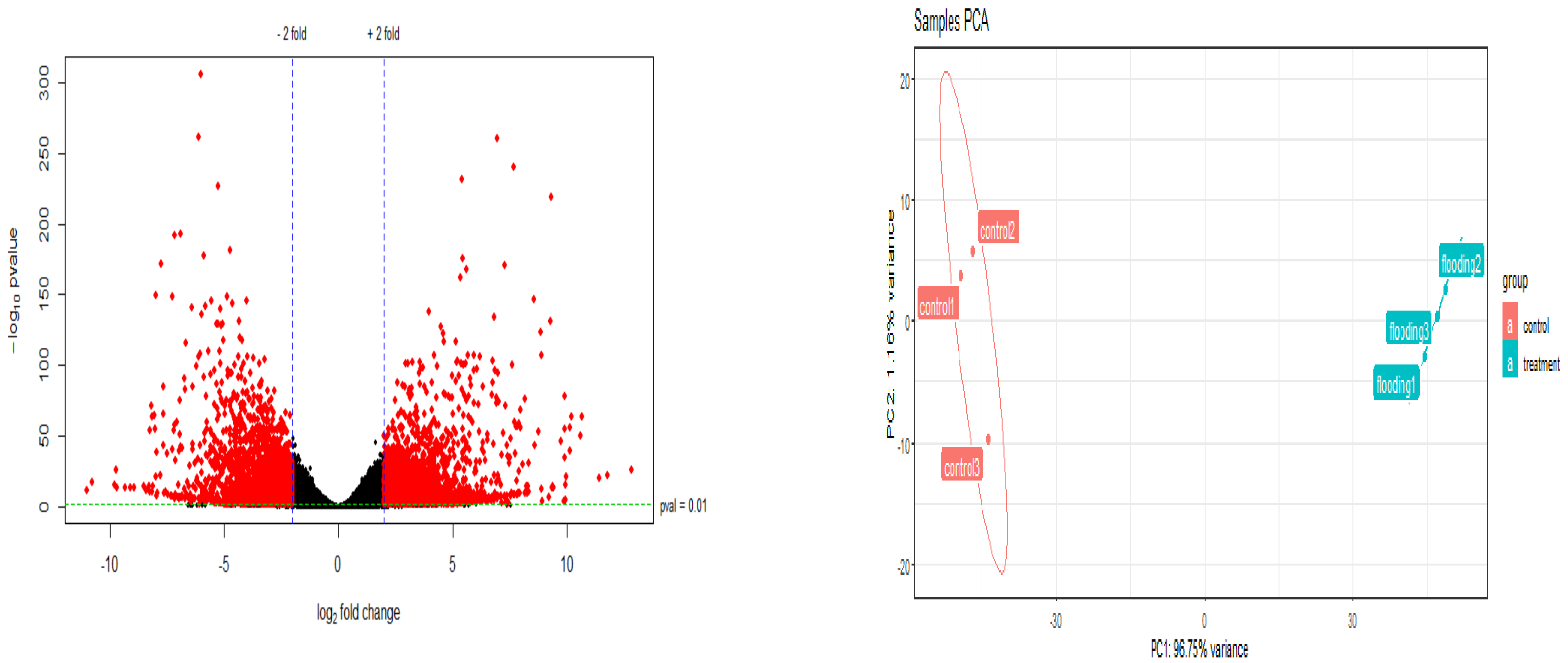

Ab8 - SRP076153_FLOODING

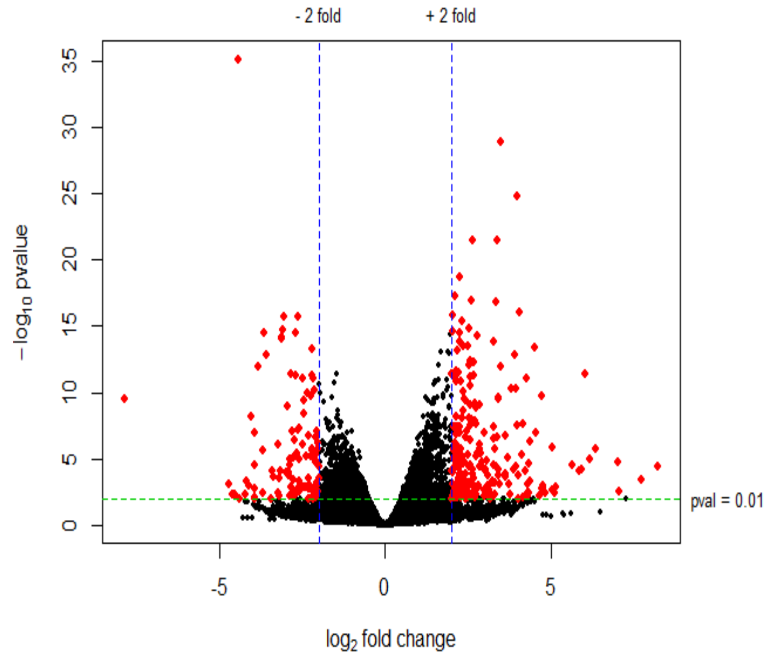

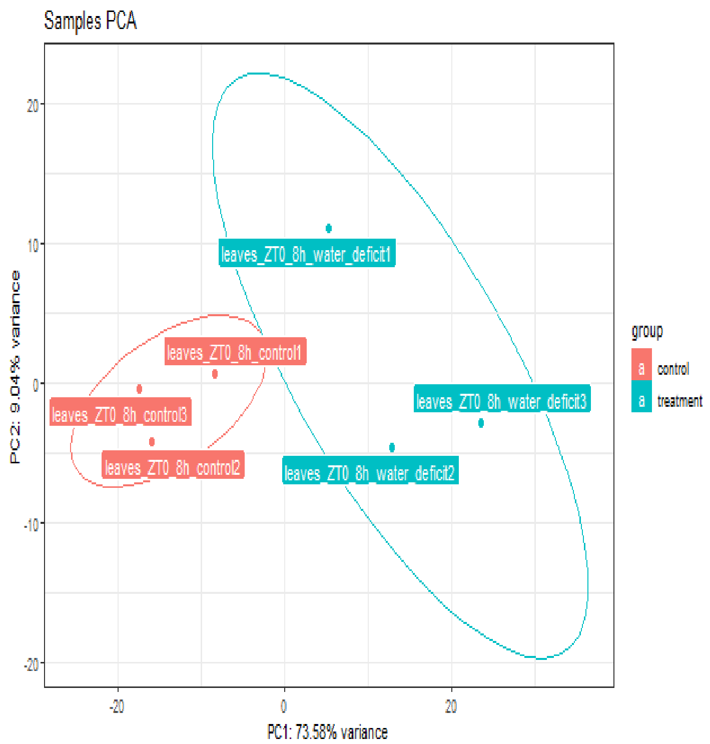

Ab9 - SRP058975_WDef_8h

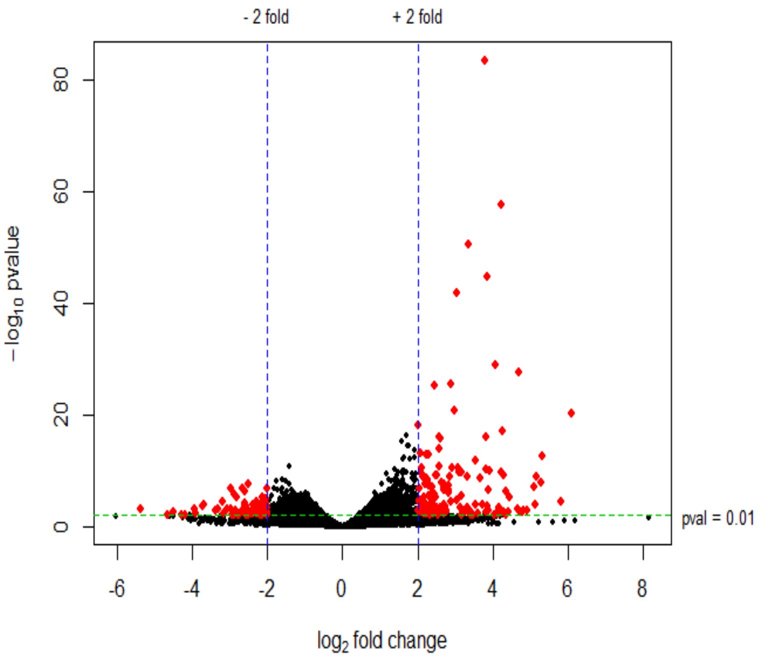

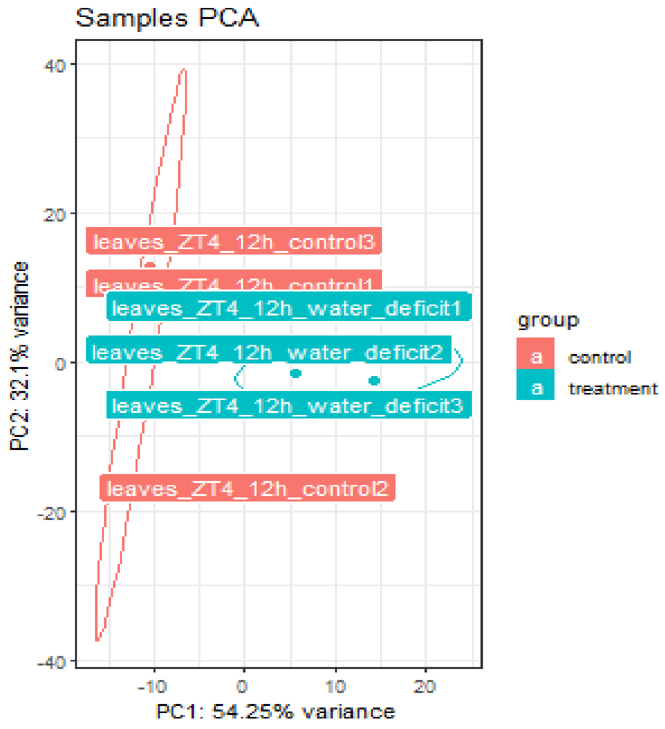

Ab9 - SRP058975_WDef_12h

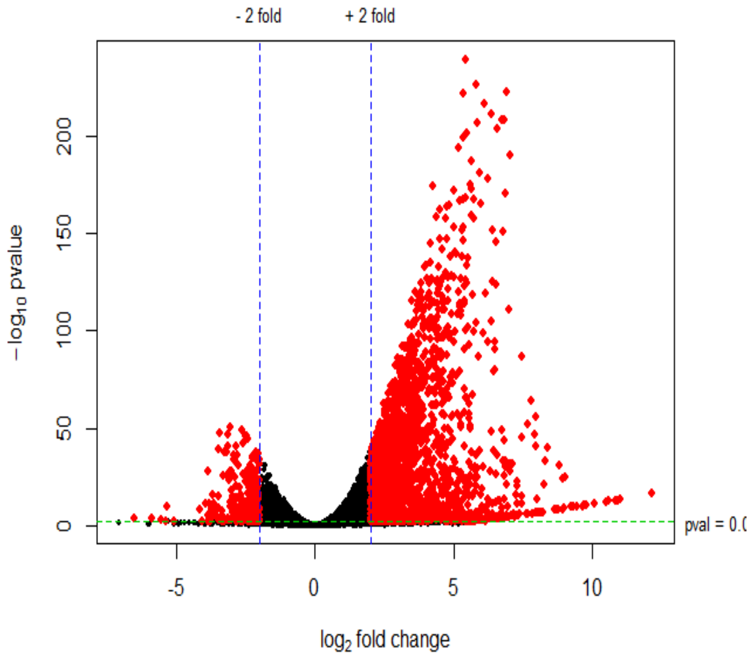

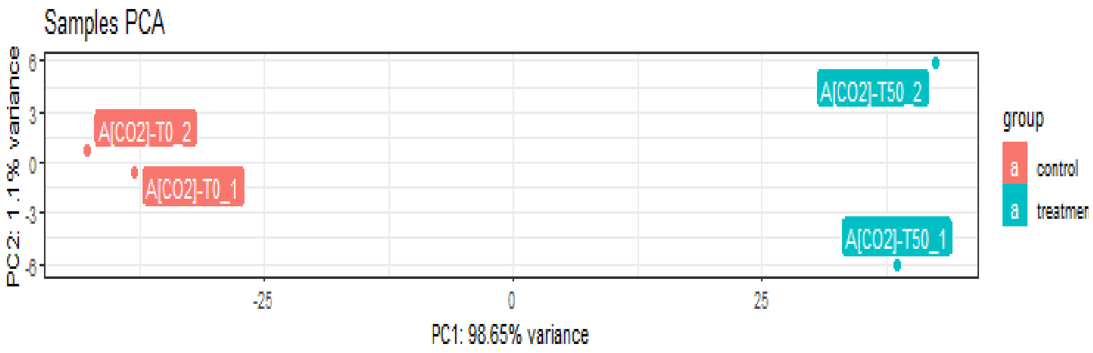

Ab10 - SRP064384_effectDrought_ambientCO2

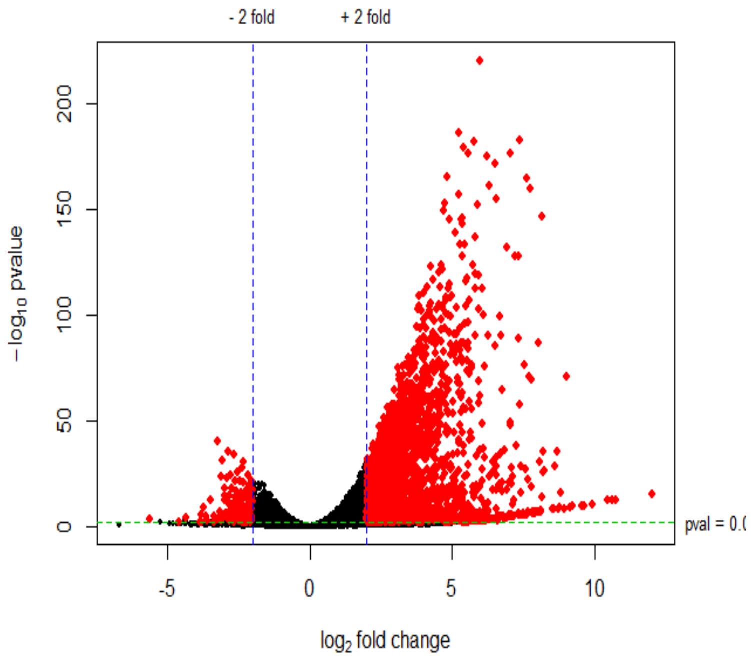

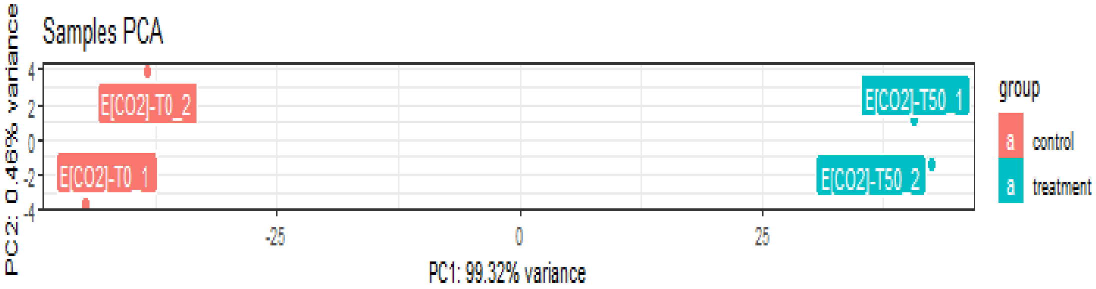

Ab10 - SRP064384_effectDrought_elevatedCO2

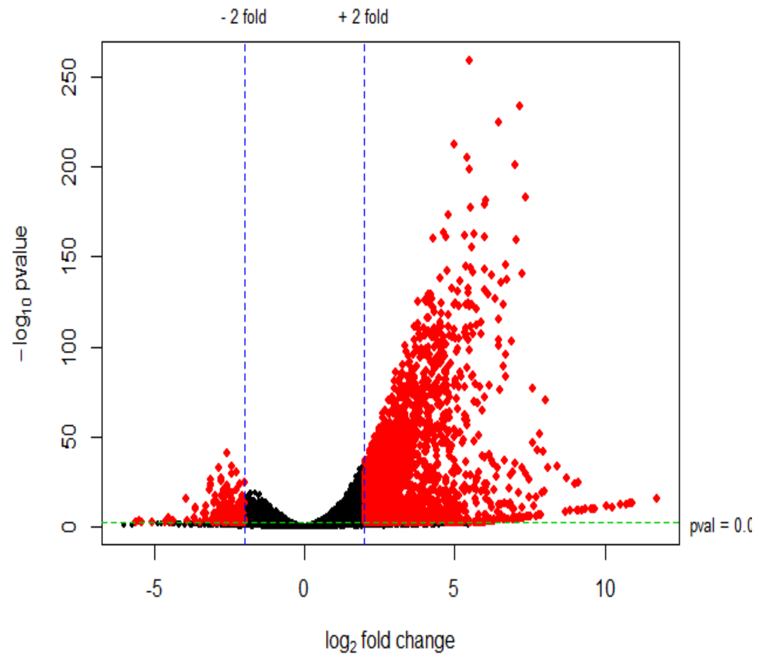

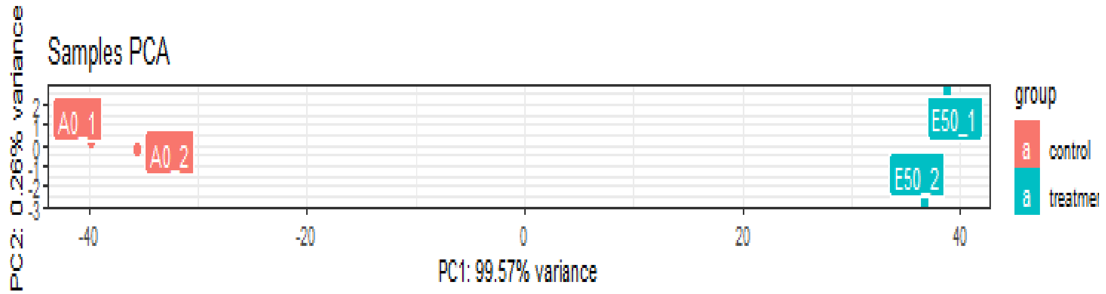

Ab10 - SRP064384_mixedeffect_AmbientnoD_Elevated_D50minD

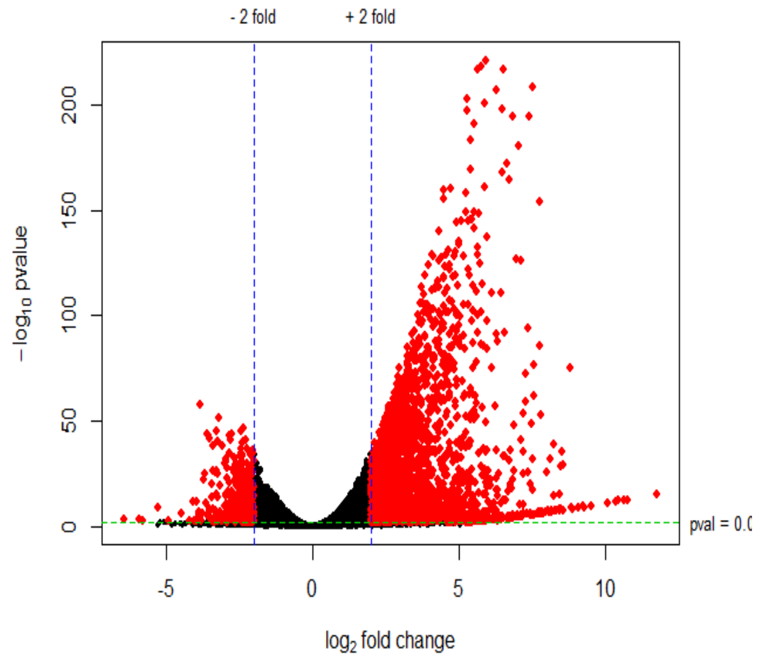

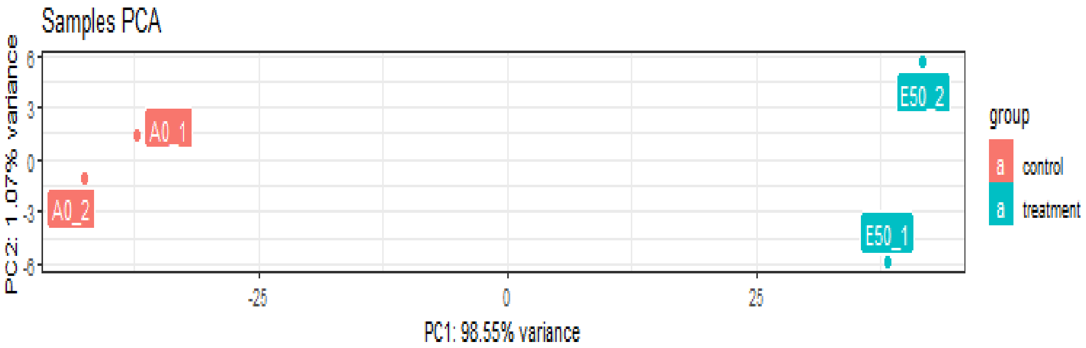

Ab10 - SRP064384_mixedeffect_ElevatednoD.VS.Ambient_D50

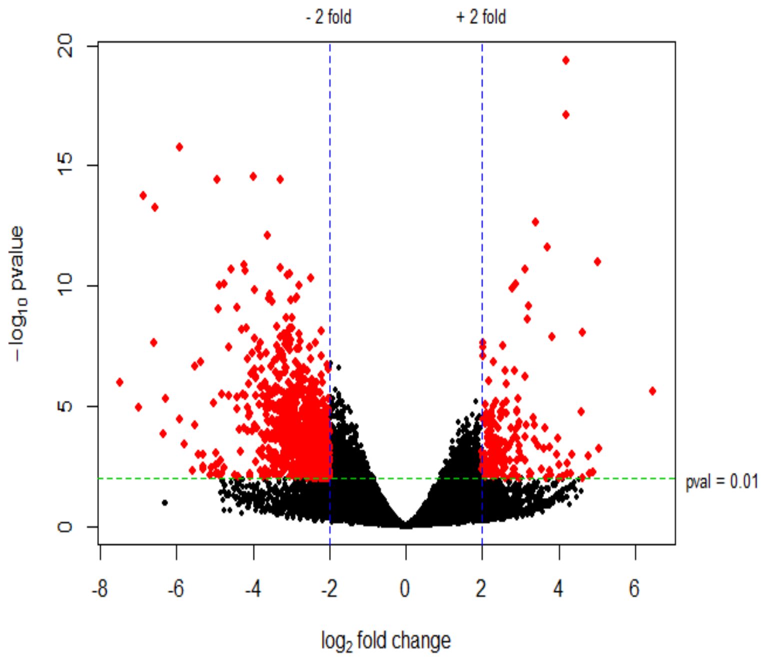

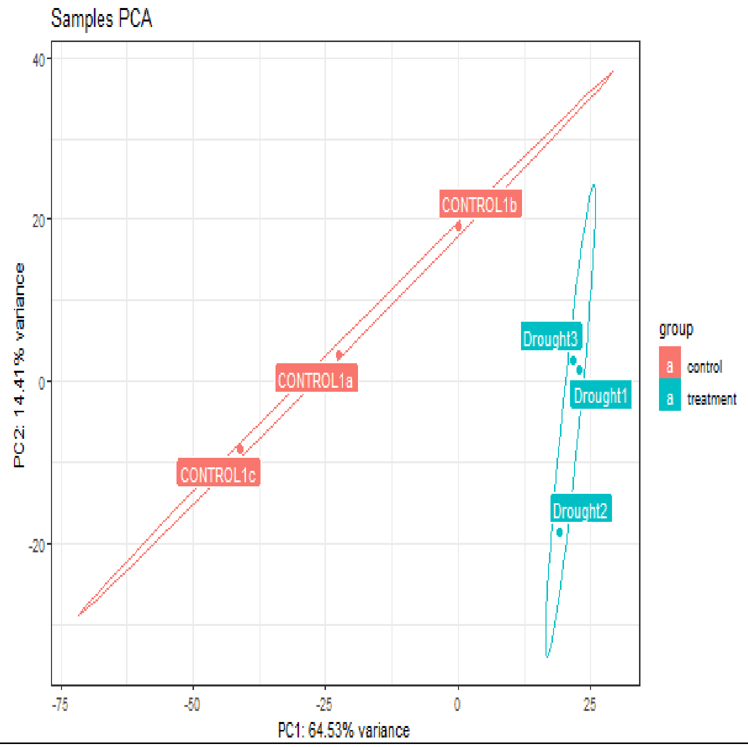

Ab11 - SRP105922-965_DROUGHT

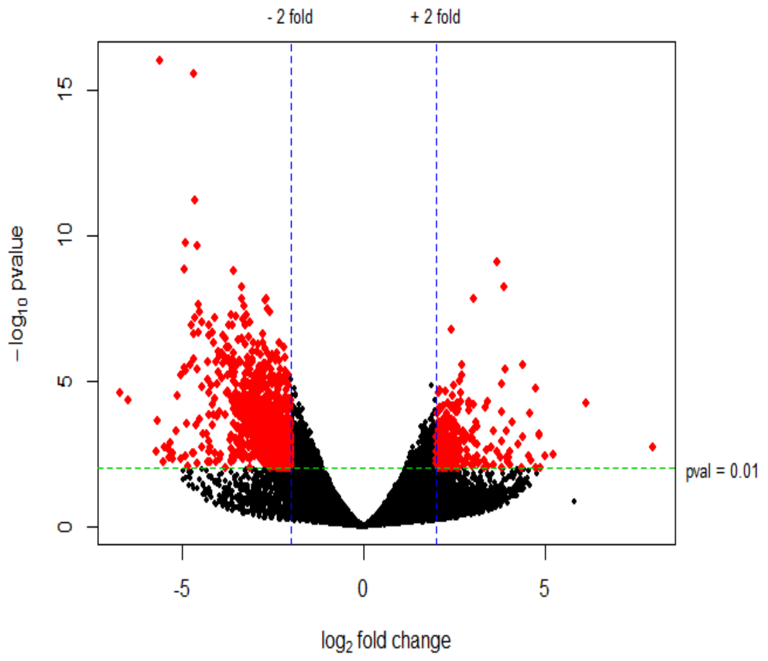

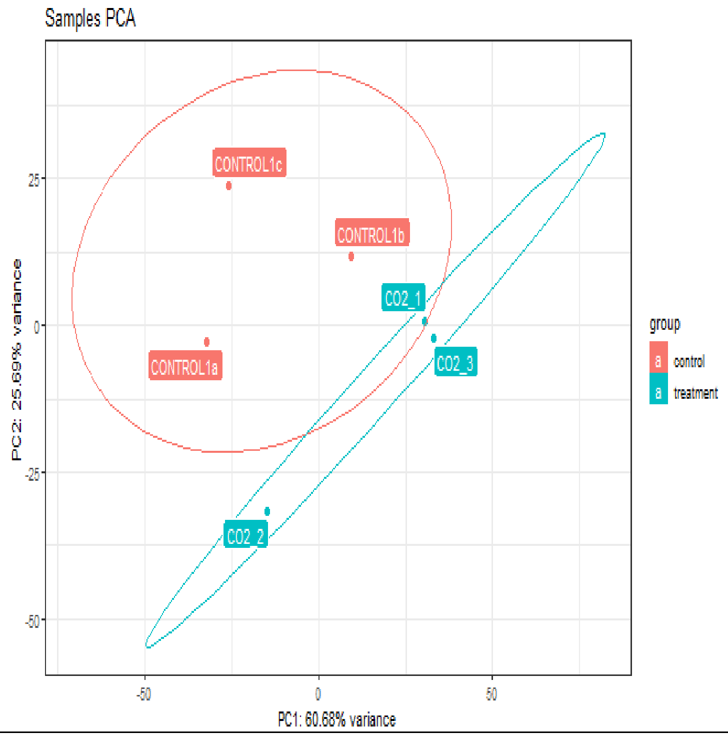

Ab11 - SRP105922-965_HighCO2

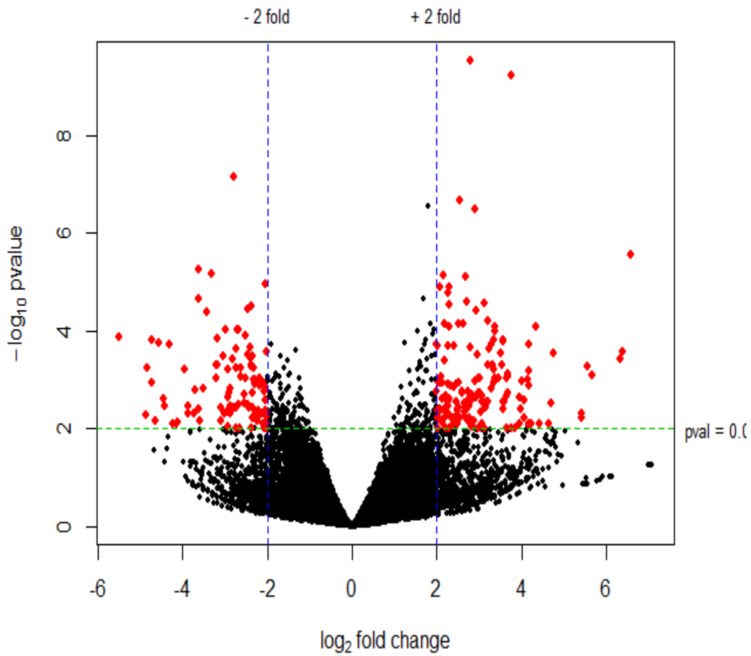

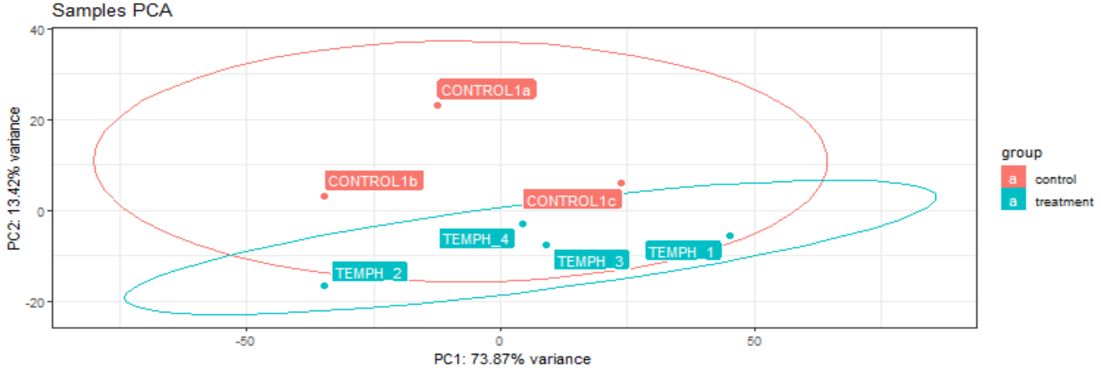

Ab11 - SRP105922-965_highT

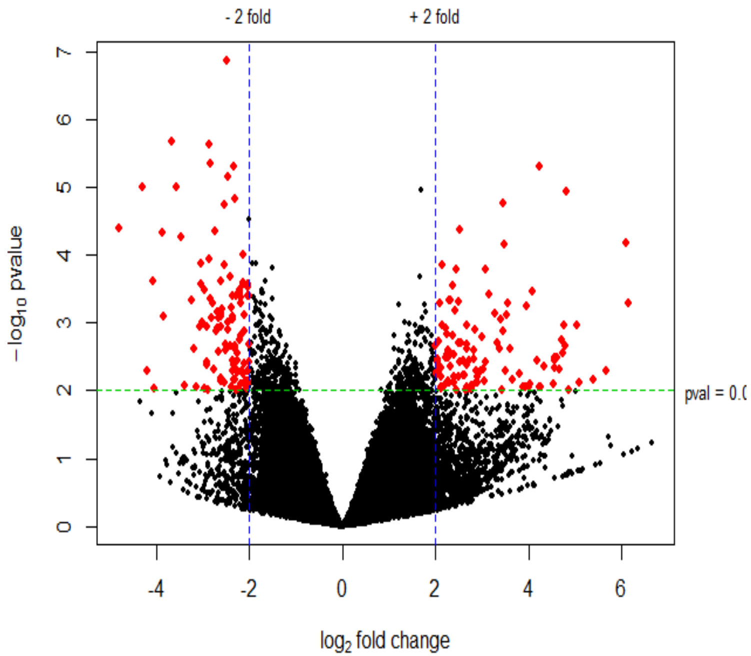

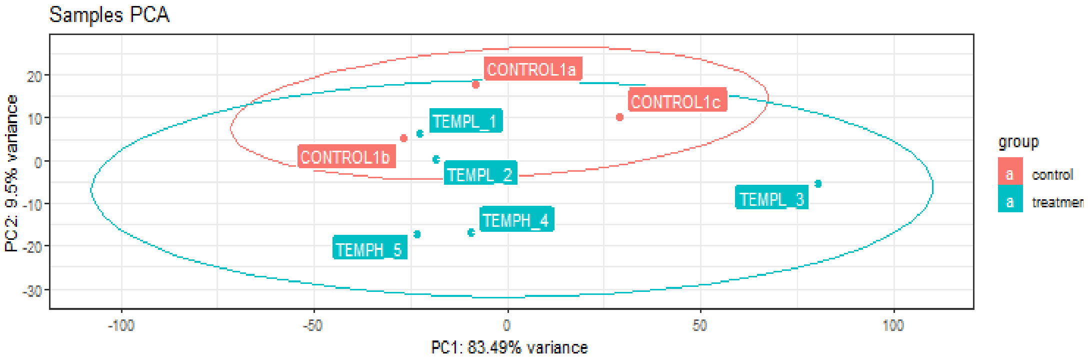

Ab11 - SRP105922-965_lowT

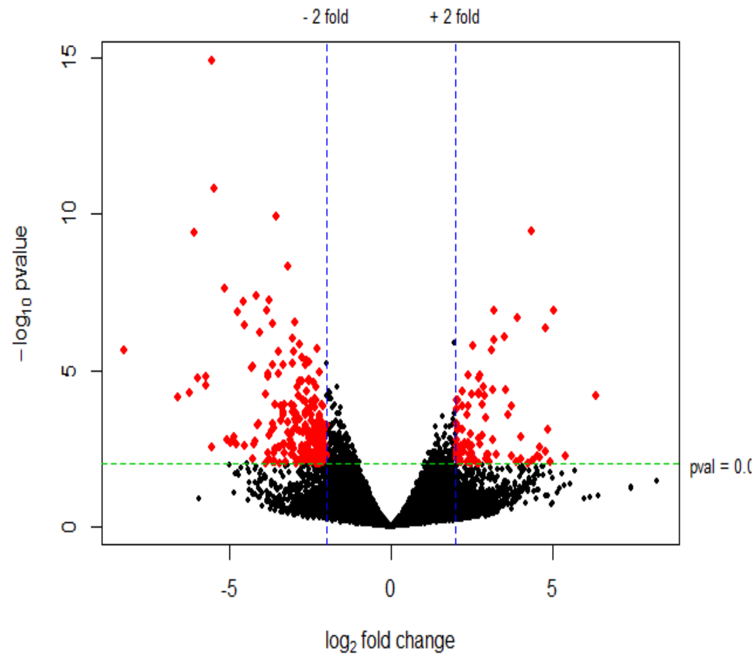

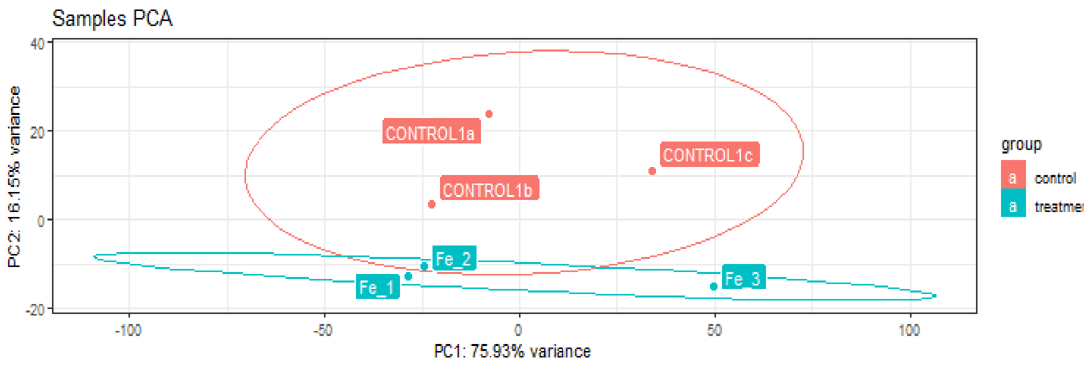

Ab11 - SRP105922-965_minusFe

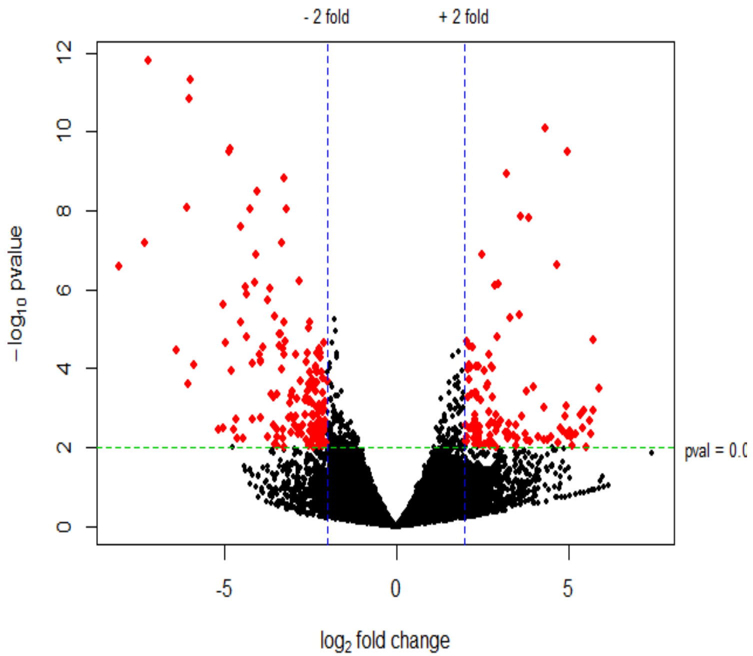

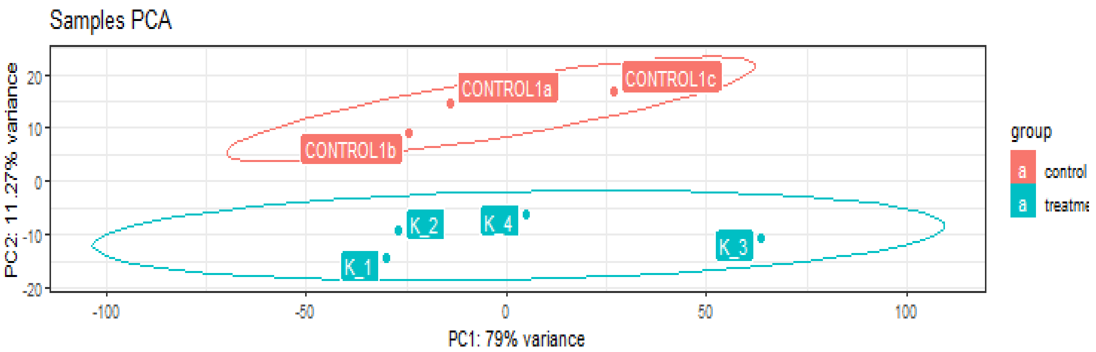

Ab11 - SRP105922-965_minusK

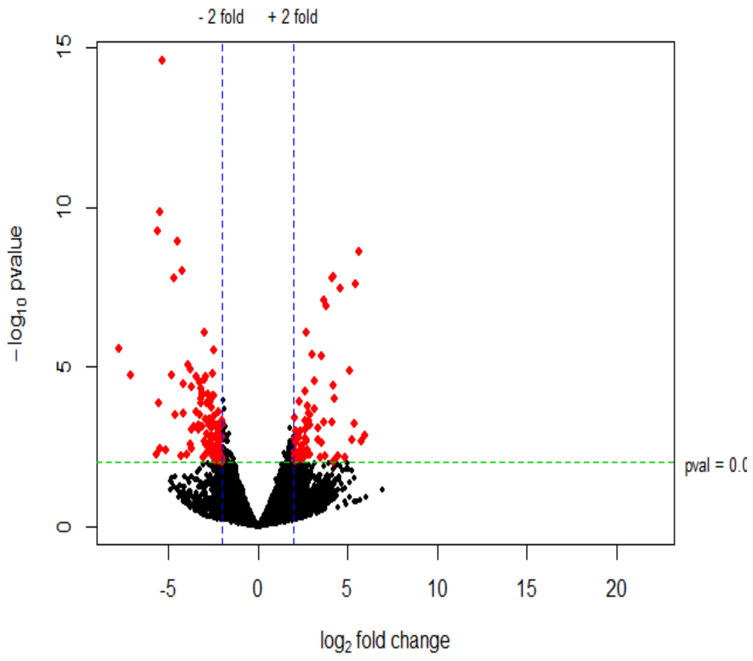

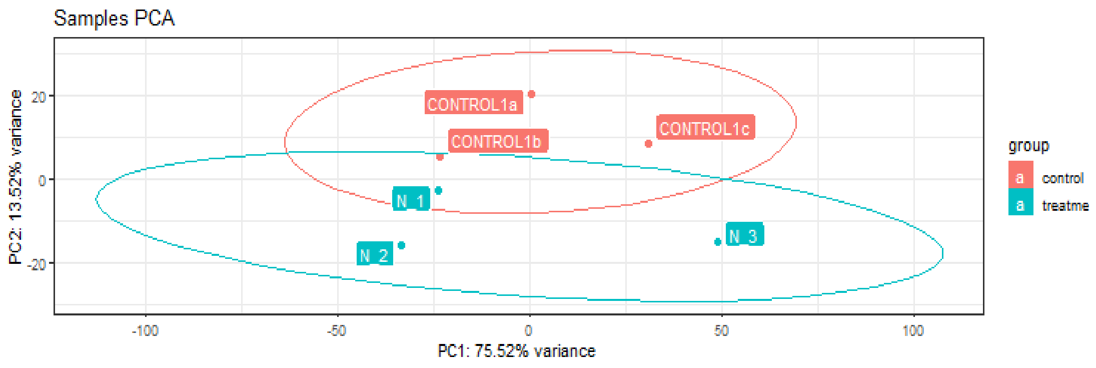

Ab11 - SRP105922-965_minusN

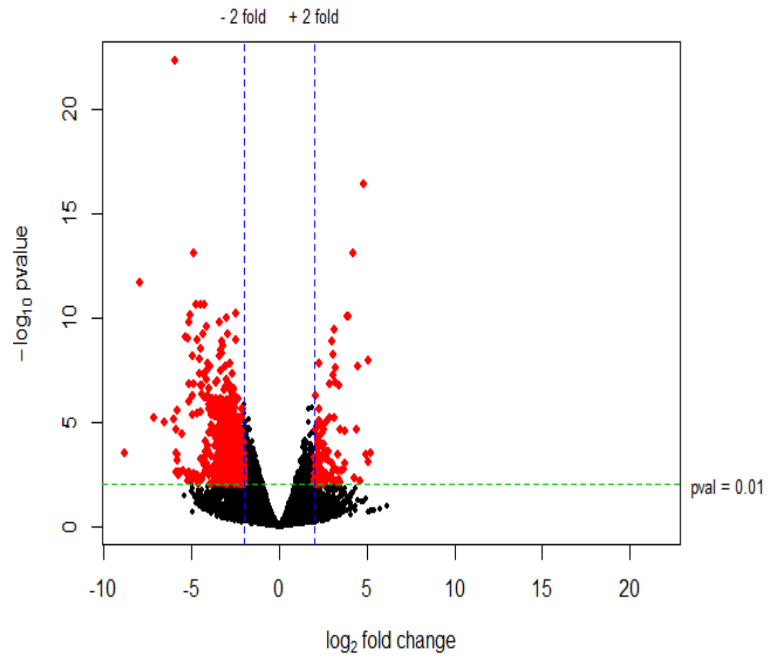

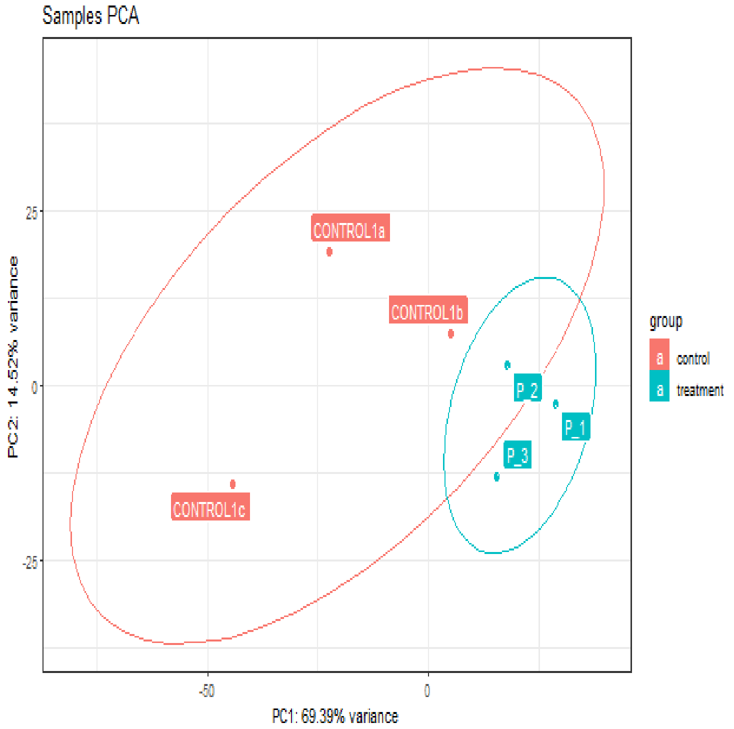

Ab11 - SRP105922-965_minusP

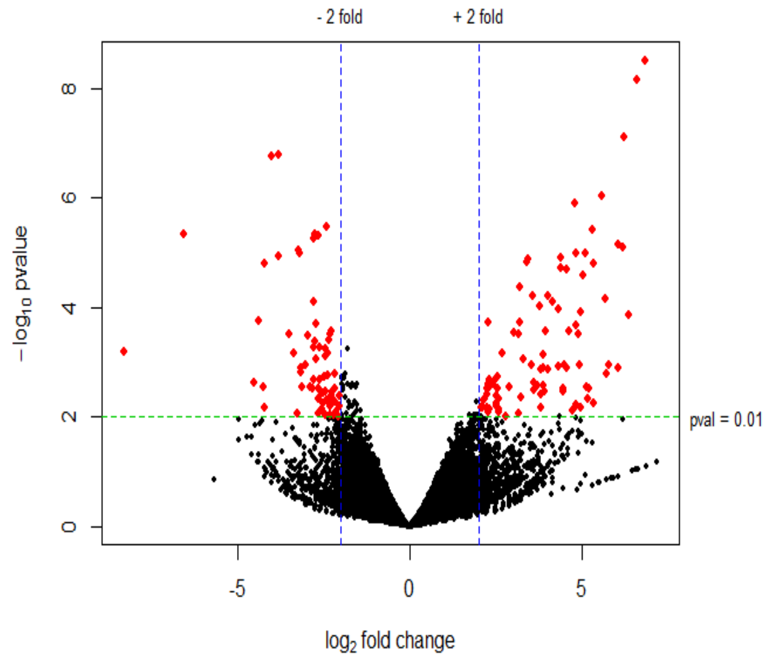

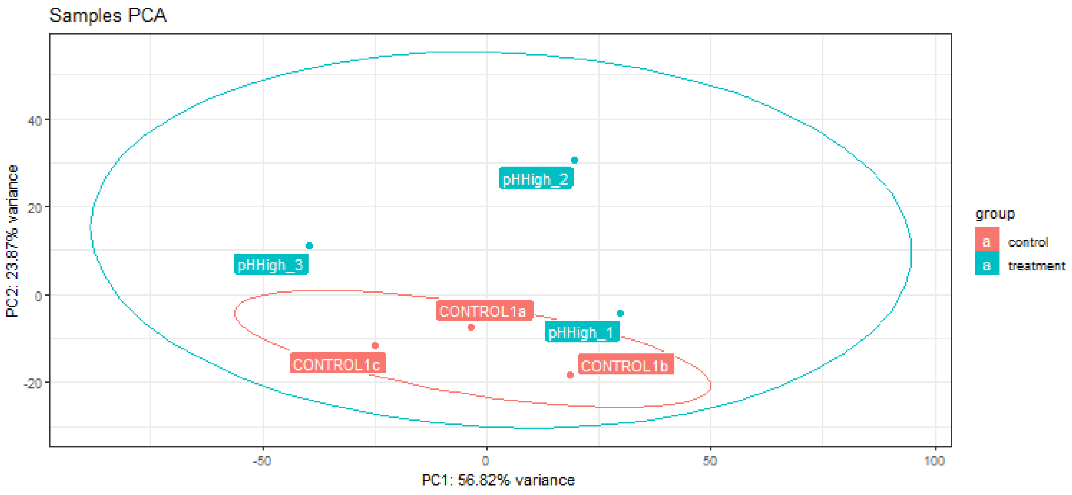

Ab11 - SRP105922-965_pHHigh

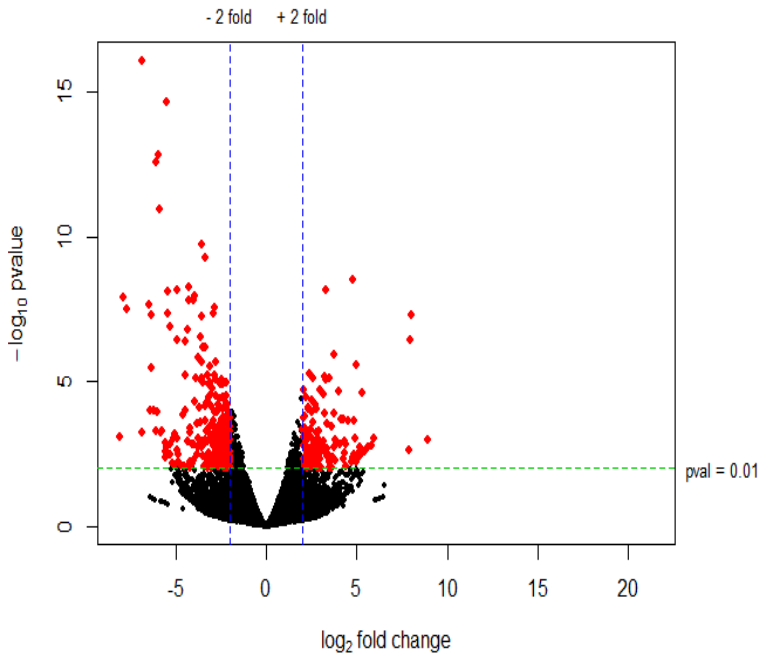

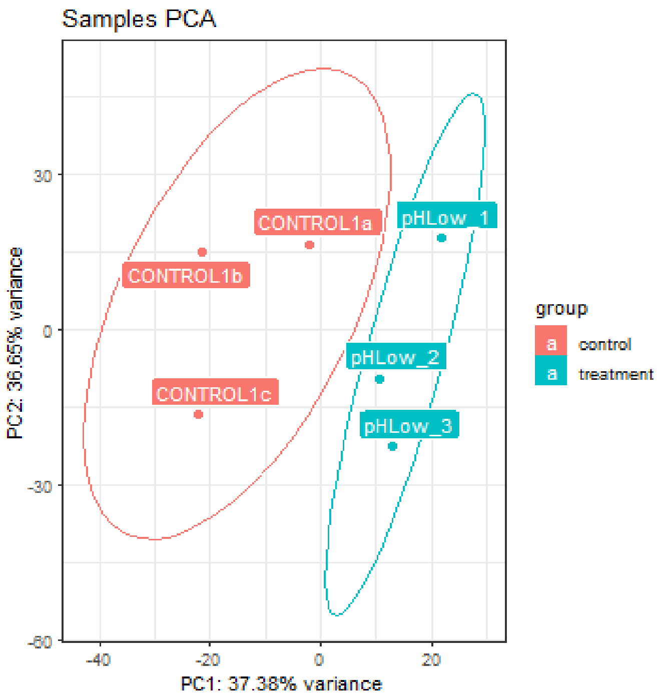

Ab11 - SRP105922-965_pHLow

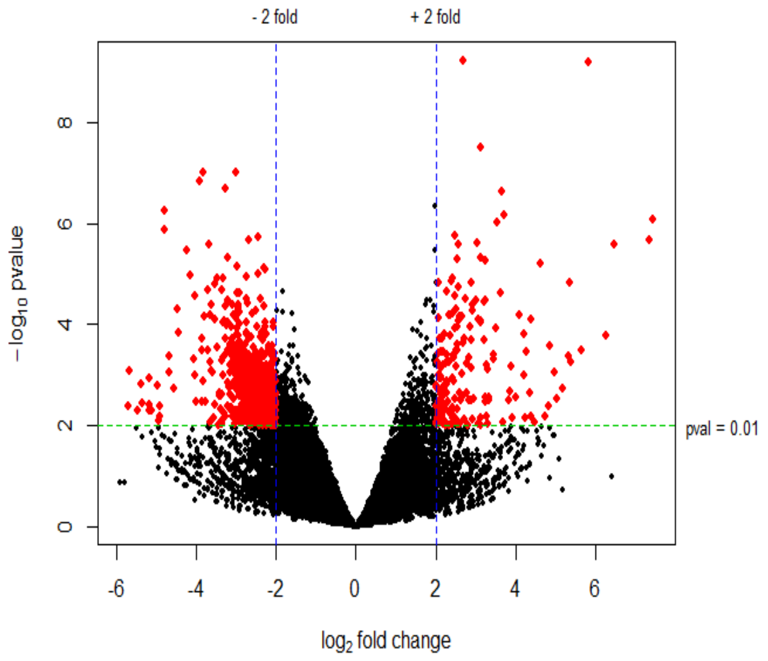

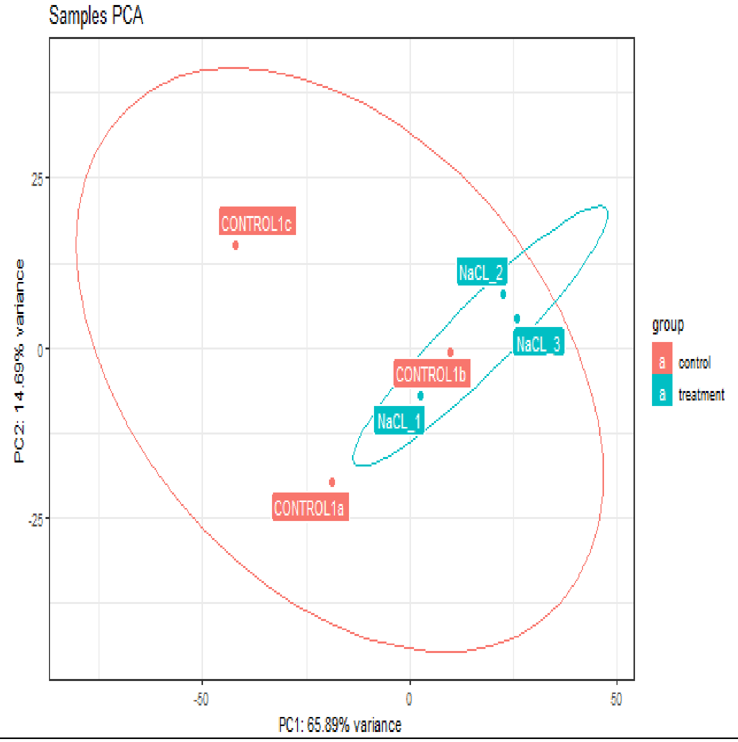

Ab11 - SRP105922-965_Salinity

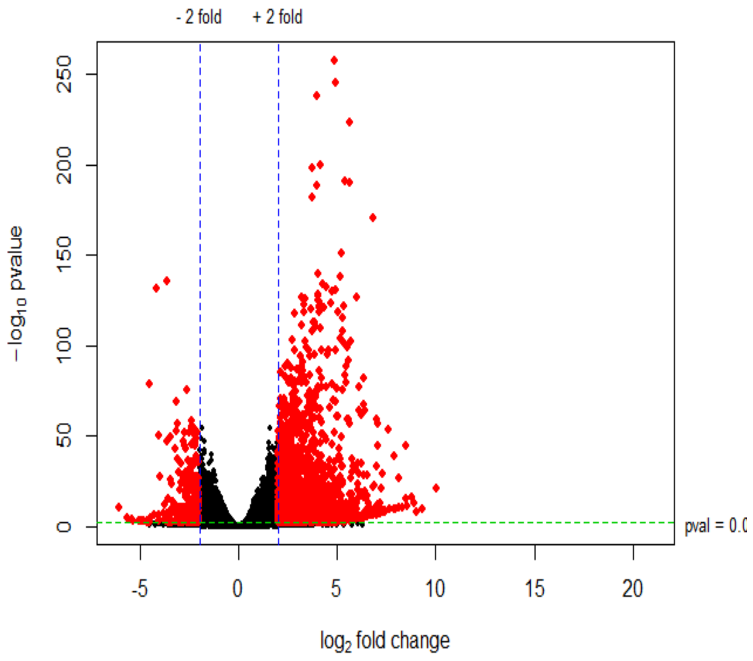

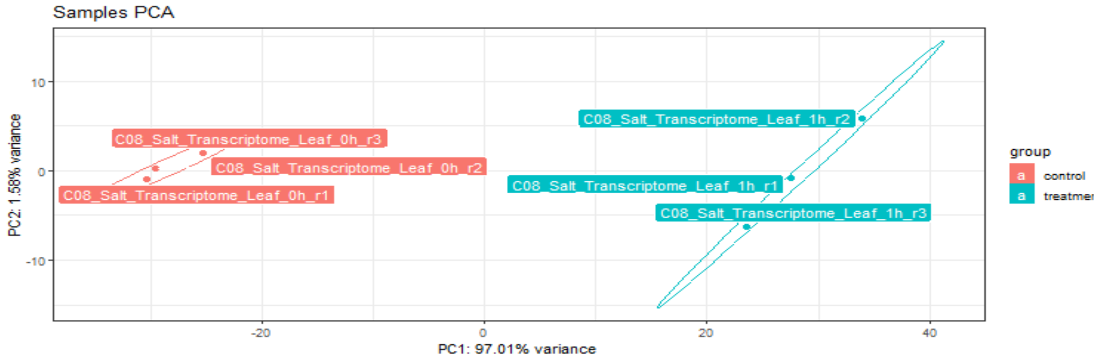

Ab12 - SRP132150_SALT_LEAVES_1h

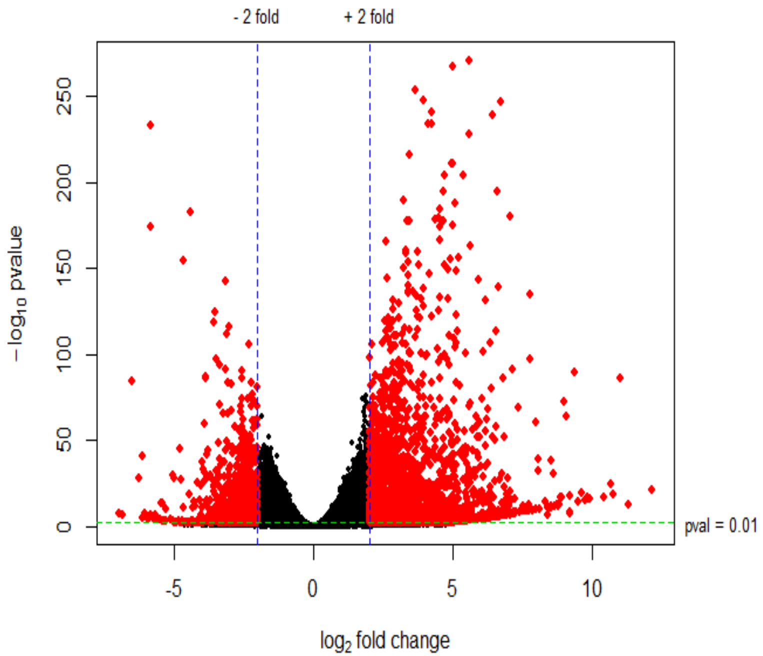

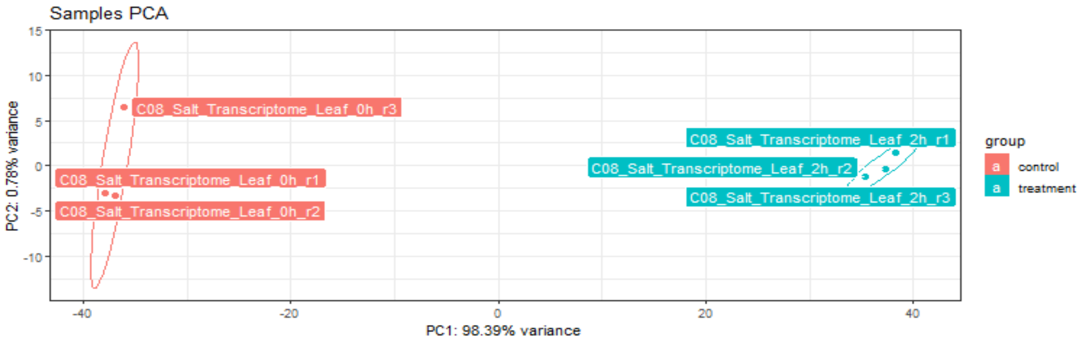

Ab12 - SRP132150_SALT_LEAVES_2h

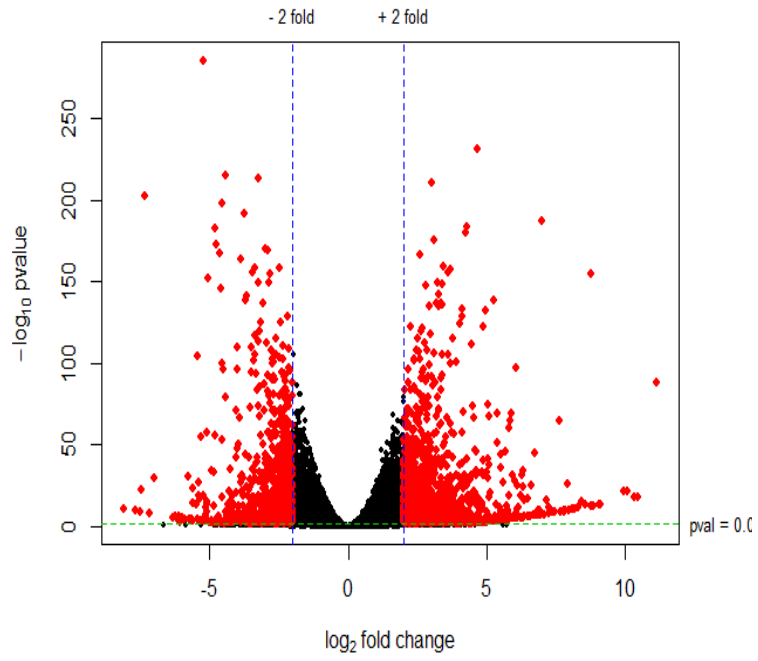

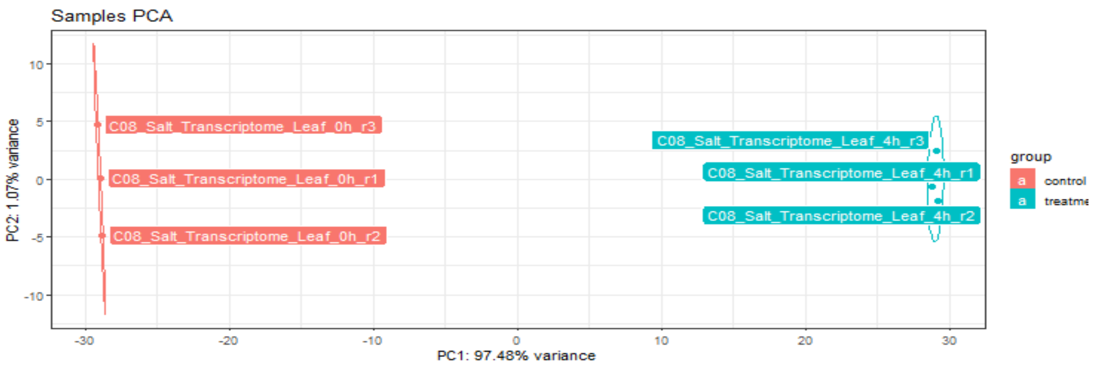

Ab12 - SRP132150_SALT_LEAVES_4h

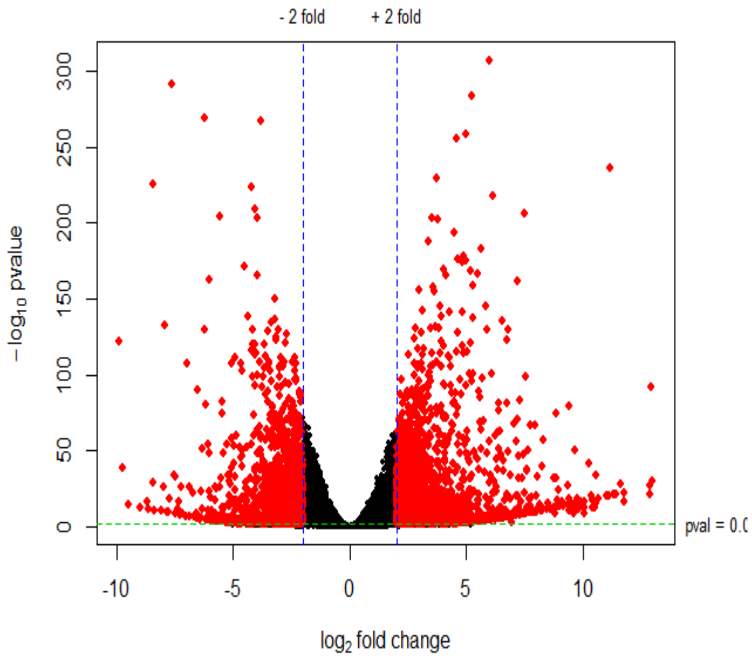

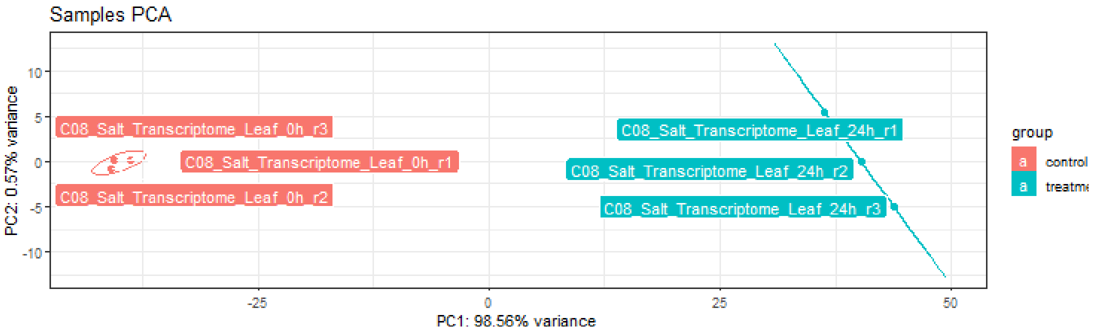

Ab12 - SRP132150_SALT_LEAVES_24h

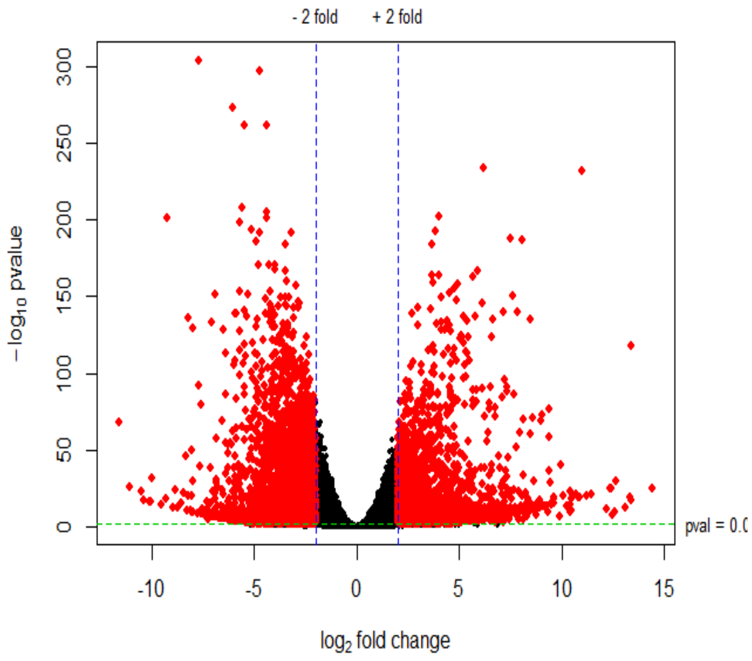

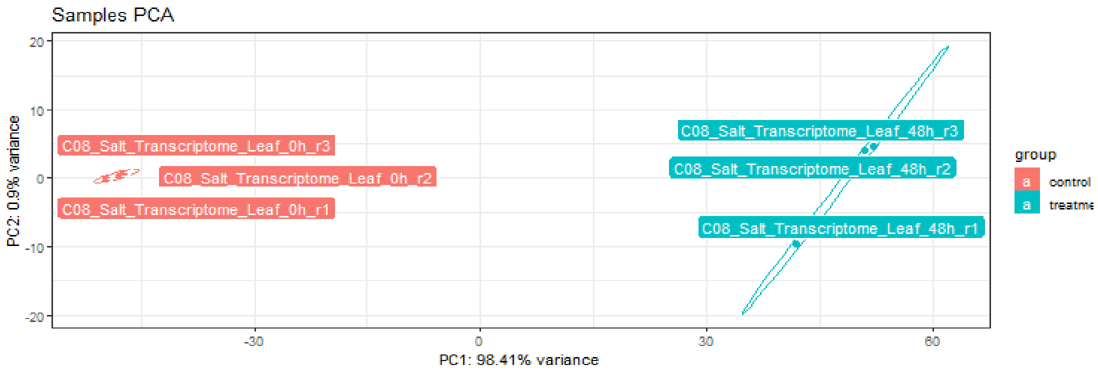

Ab12 - SRP132150_SALT_LEAVES_48h

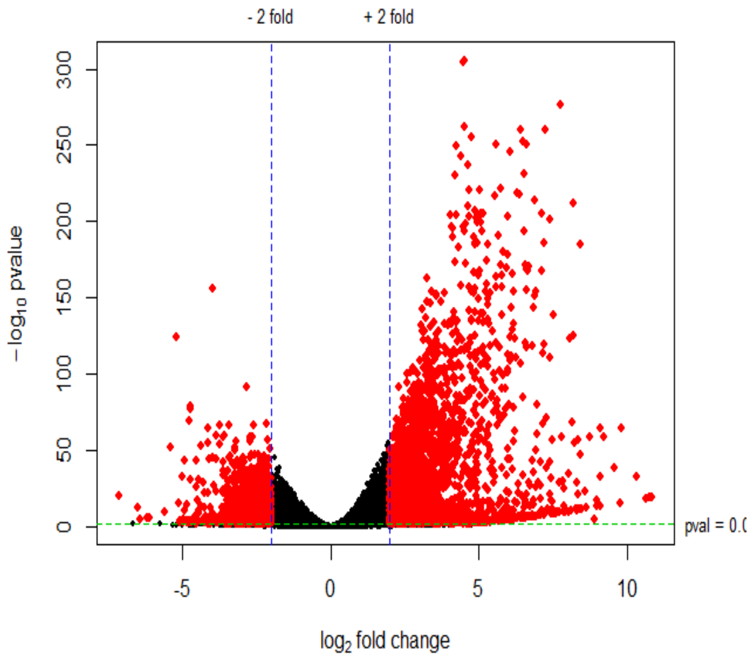

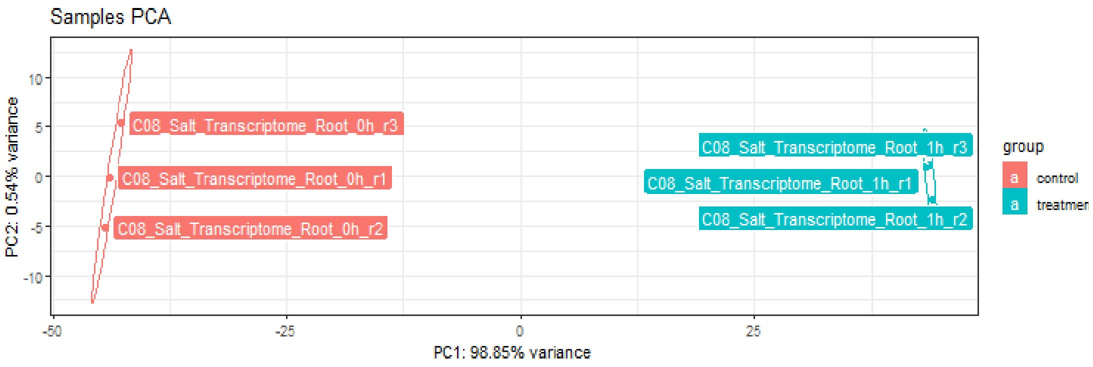

Ab12 - SRP132150_SALT_ROOT_1h

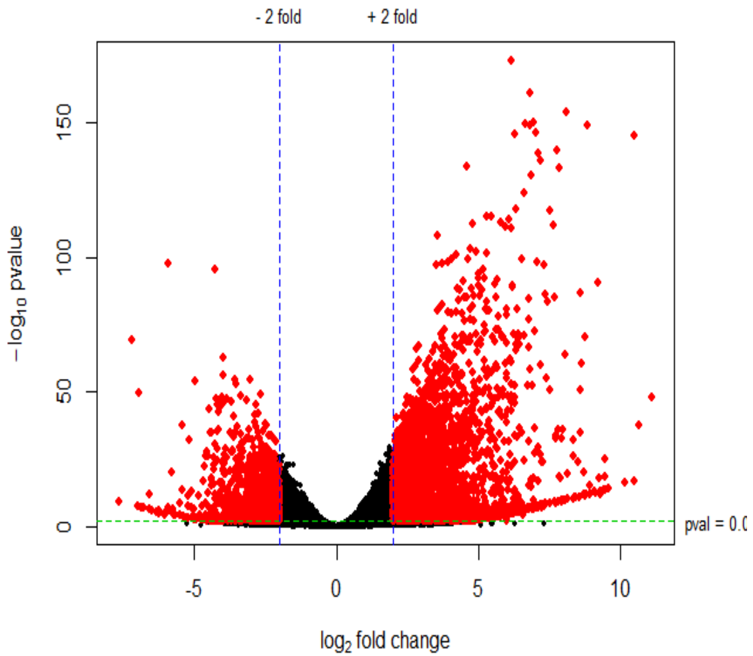

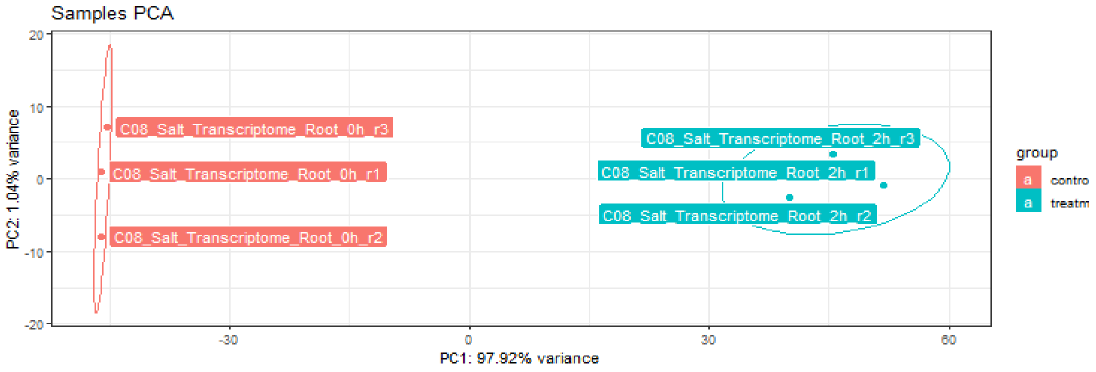

Ab12 - SRP132150_SALT_ROOT_2h

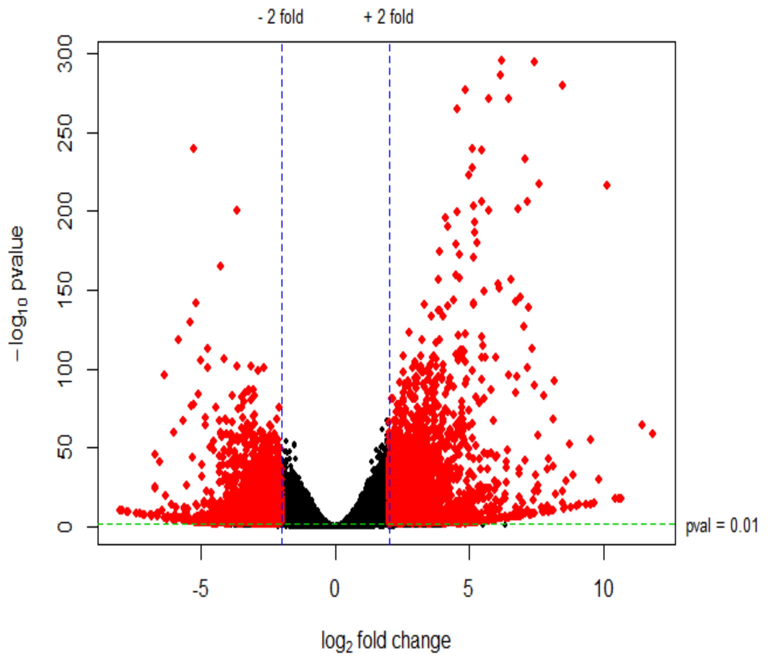

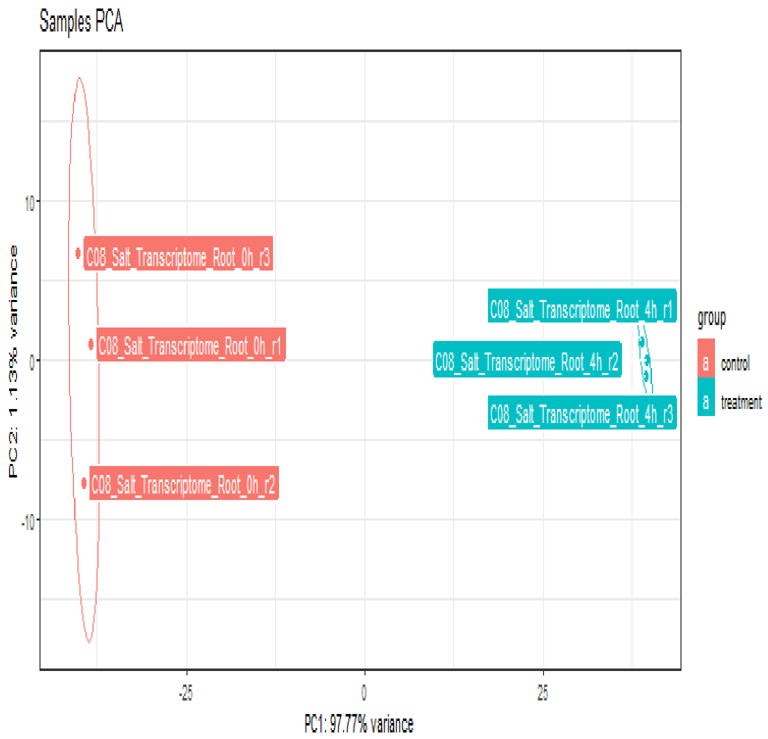

Ab12 - SRP132150_SALT_ROOT_4h

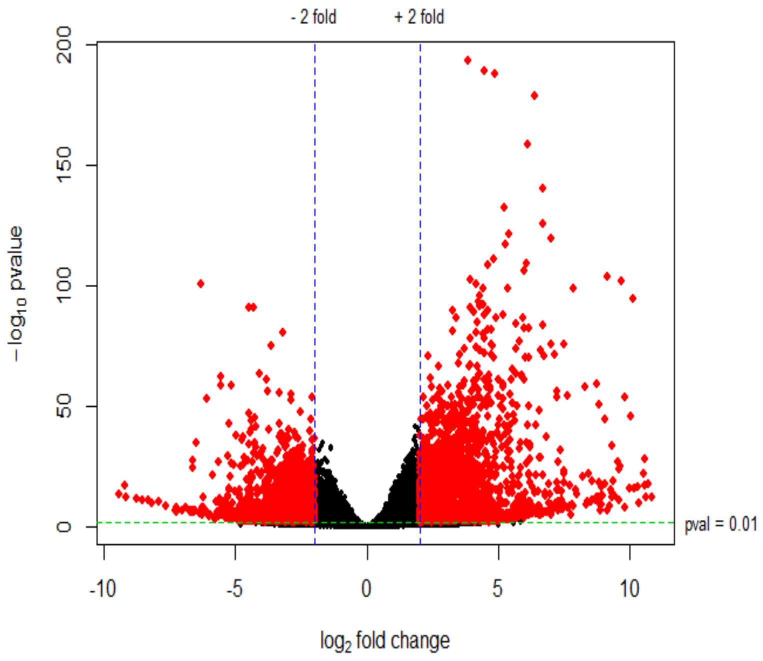

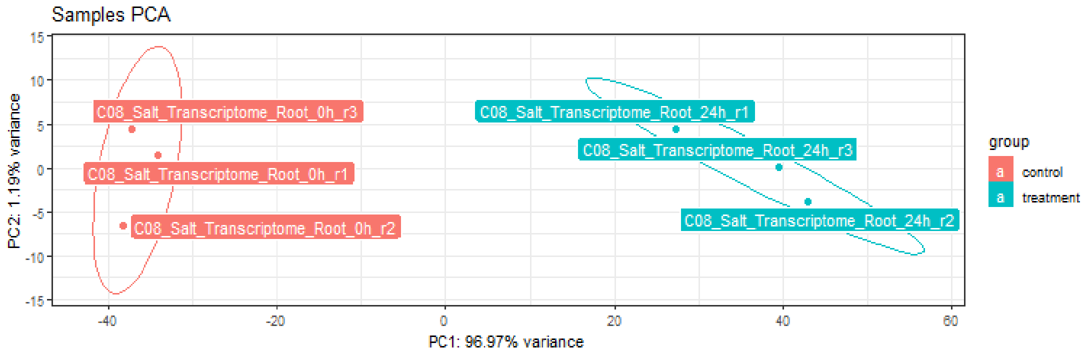

Ab12 - SRP132150_SALT_ROOT_24h

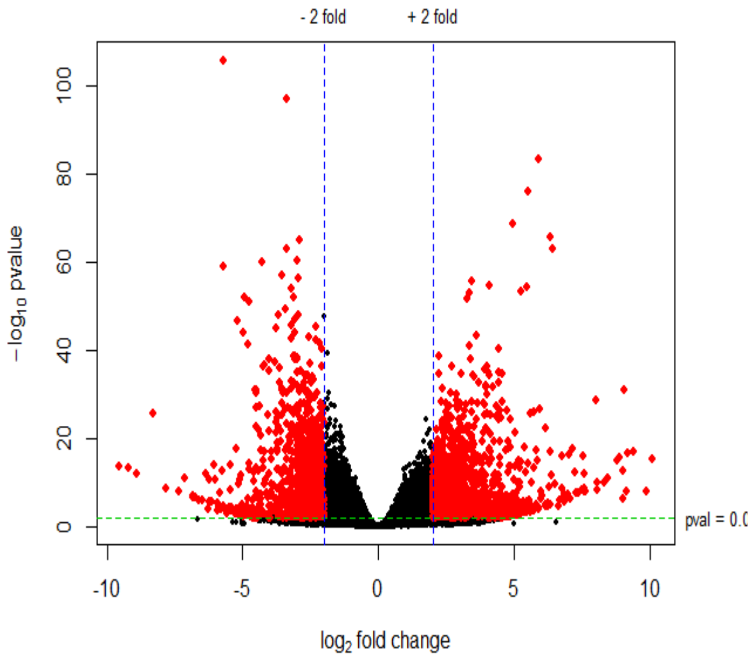

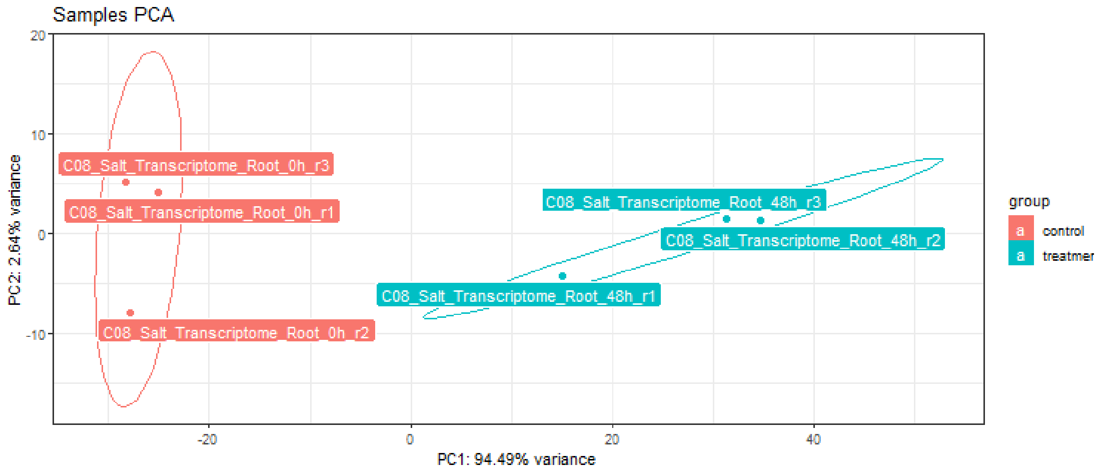

Ab12 - SRP132150_SALT_ROOT_48h

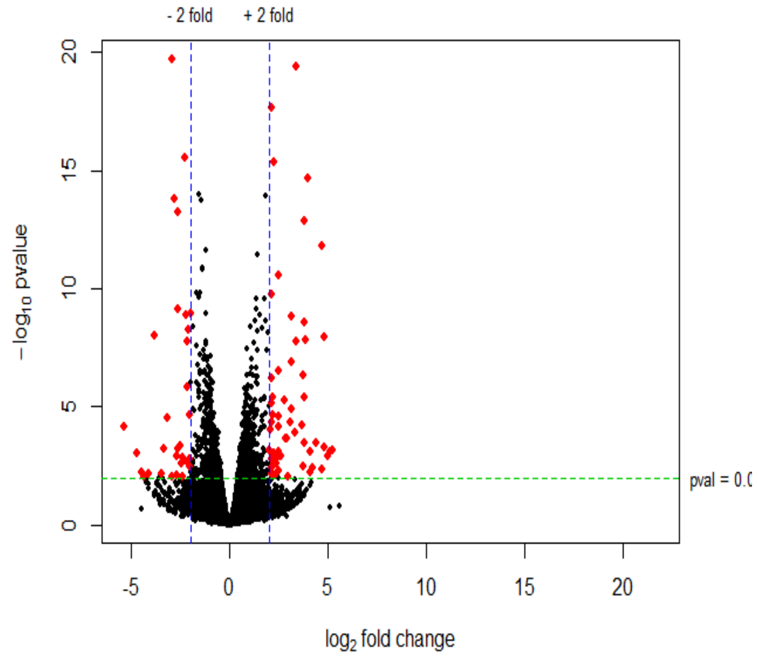

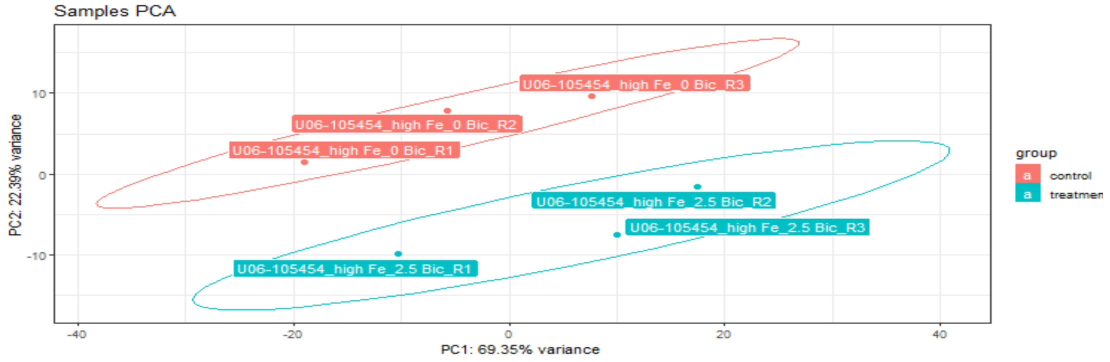

Ab13 - SRP108540_HighFe_2.5BIC_454

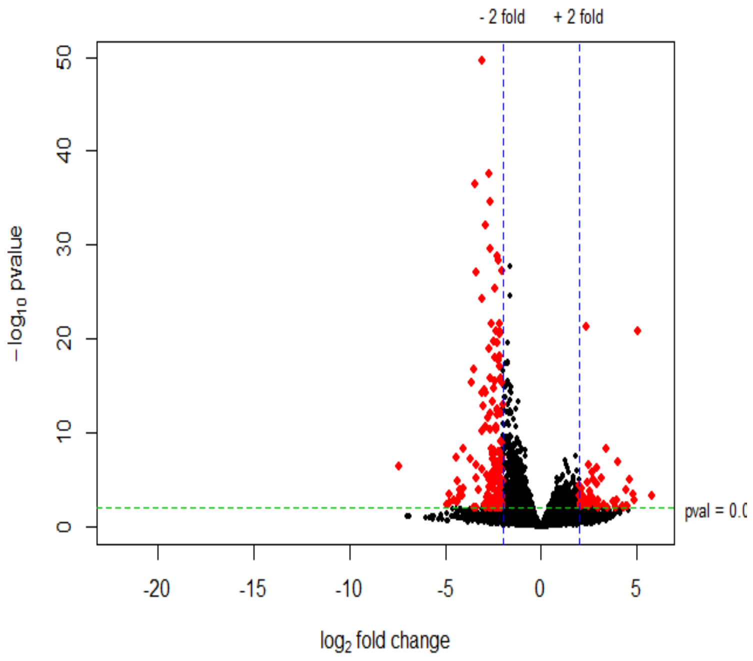

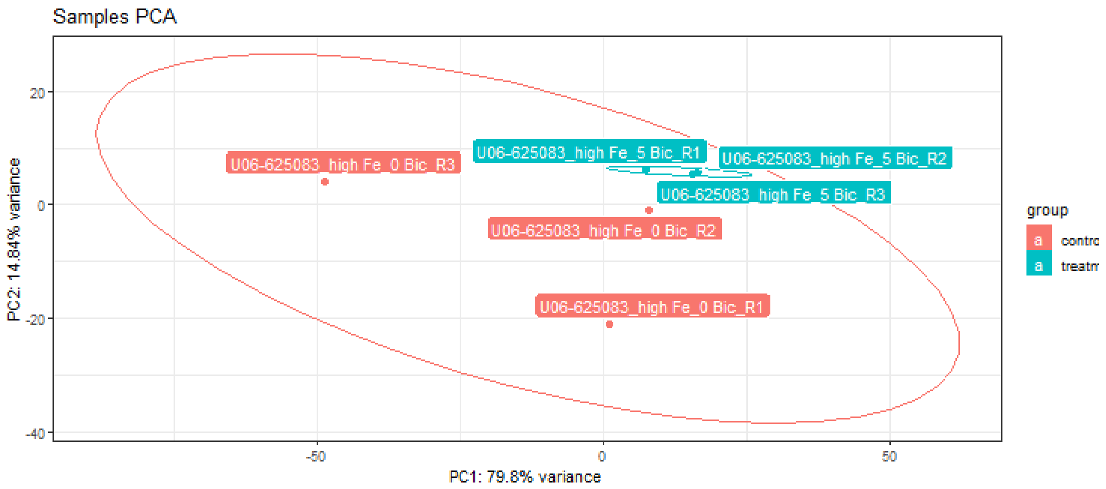

Ab13 - SRP108540_HighFe_5BIC_083

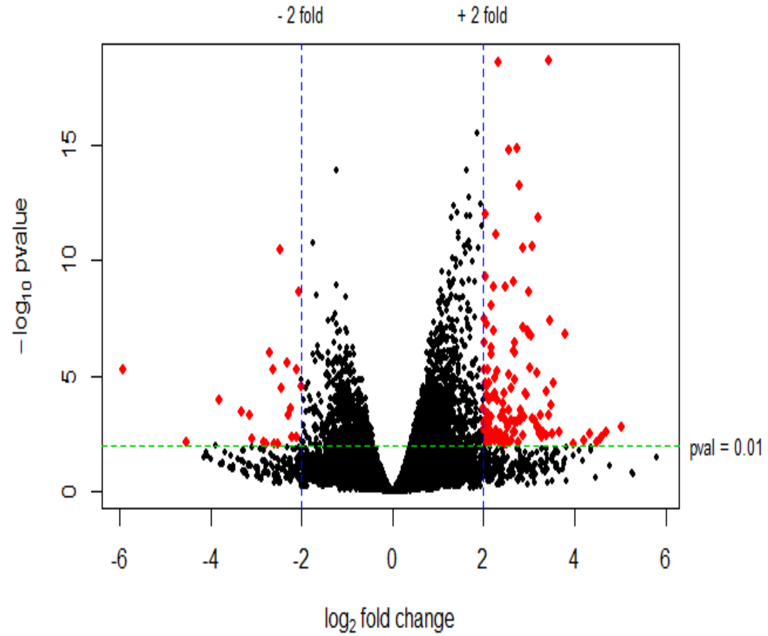

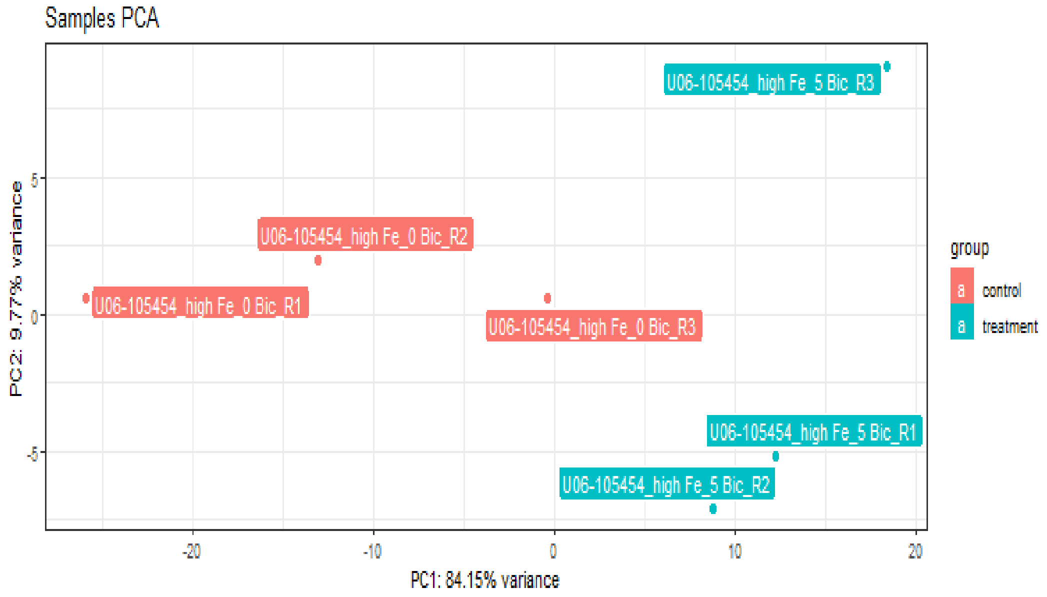

Ab13 - SRP108540_HighFe_5BIC_454

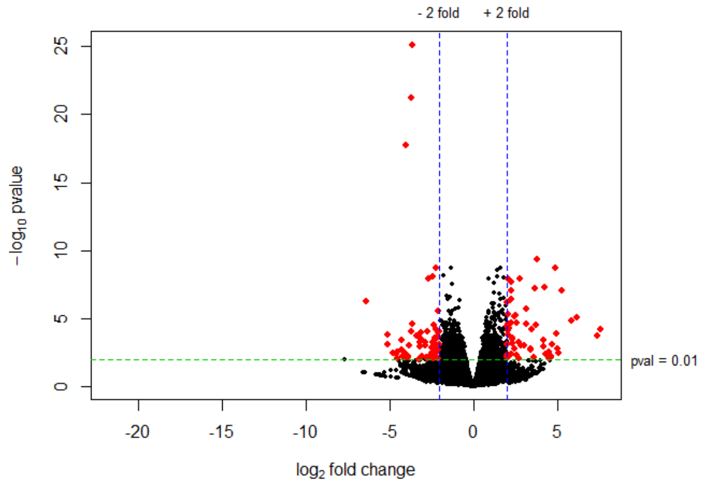

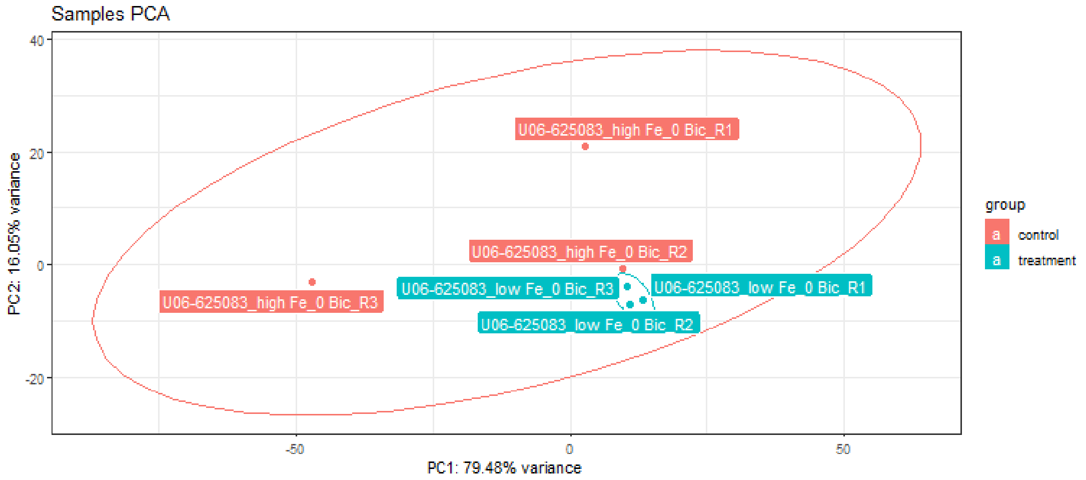

Ab13 - SRP108540_HighVsLOWFe_0BIC_083

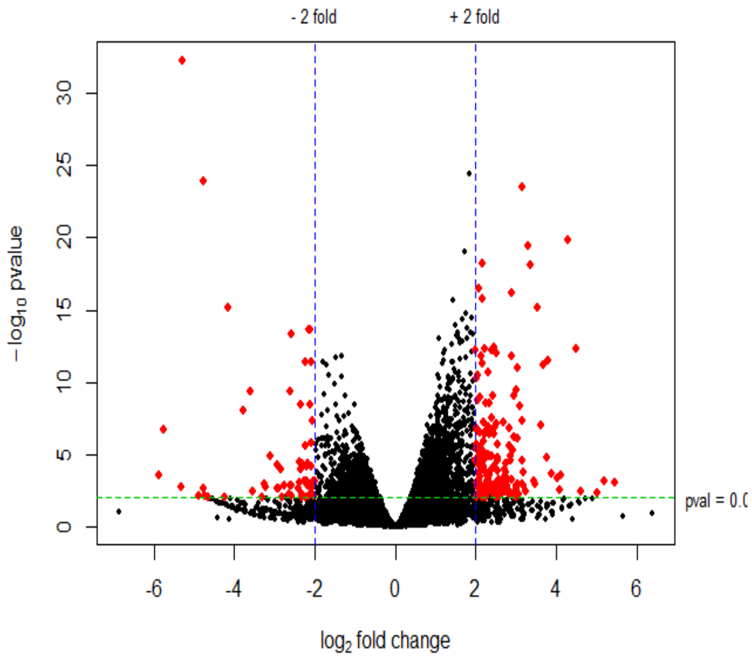

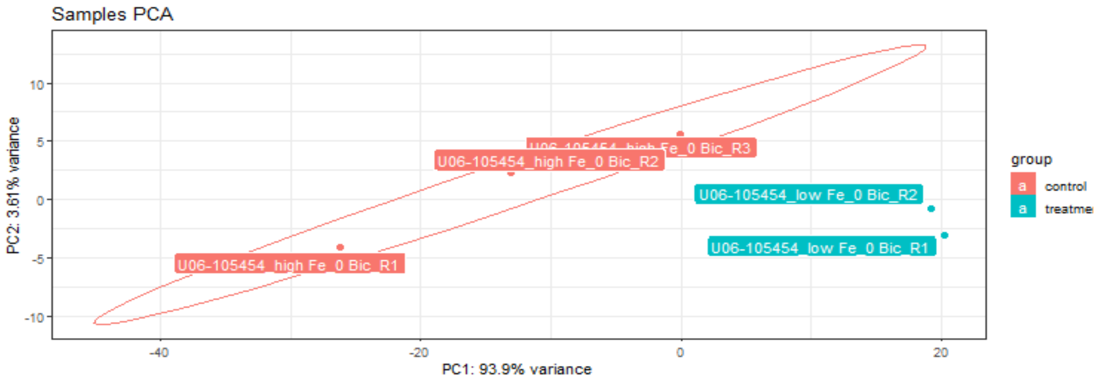

Ab13 - SRP108540_HighVsLOWFe_0BIC_454

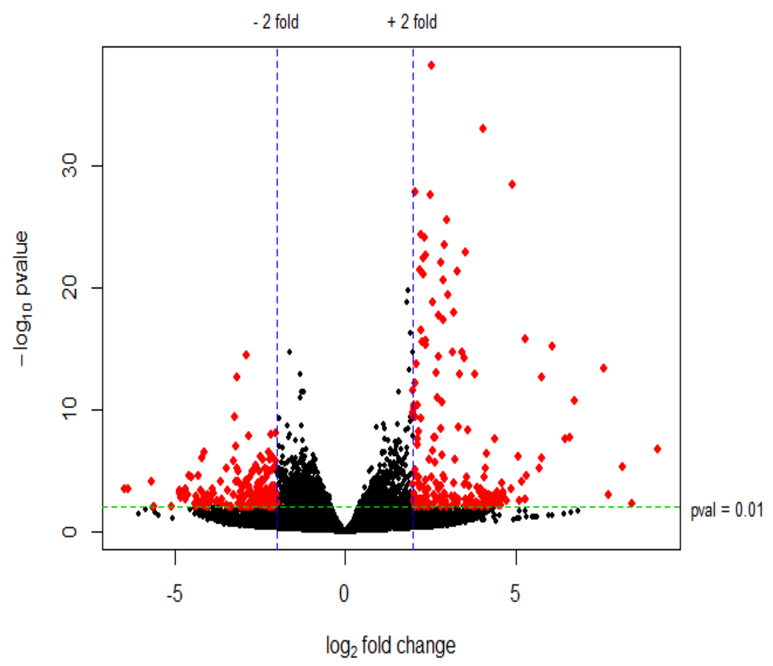

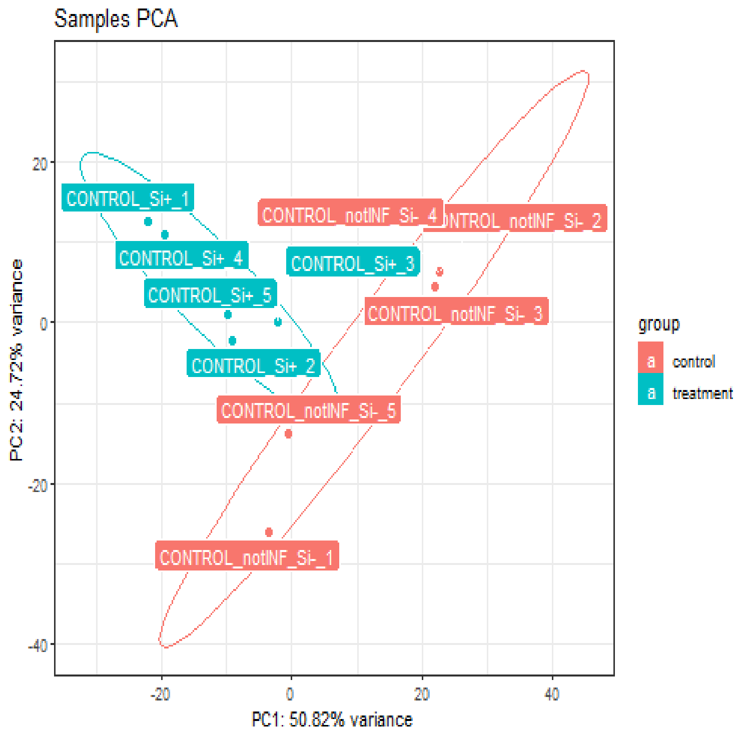

Bi1 - SRP155375_effect_Si_NoINF

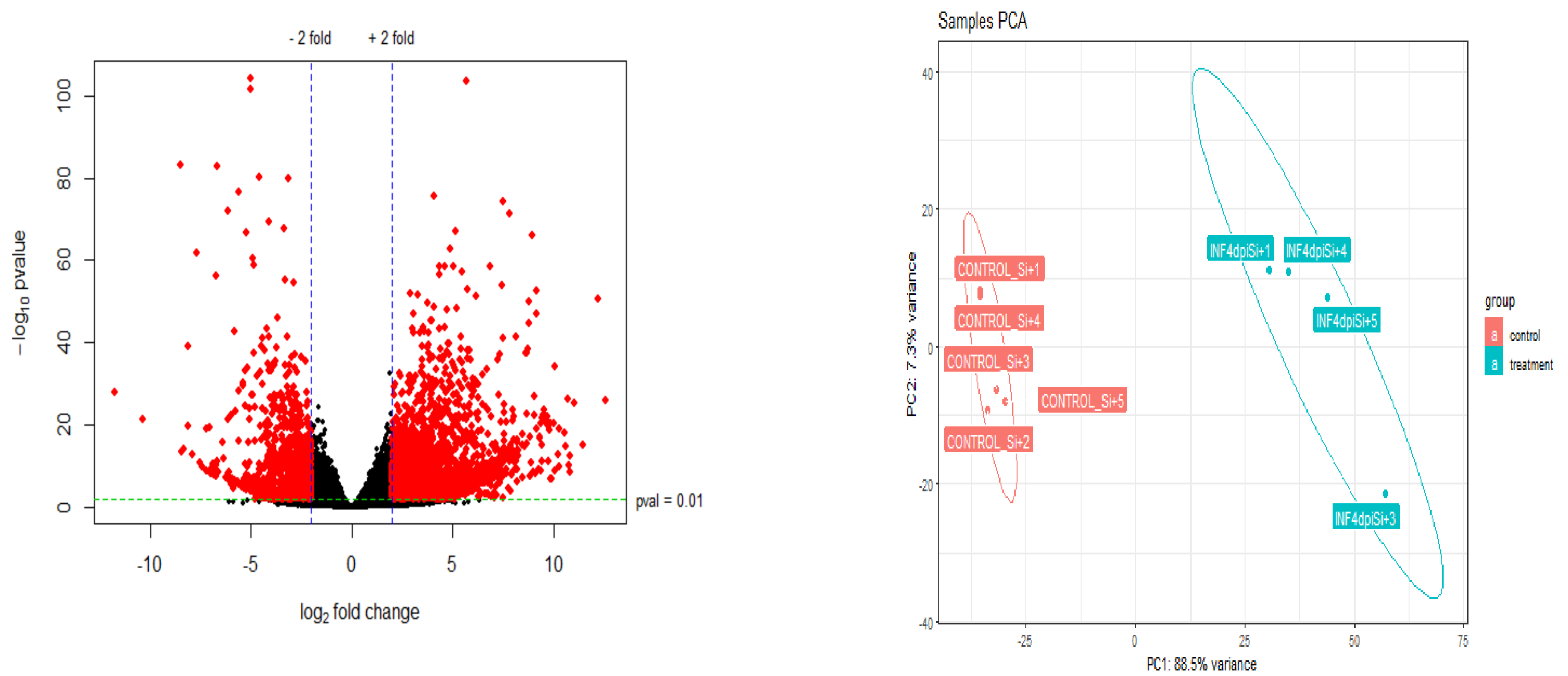

Bi1 - SRP155375_effect_Si+_INF4dpi

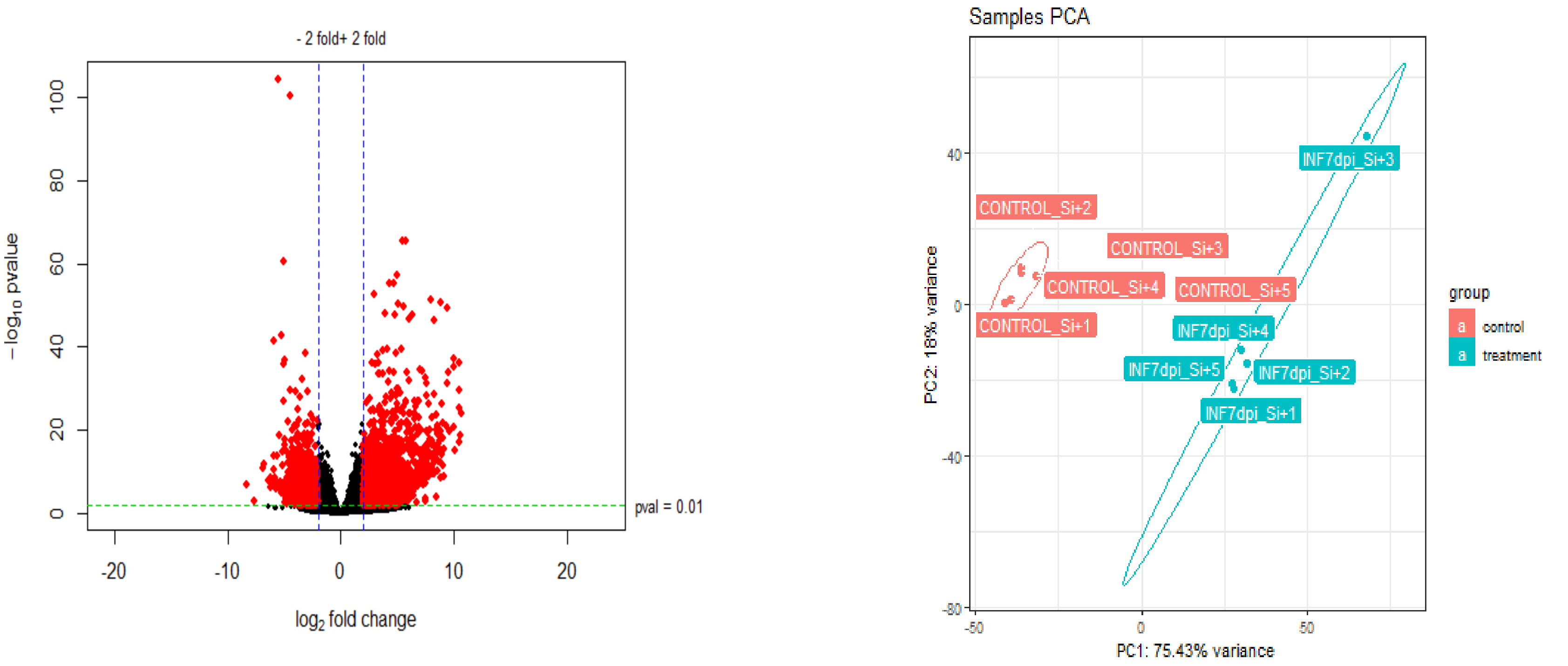

Bi1 - SRP155375_effect_Si+_INF7dpi

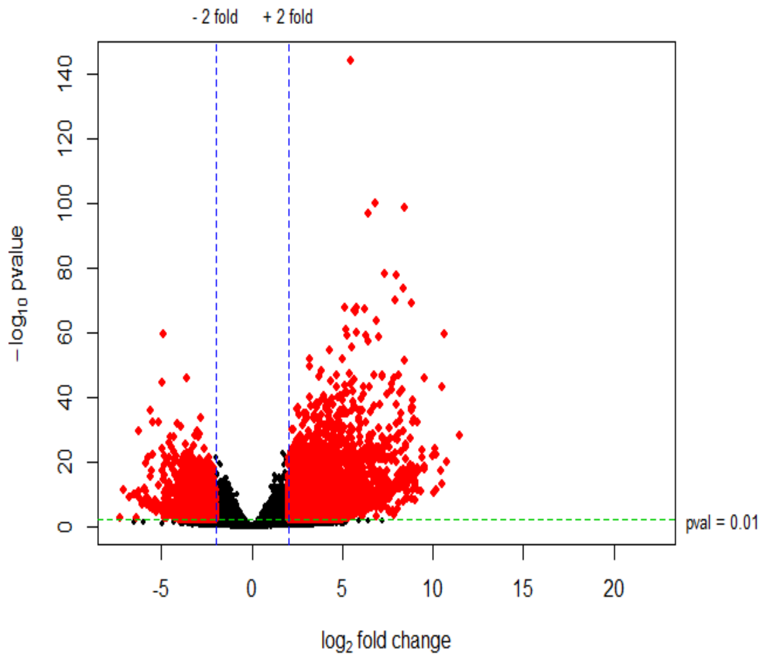

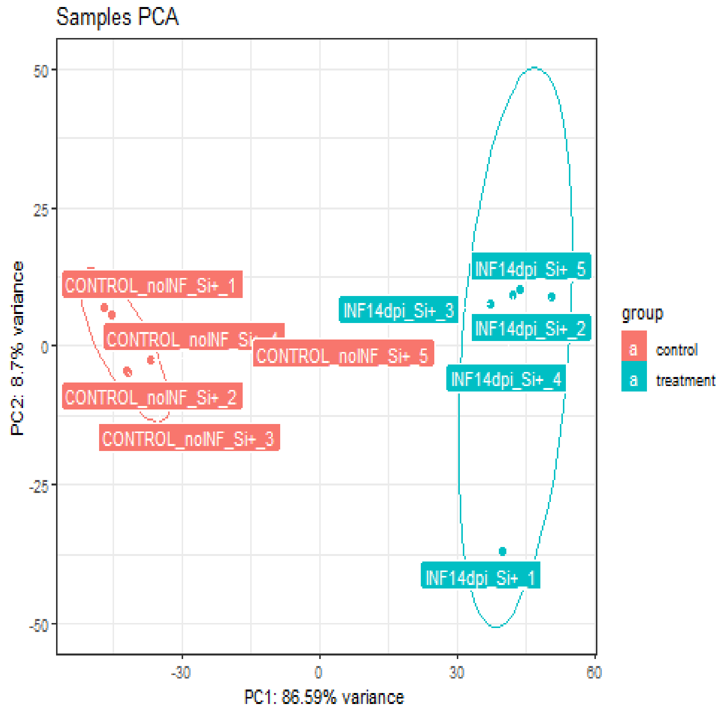

Bi1 - SRP155375_effect_Si+_INF14dpi

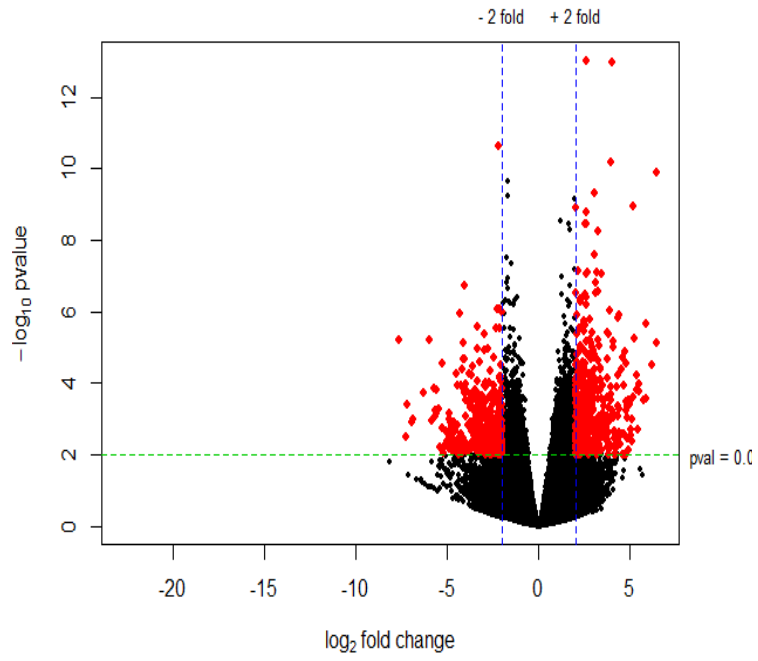

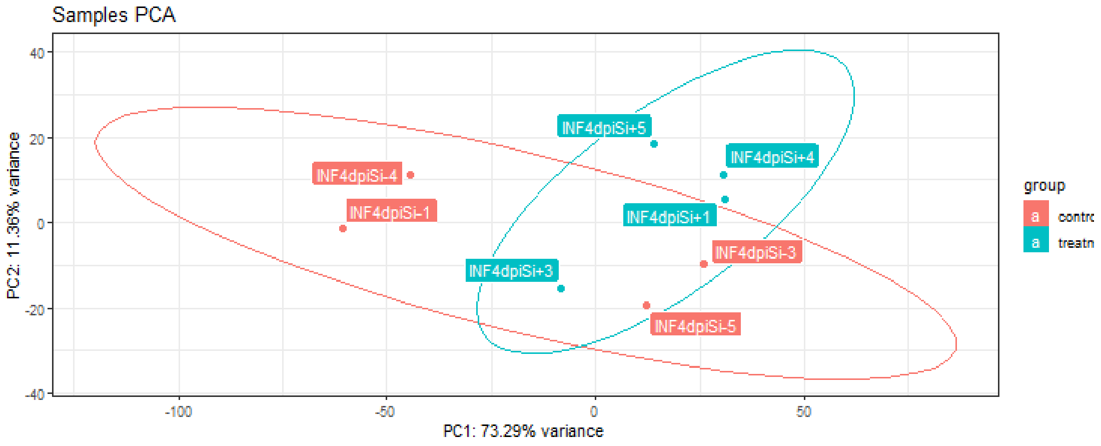

Bi1 - SRP155375_effect_Sieffect_INF4dpi

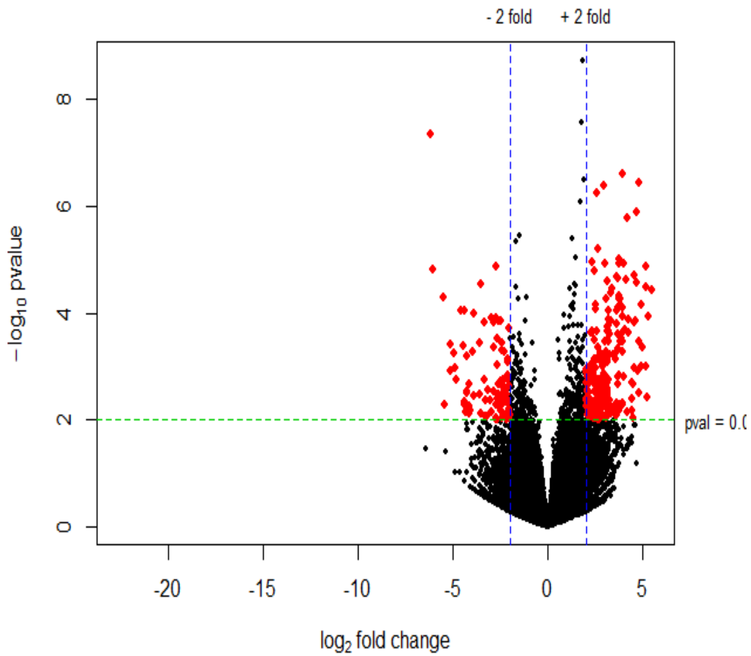

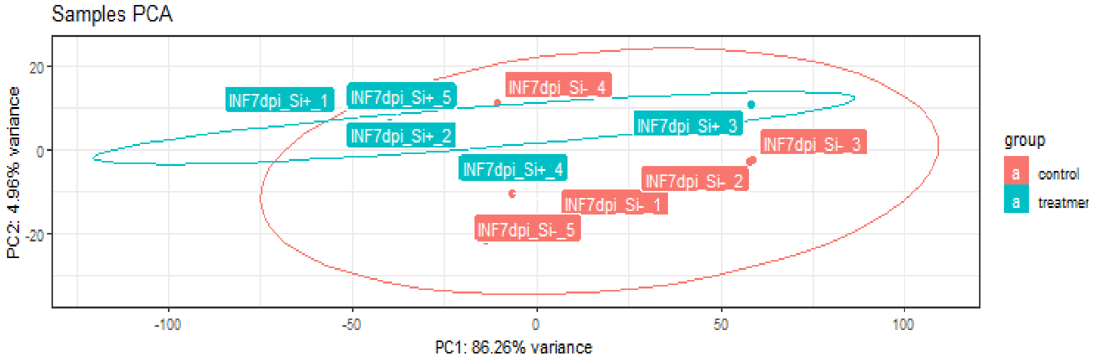

Bi1 - SRP155375_effect_Sieffect_INF7dpi

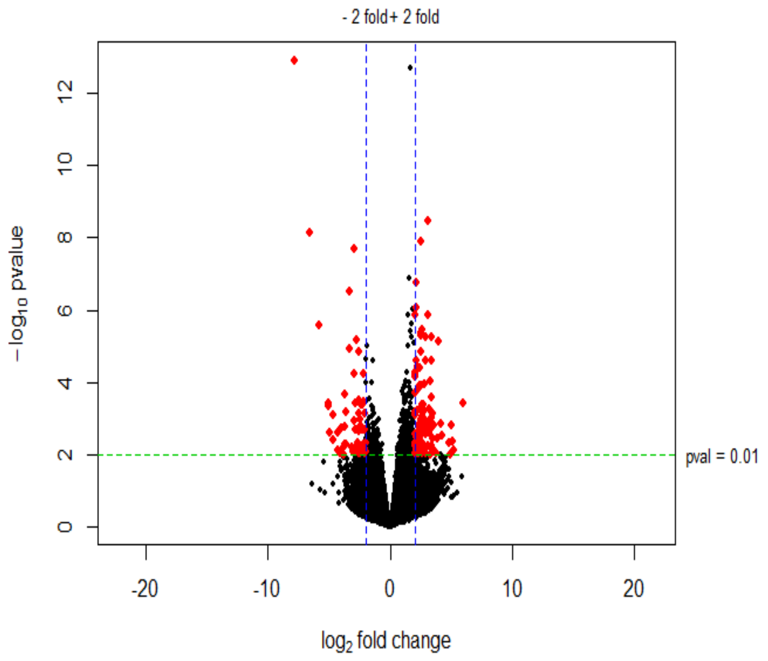

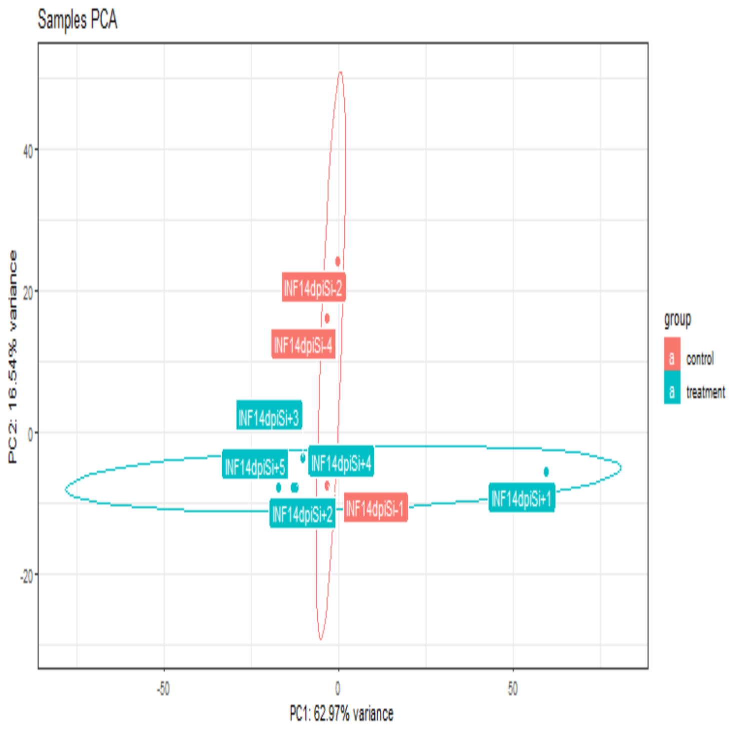

Bi1 - SRP155375_effect_Sieffect_INF14dpi

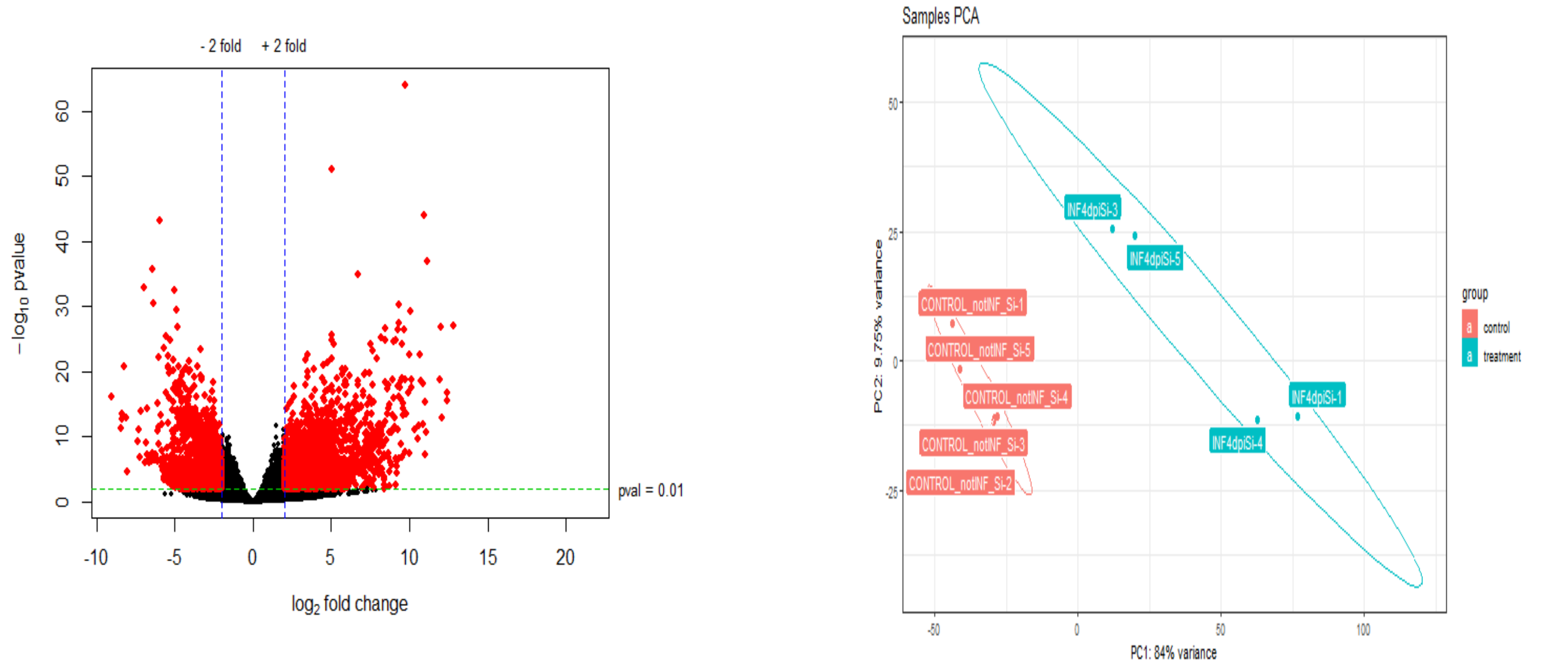

Bi1 - SRP155375_Si-_INF4dpi

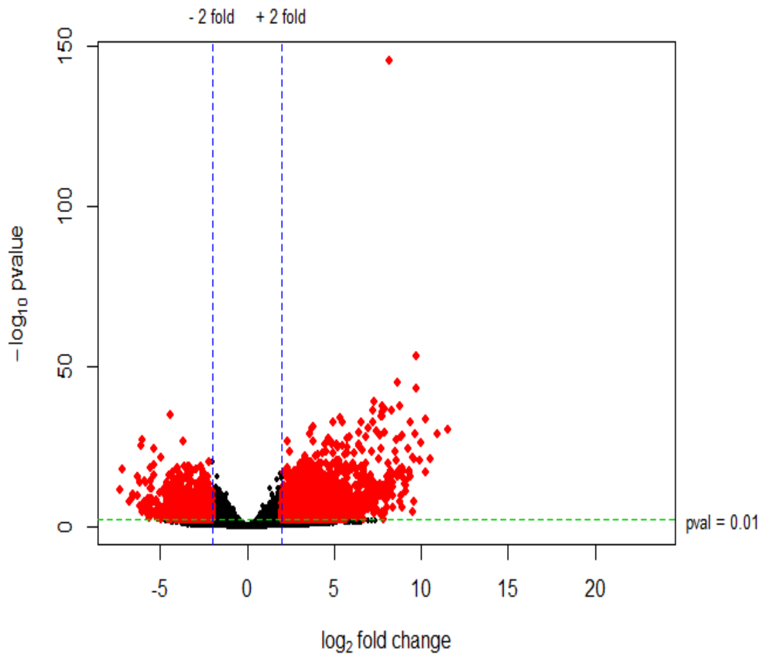

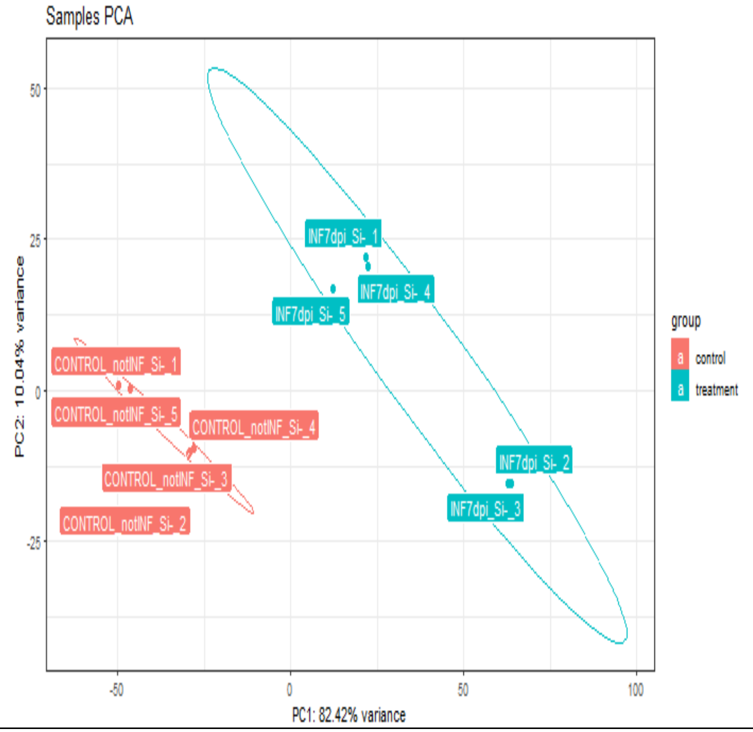

Bi1 - SRP155375_Si-_INF7dpi

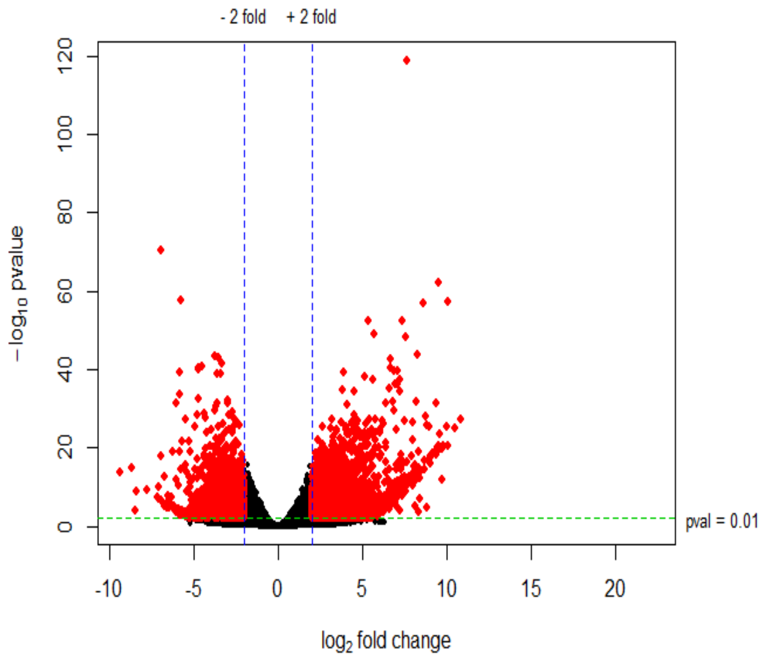

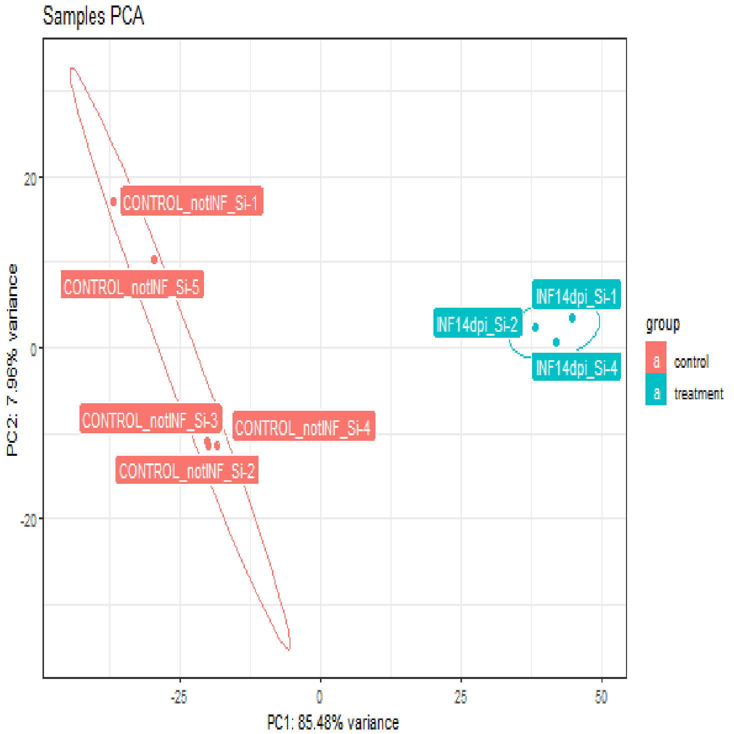

Bi1 - SRP155375_Si-_INF14dpi

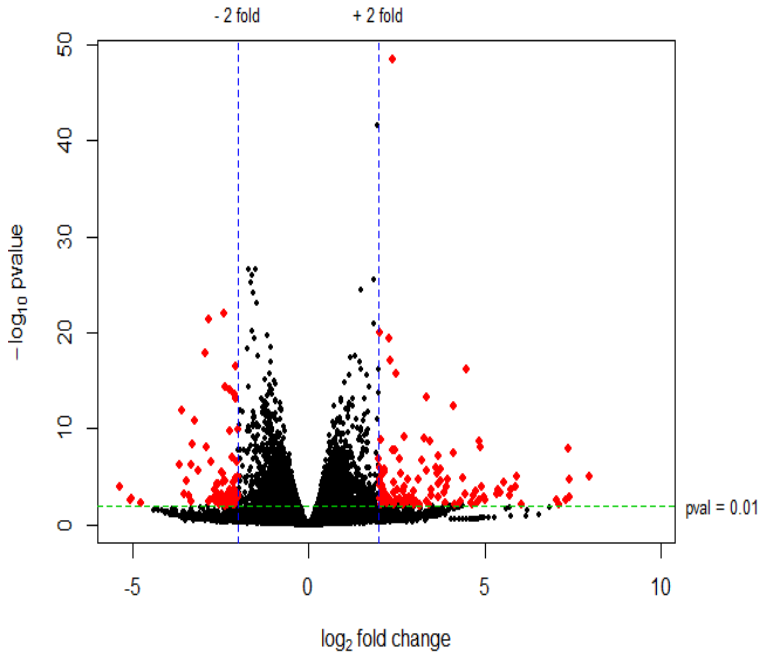

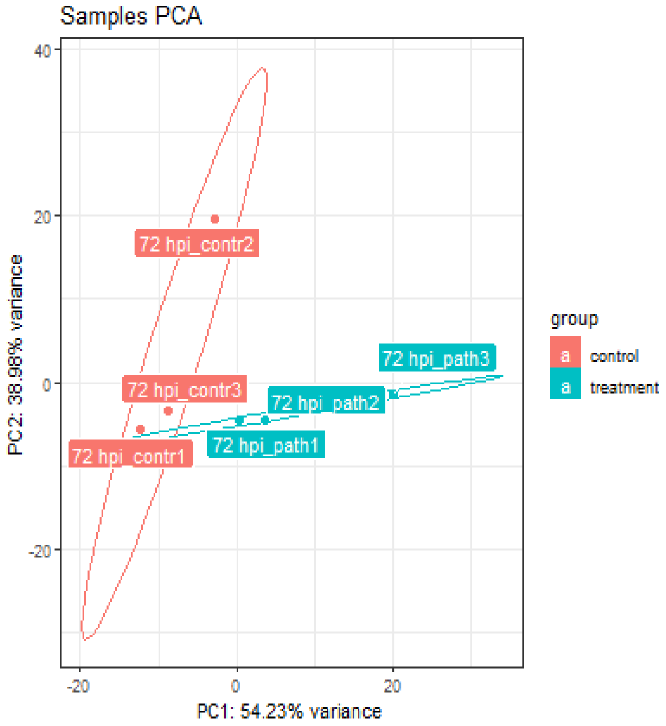

Bi2 - SRP056137_FO_path72hpi

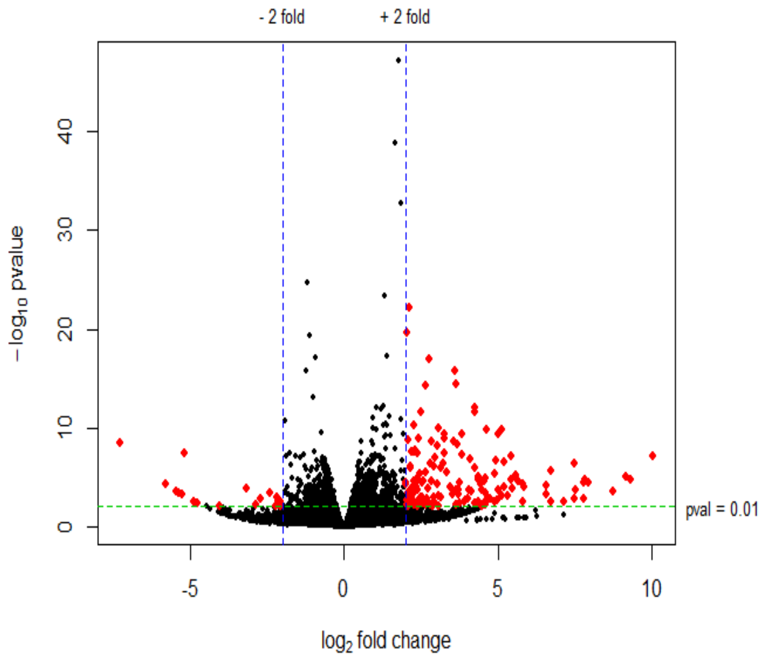

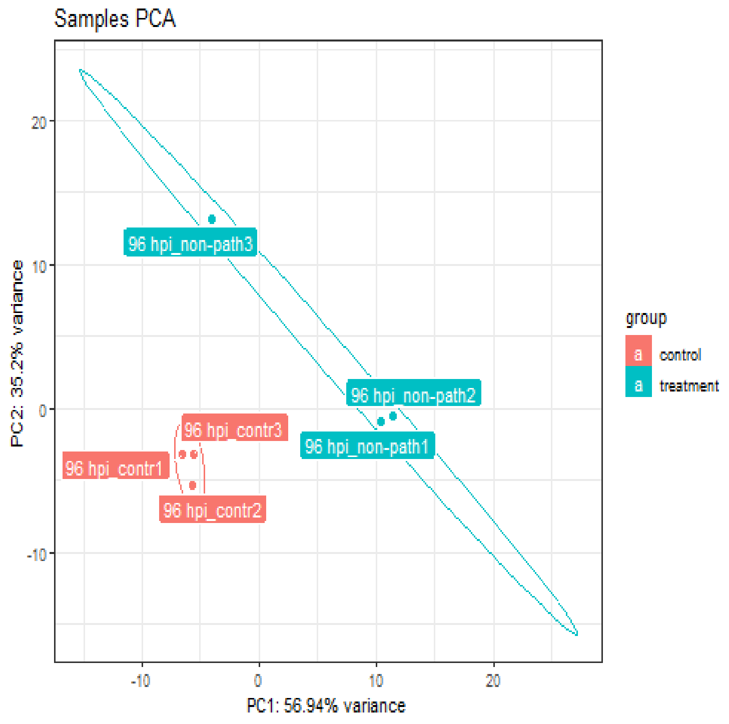

Bi2 - SRP056137_FO_path96hpi

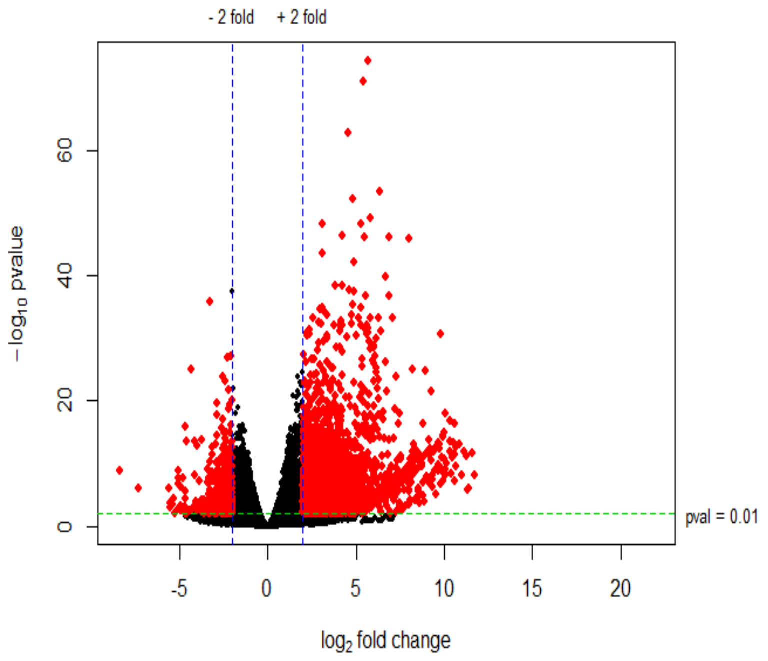

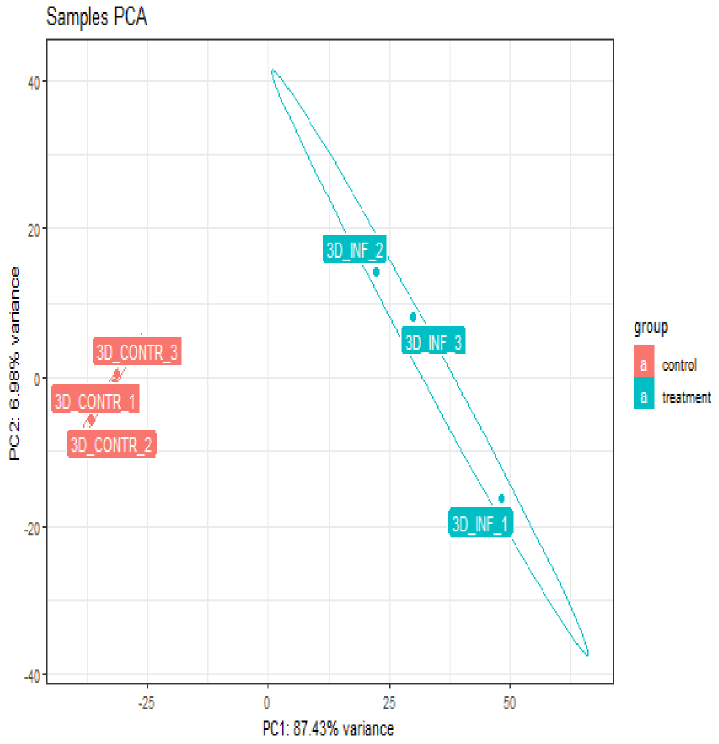

Bi3 - SRP091708_Rotylenchus_NEMATOD_3DAI

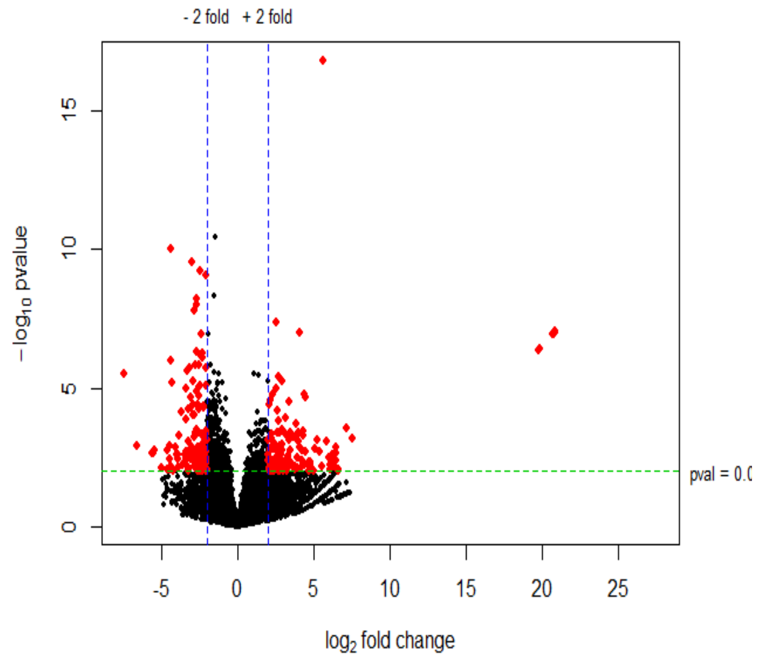

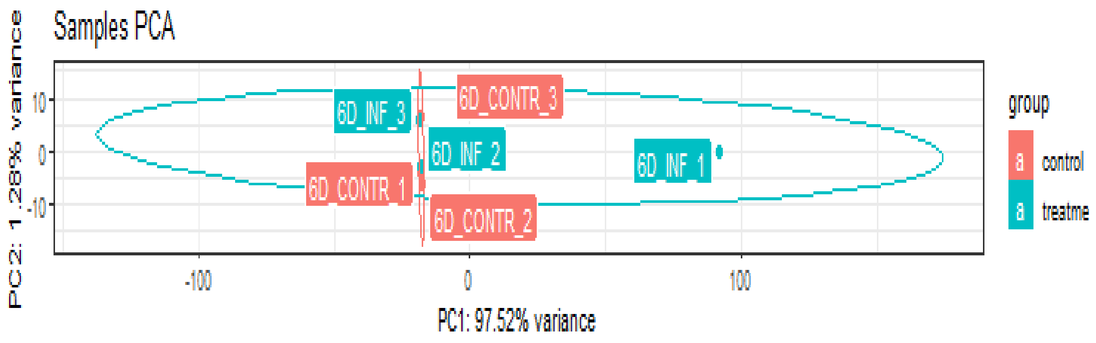

Bi3 - SRP091708__Rotylenchus_NEMATOD_6DAI

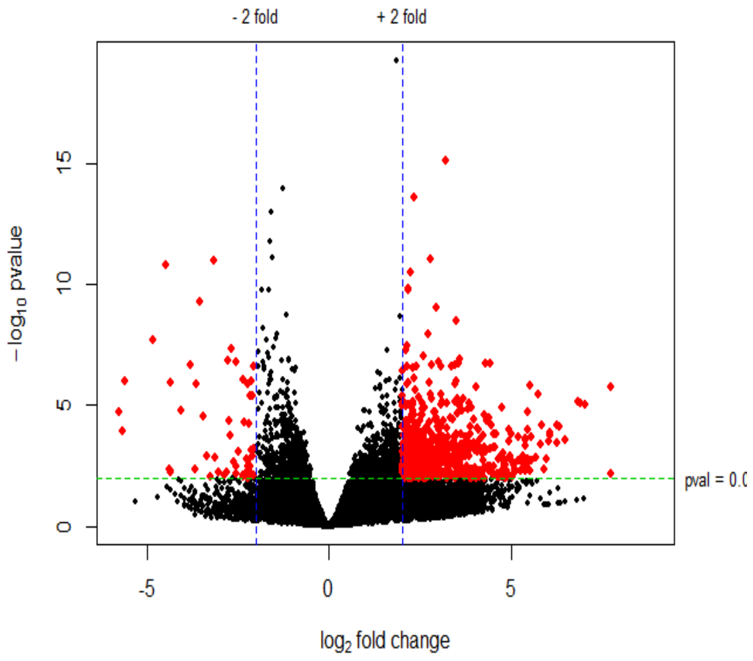

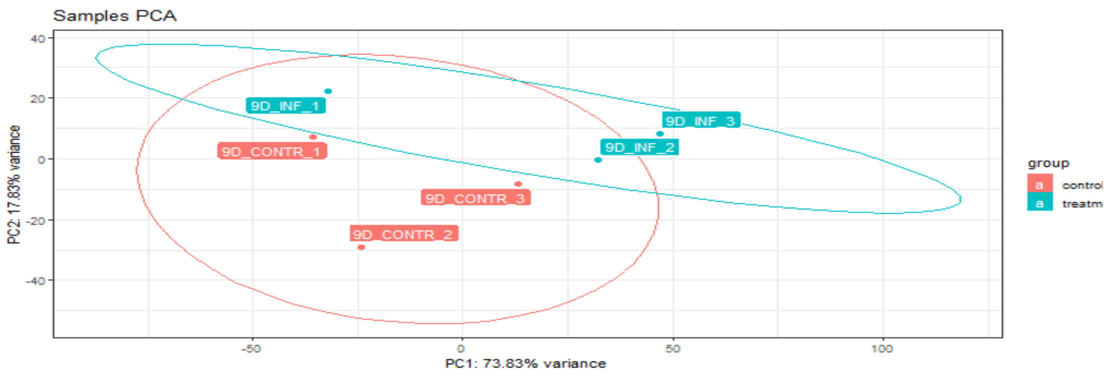

Bi3 - SRP091708__Rotylenchus_NEMATOD_9DAI

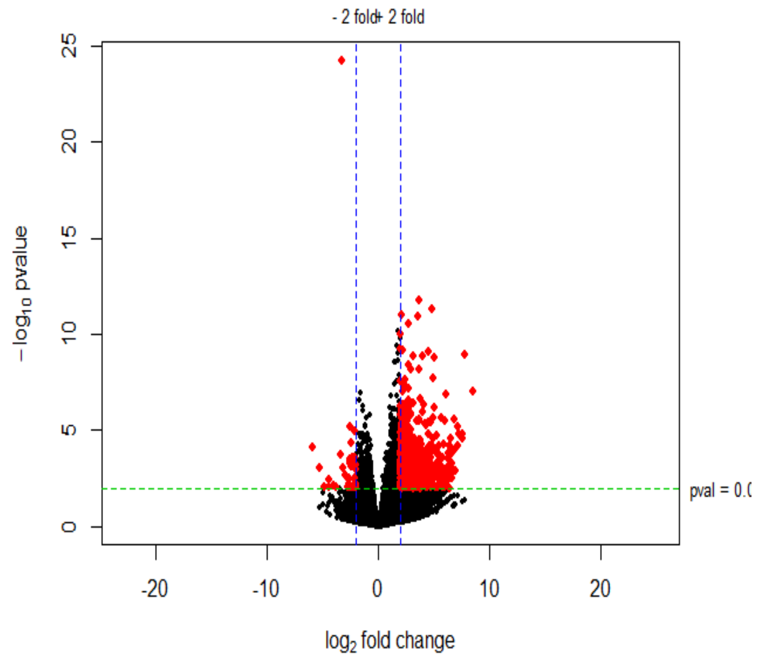

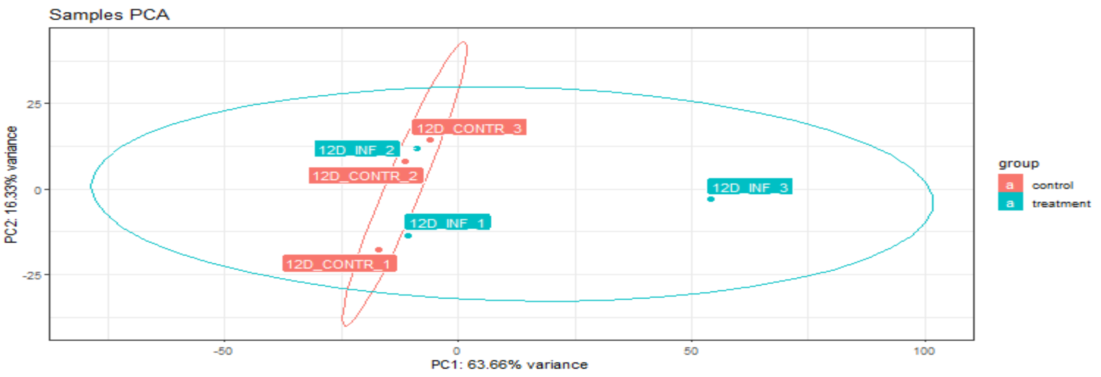

Bi3 - SRP091708__Rotylenchus_NEMATOD_12DAI

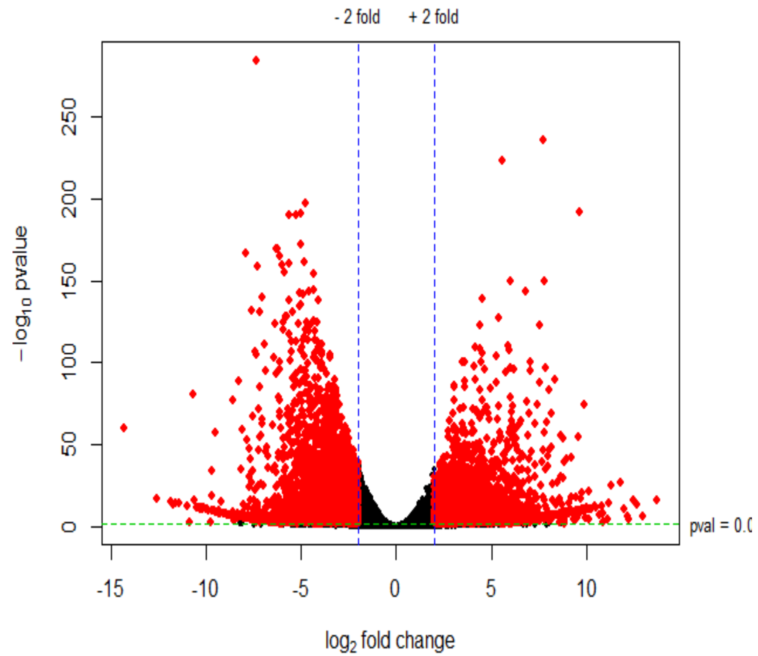

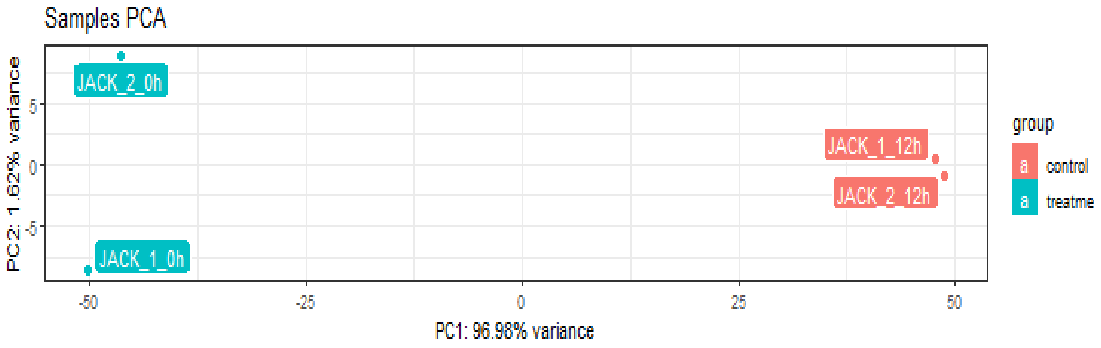

Bi4 - SRP126743_SMV_JACK12h

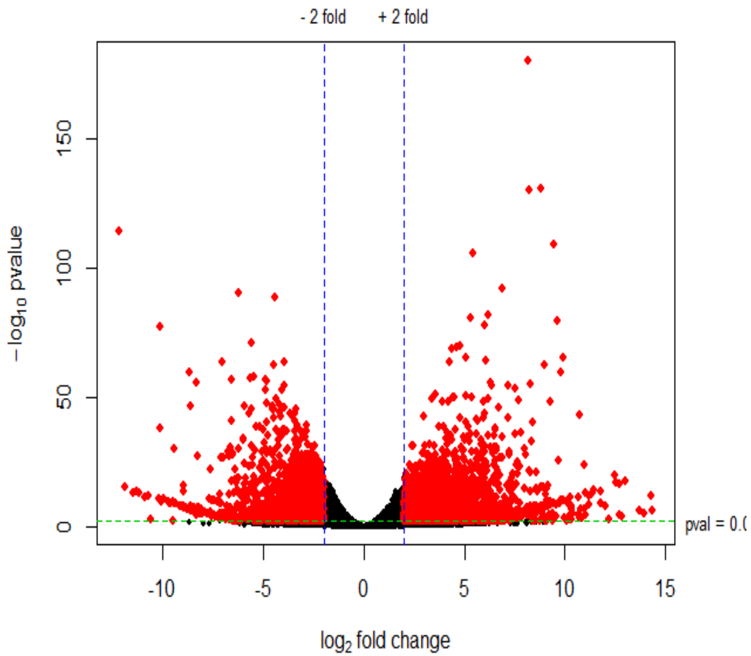

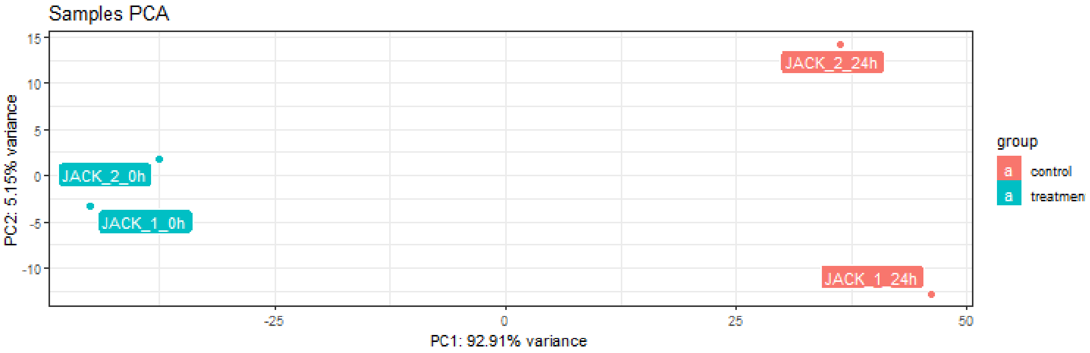

Bi4 - SRP126743_SMV_JACK24h

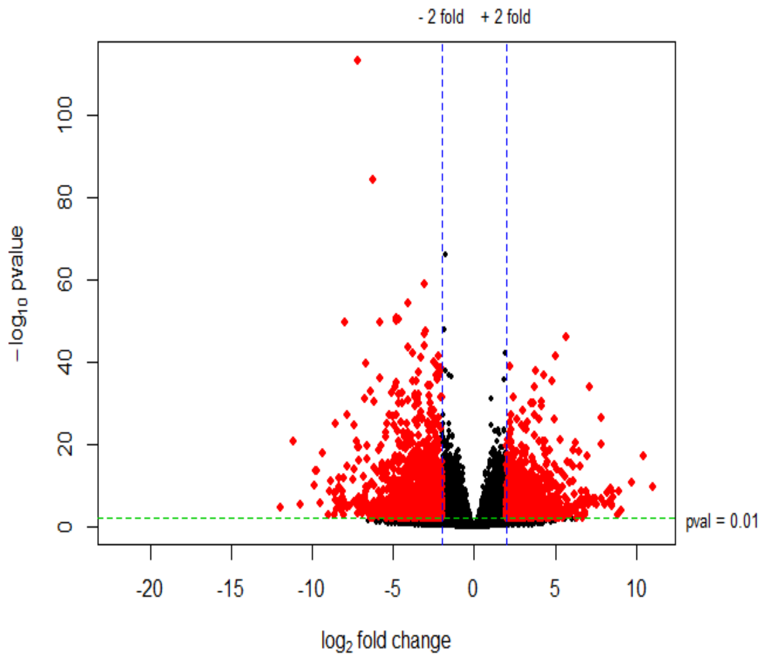

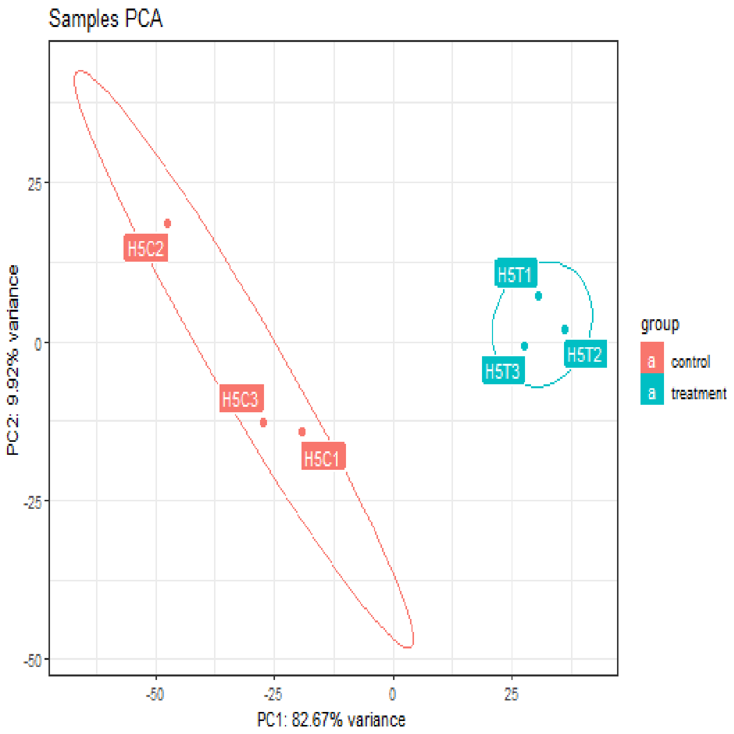

Bi5 - SRP135932_Huipizihi_nematode_5DAI

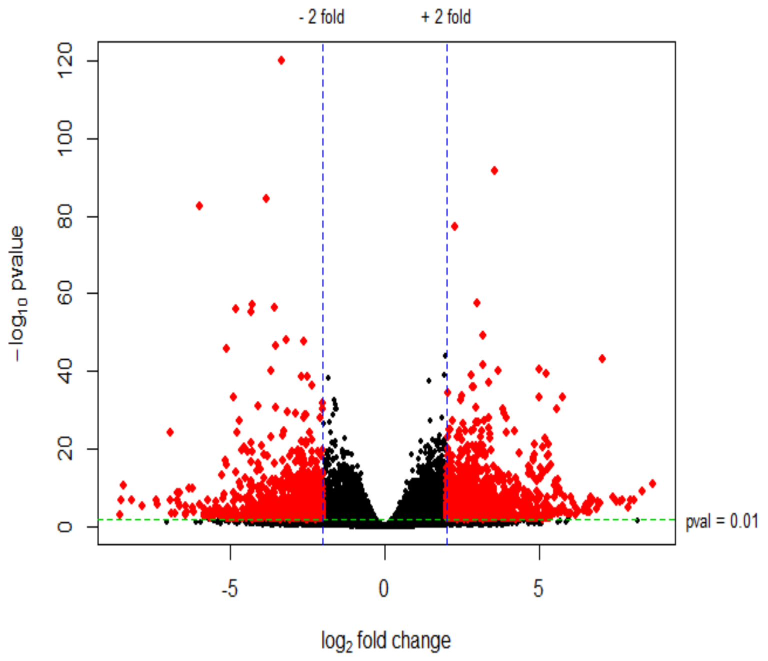

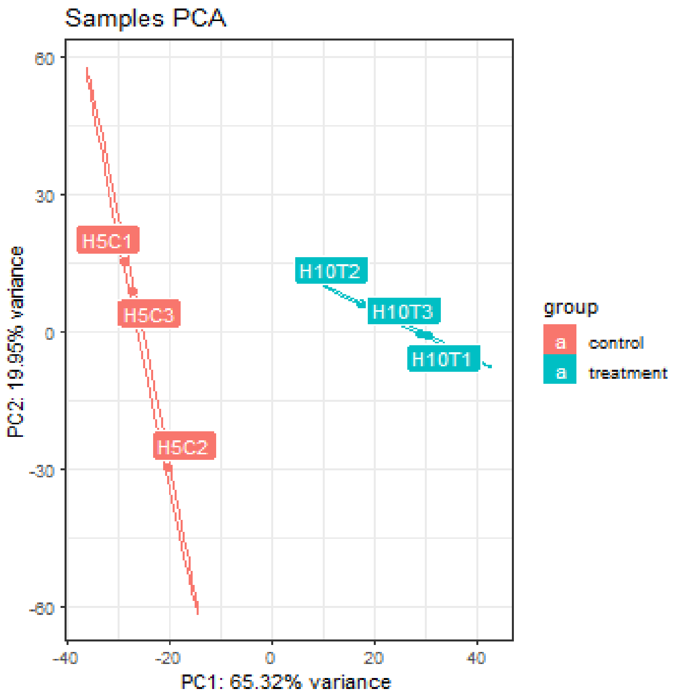

Bi5 - SRP135932_Huipizihi_nematode_10DAI

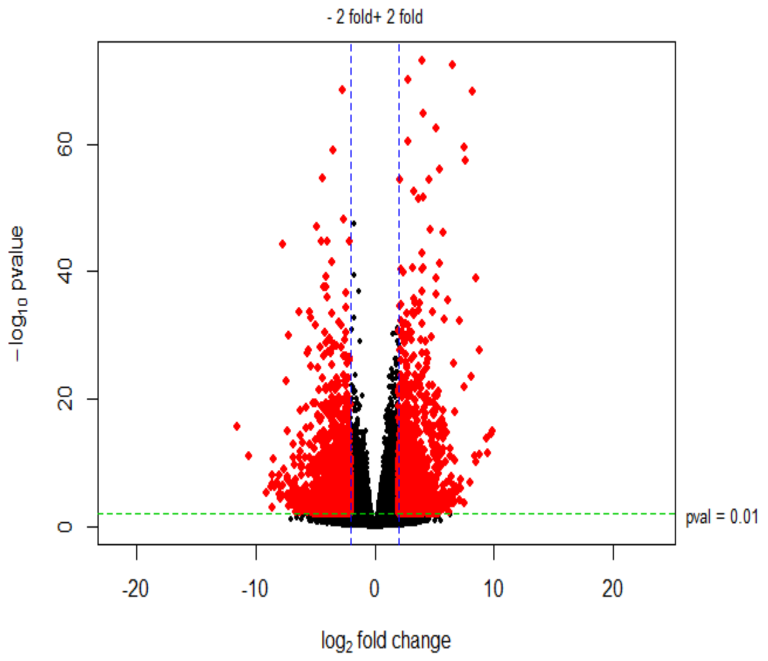

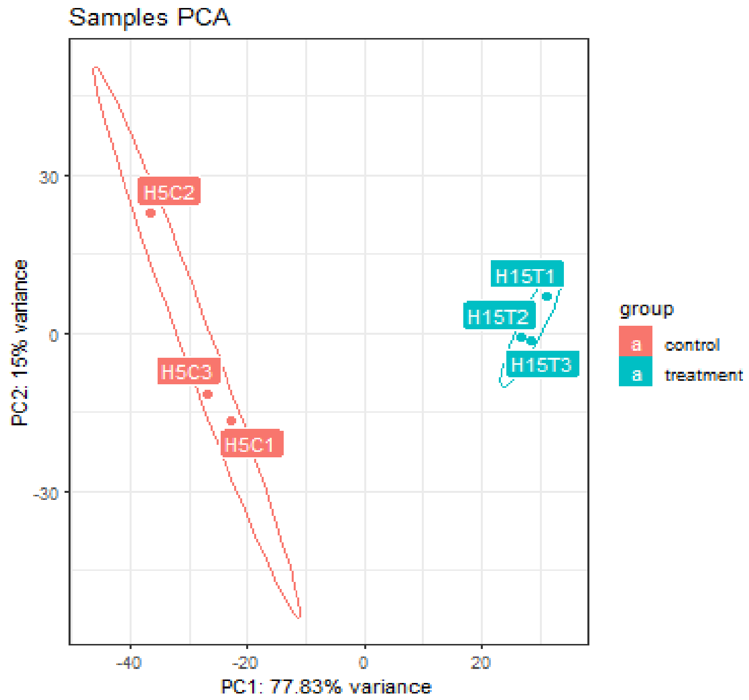

Bi5 - SRP135932_Huipizihi_nematode_15DAI

(**A**)

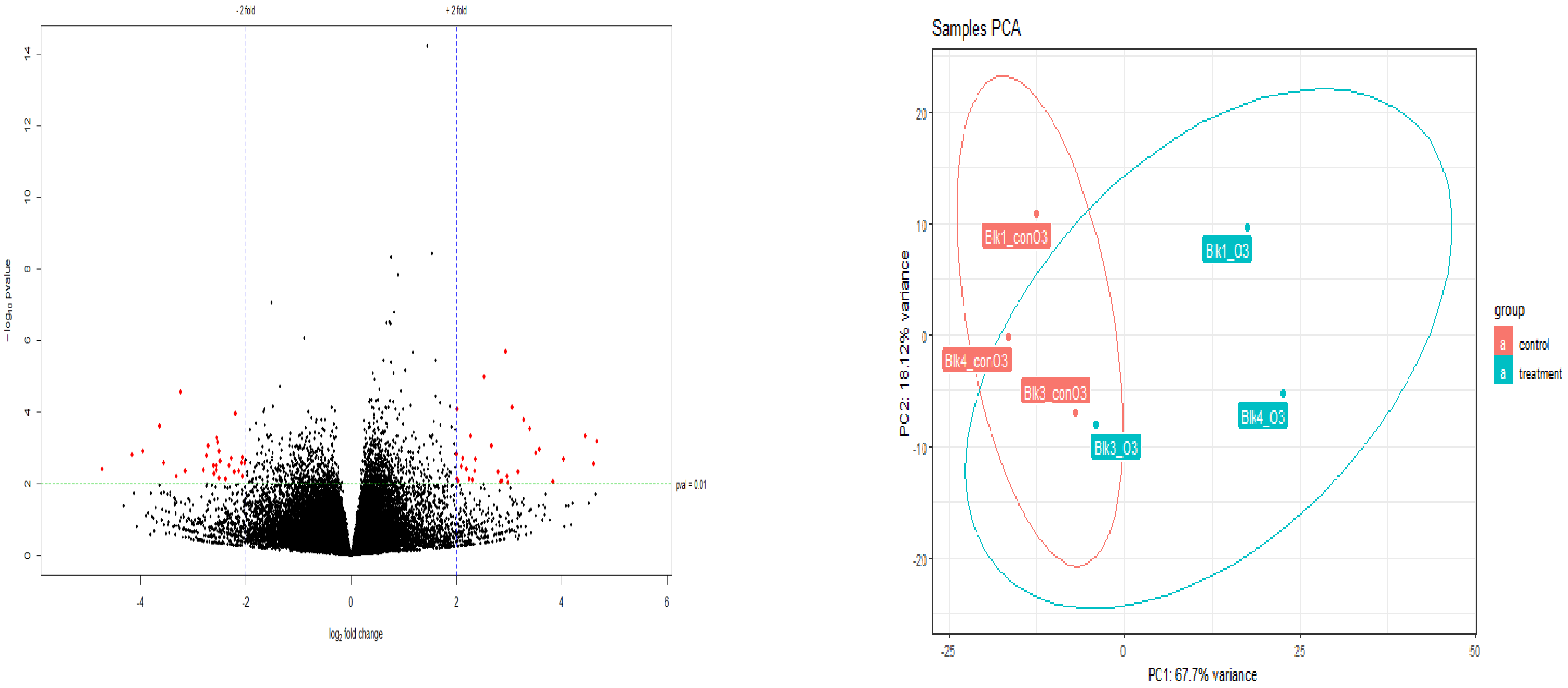

Ab2 - SRP024277 - Ozone (100 nl L^{-1}) - Pioneer 93B15 - Seed Coat (mature R8)

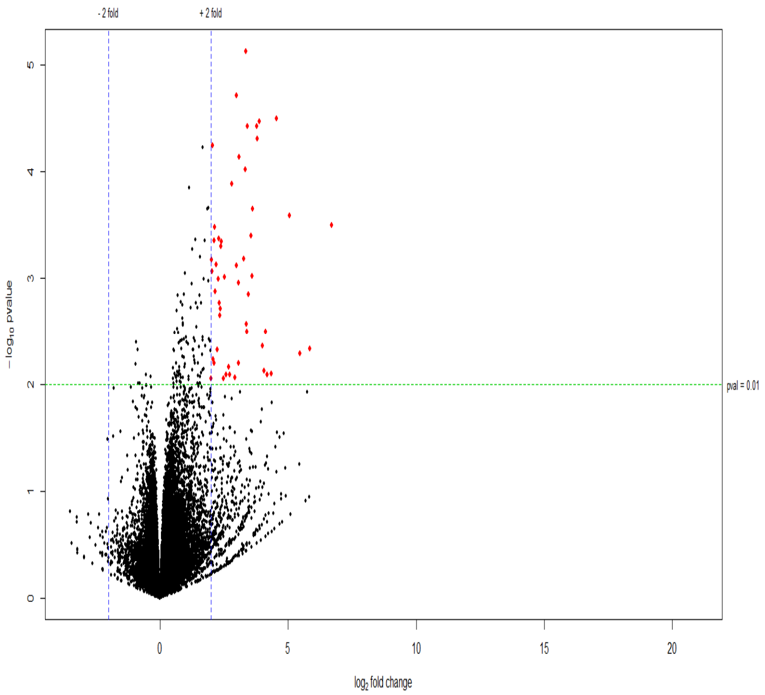

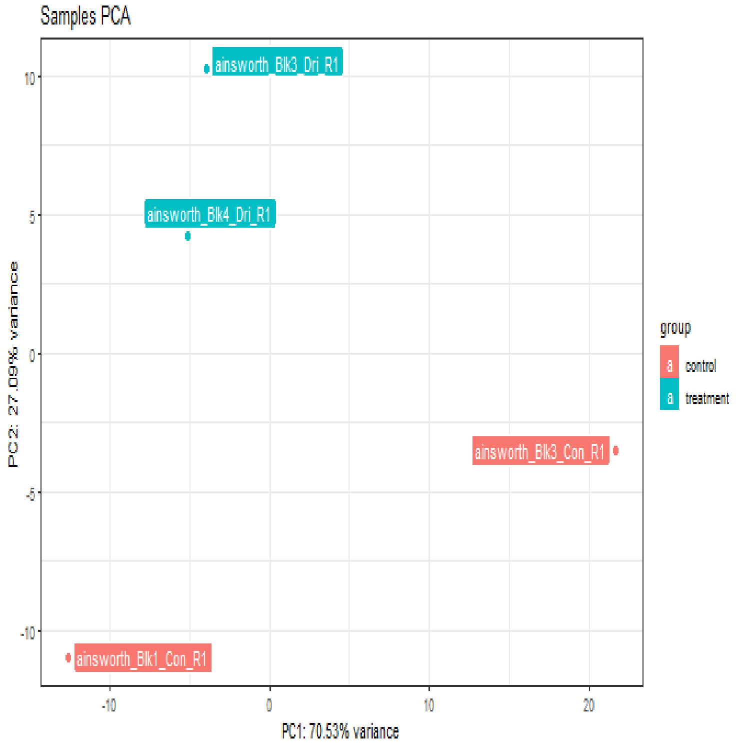

Ab2 - SRP024277 - Drought (interception of nighttime rainfall) - Pioneer 93B15 - Seed Coat (mature R8)

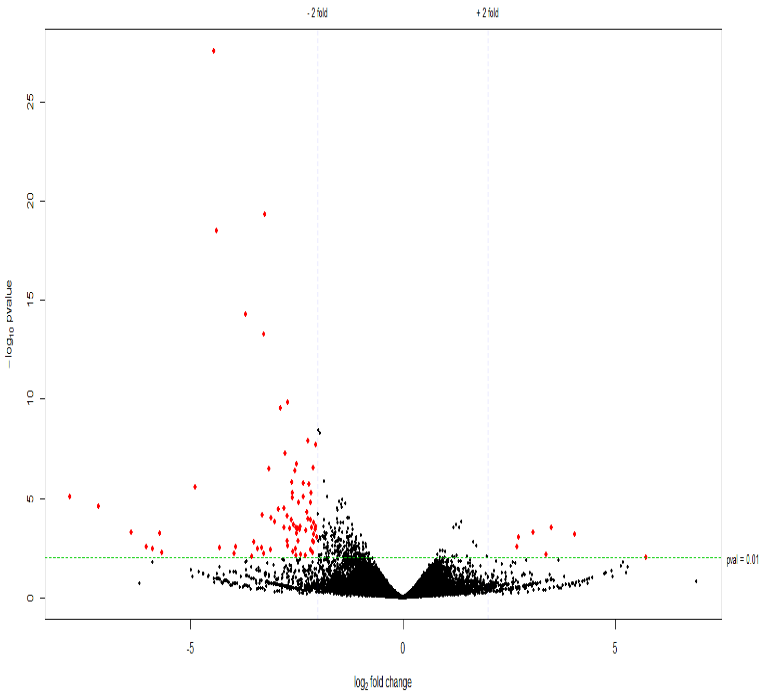

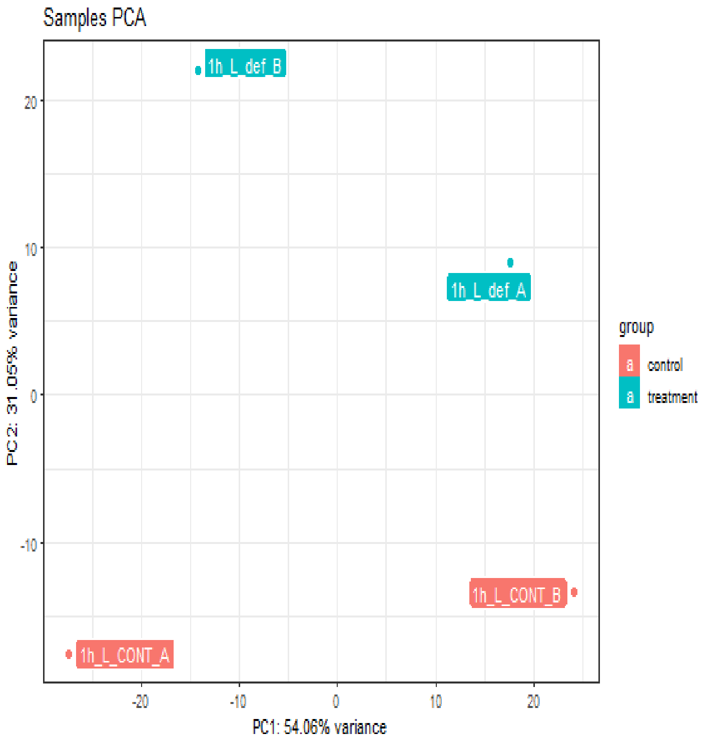

Ab3 - SRP031889 - Fe deficiency (100 µM vs. 50 µM) – Clark – Leaf 1h

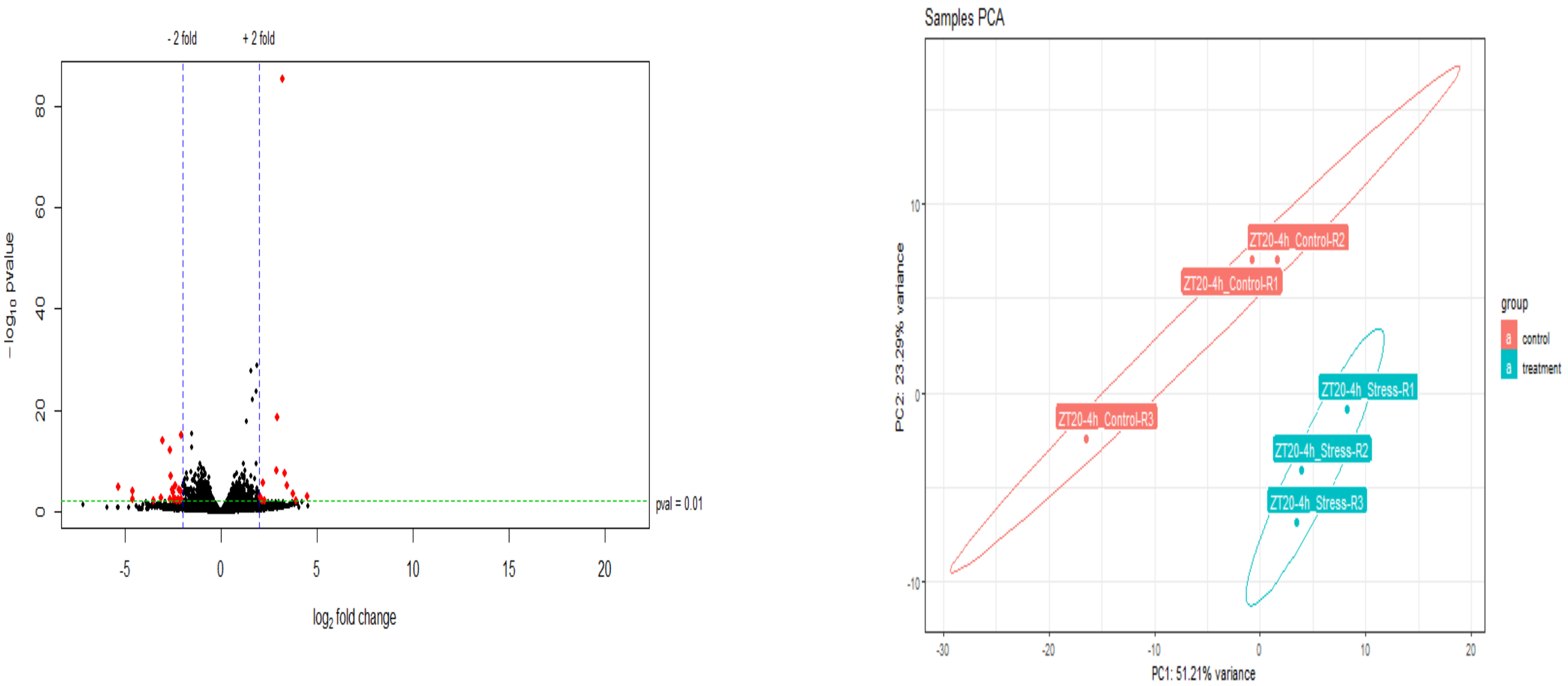

Ab9 - SRP058975_WDef_4h

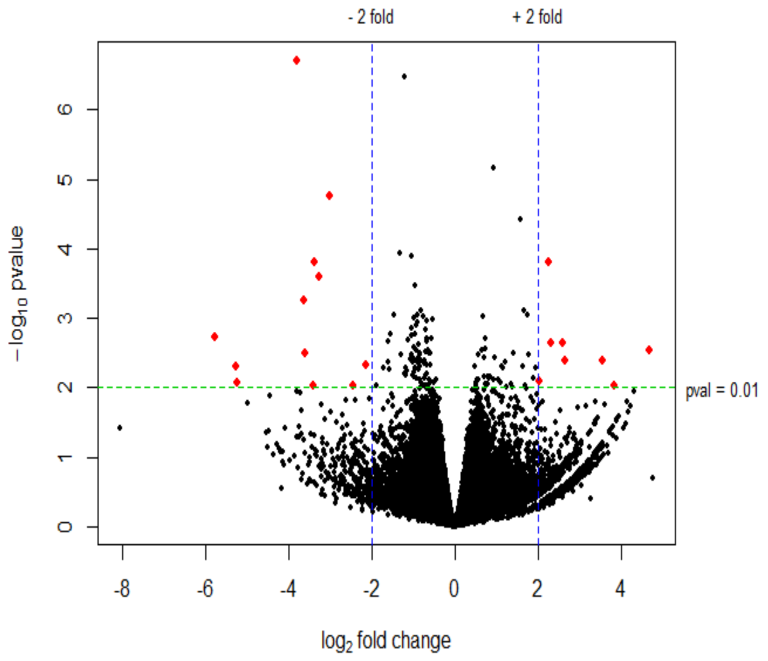

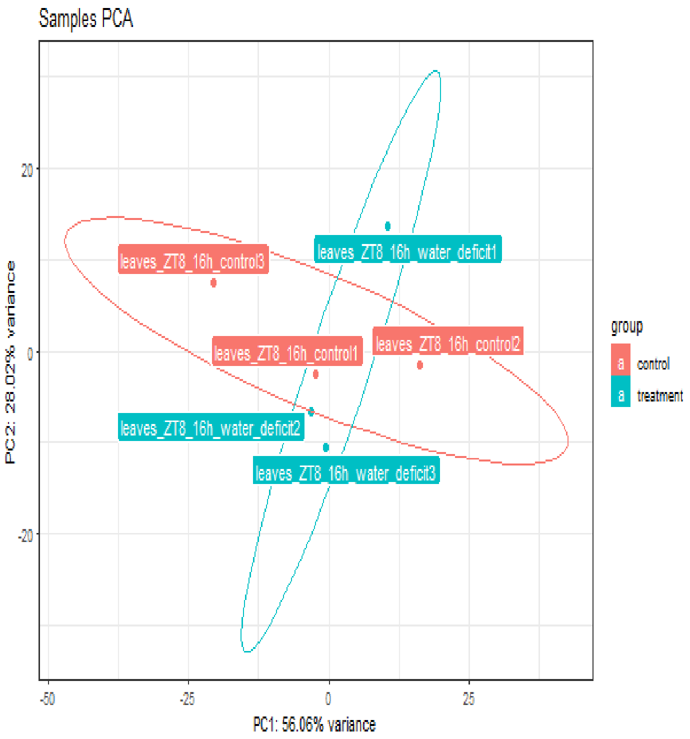

Ab9 - SRP058975_WDef_16h

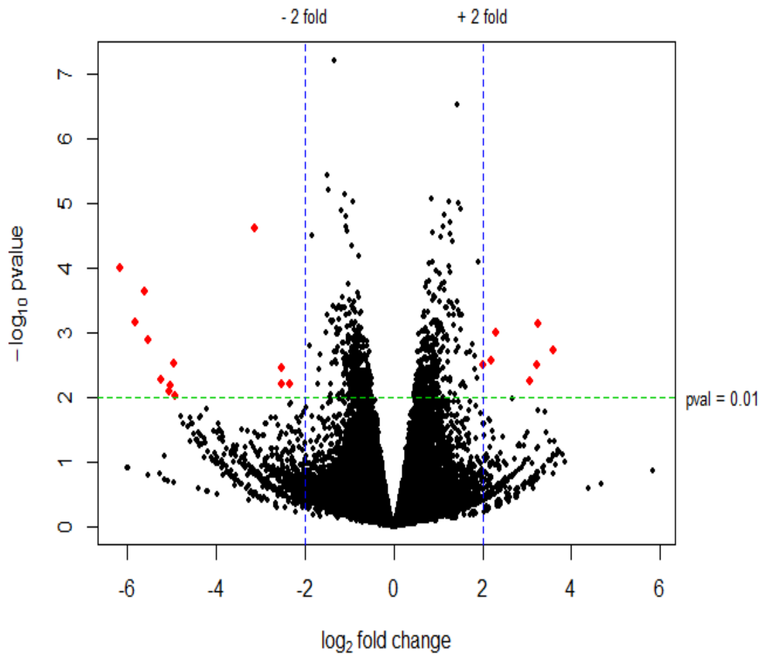

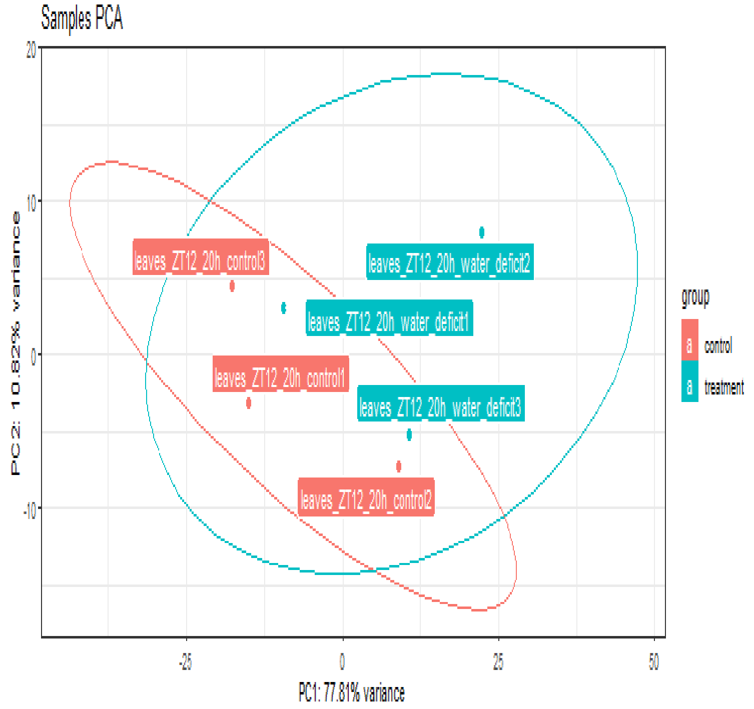

Ab9 - SRP058975_WDef_20h

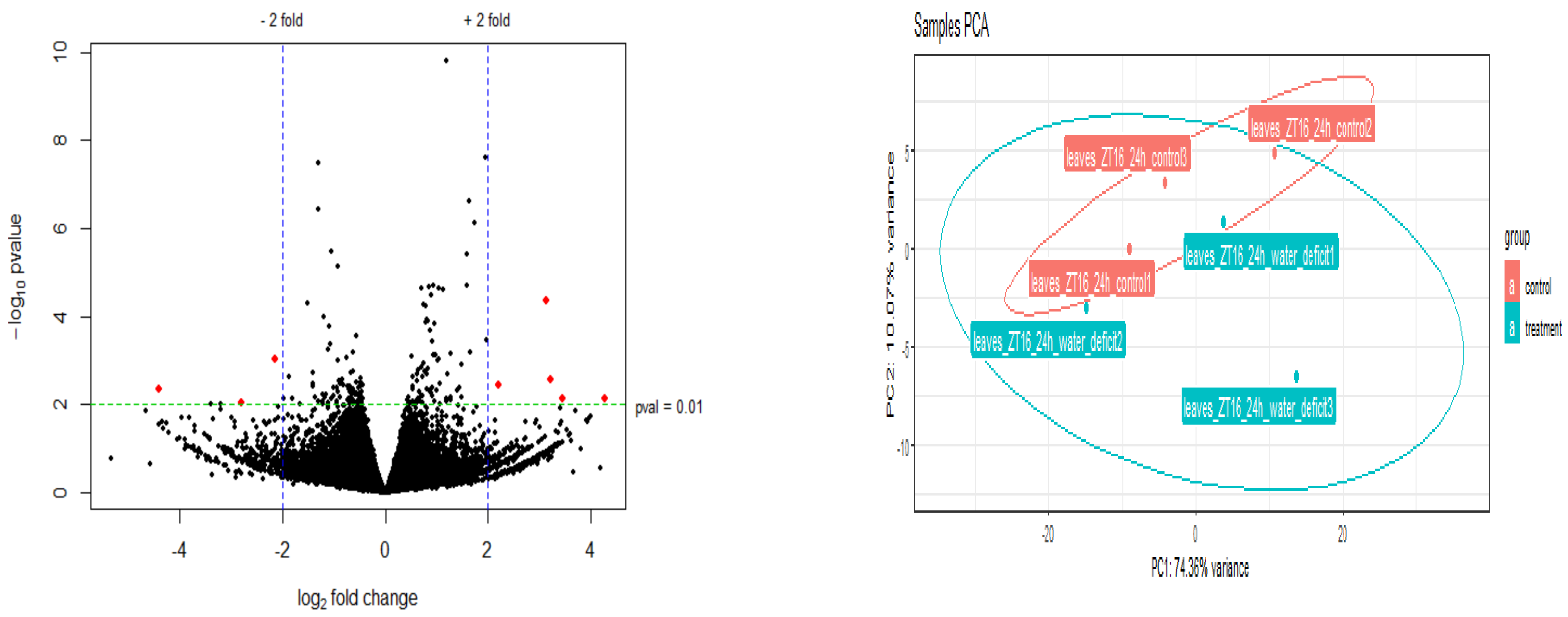

Ab9 - SRP058975_WDef_24h

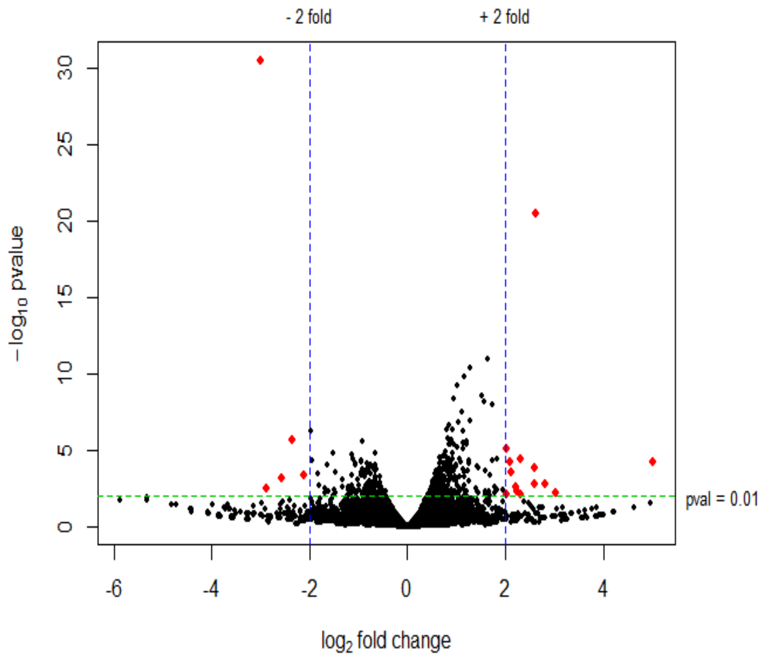

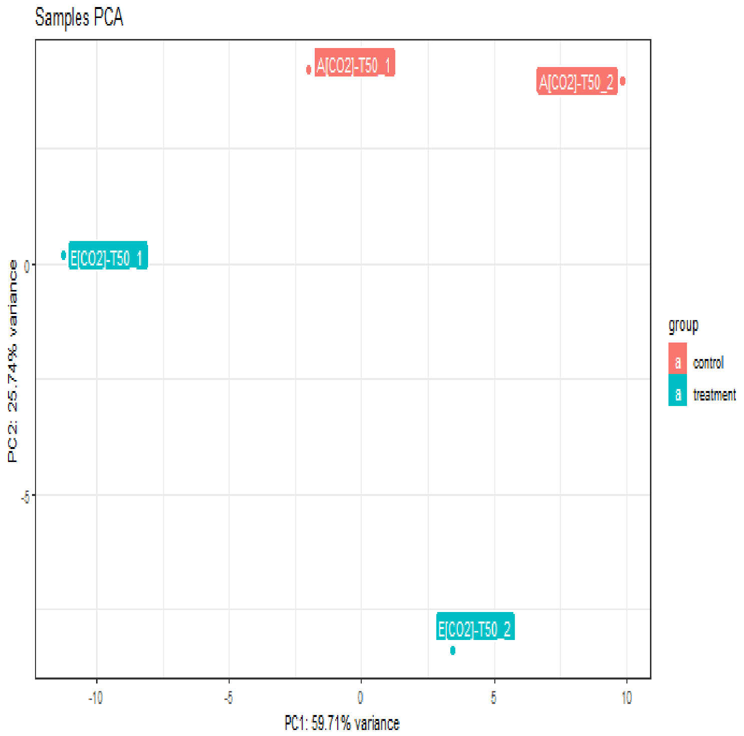

Ab10 - SRP064384_effectCO2_50minDrought

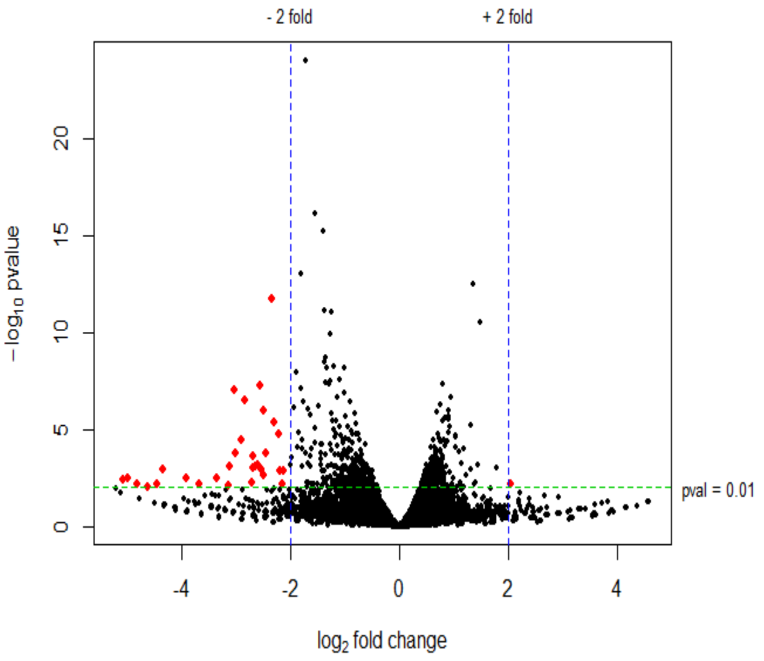

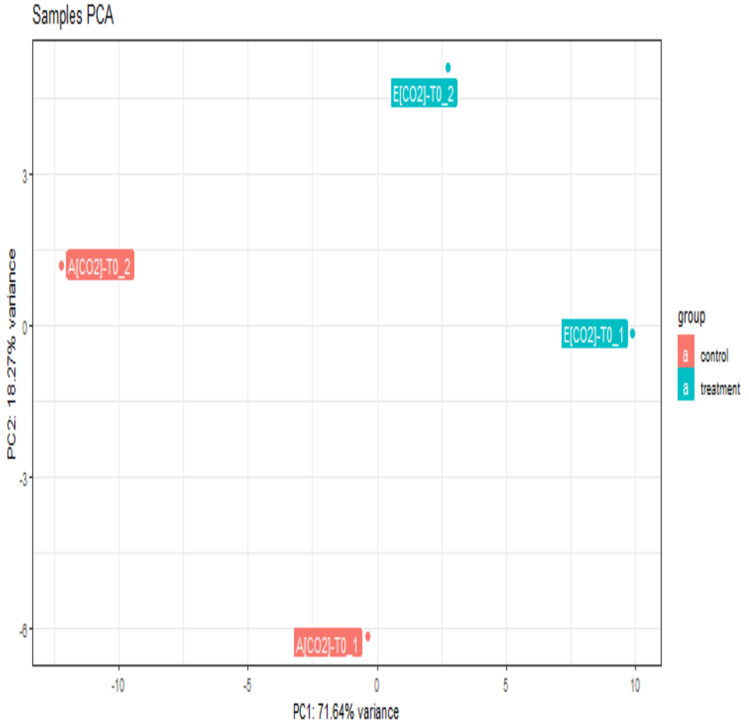

Ab10 - SRP064384_effectCO2_NoDrought

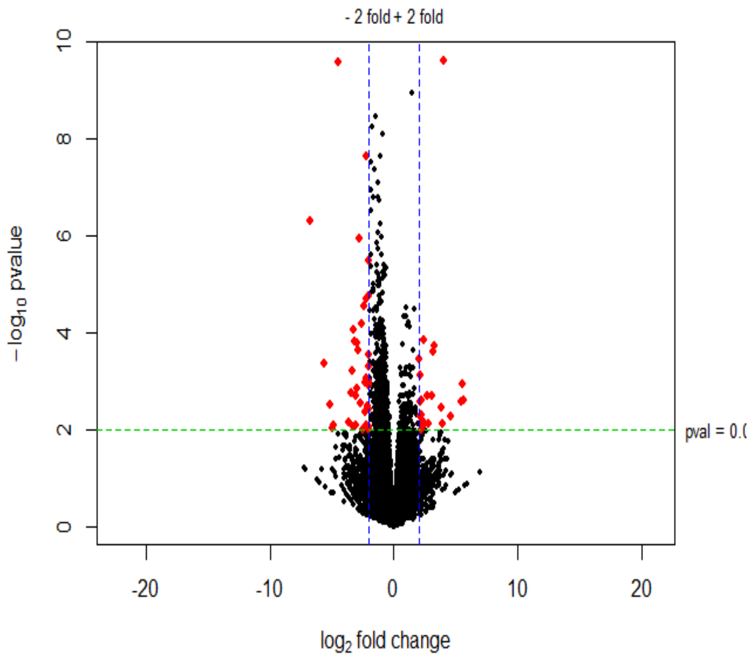

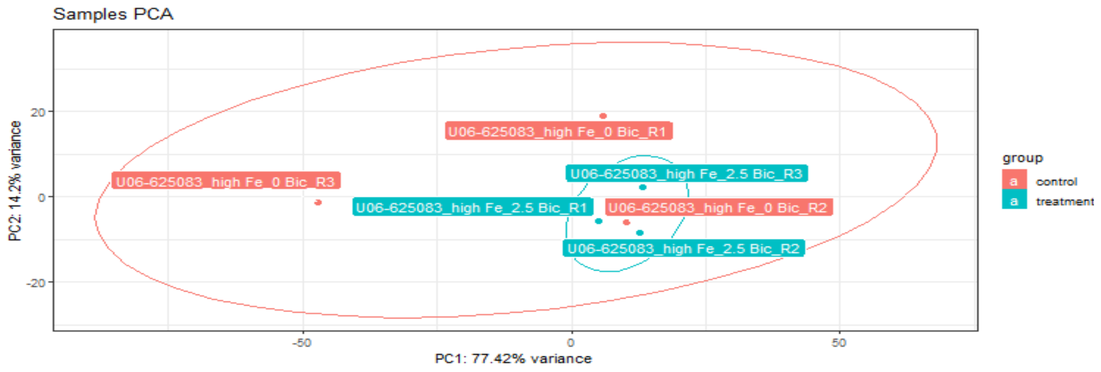

Ab13 - SRP108540_HighFe_2.5BIC_083

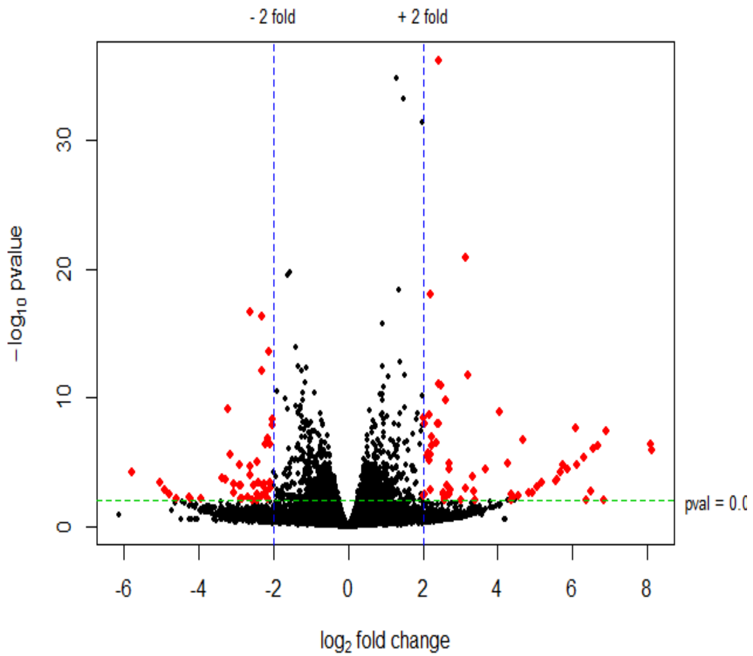

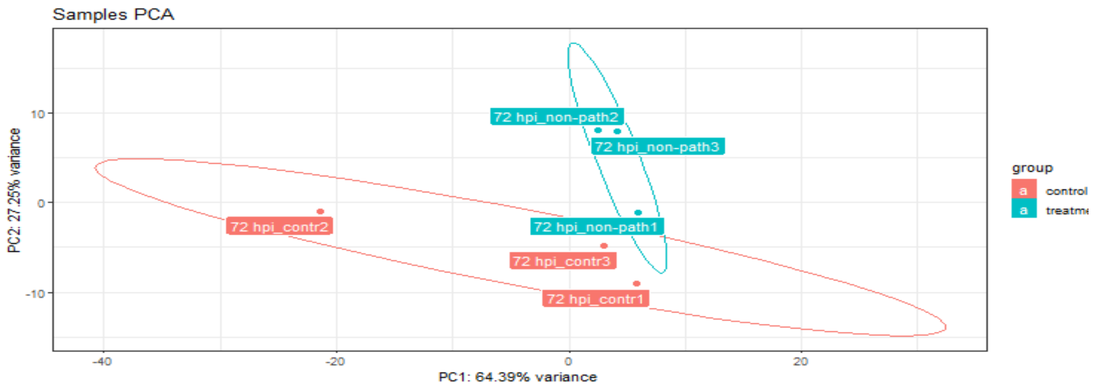

Bi2 - SRP056137_FO_nonpath72hpi

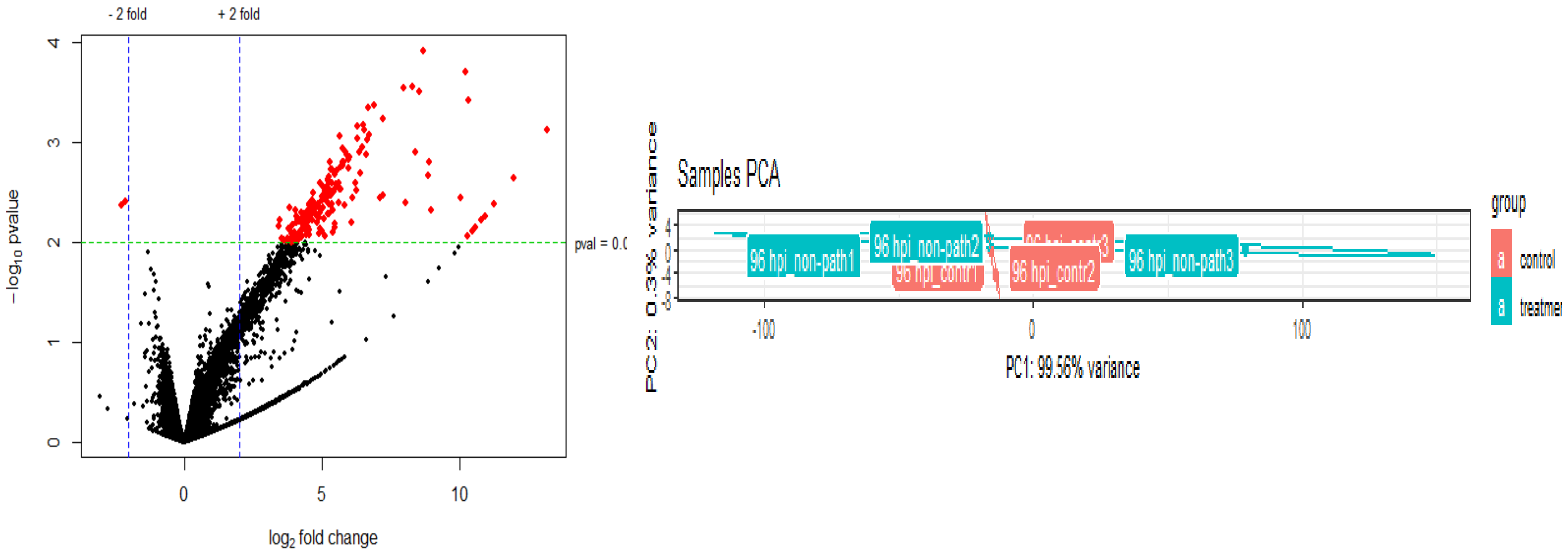

Bi2 - SRP056137_FO_nonpath96hpi

(**B**)

Figure S3. (**A**) Volcano plots and PCA plot of the samples (treatment versus control) for experiments that **passed** the quality control step to check for any con-founding factors within each DESeq2 dataset. For the volcano plots the X-axis corresponds to the log2Fold change and the Y-axis to the –log10 p-value for each gene within a given experiment and is drawn after DESeq2 analysis. The DESeq2 object is also used for a subsequent PCA analysis for each abiotic of biotic experiment used for re-analysis of which the % variance is given for each component (X- and Y-axis) and clustered by sample condition. **(B)** Volcano plots and PCA plots of the samples (treatment versus control) but for experiments that **did not pass** the quality control step to check for any con-founding factors within each DESeq2 datasets. This includes experiments with a relatively low amount of differential expression or experiments with a irregular volcano plot, the latter indicating that something went wrong with the sample.
